# β-arrestin recruitment facilitates a direct association with G proteins

**DOI:** 10.1038/s41467-026-74944-7

**Published:** 2026-07-17

**Authors:** Claudia Y. Lee, Jeffrey S. Smith, Taylor Kohlmann, Emily M. Meara, Uyen Pham, Frank Kwarcinski, Andrew N. Dates, Jessica K. Orofino, Issac Choi, Ari S. Hilibrand, Chanpreet Jassal, Abigail Gilmer, Stephen C. Blacklow, Gregory G. Tall, Andrew C. Kruse, Sudarshan Rajagopal

**Affiliations:** 1 Department of Biochemistry, Duke University School of Medicine, Durham, NC USA; 2 Department of Medicine, Duke University Medical Center, Durham, NC USA; 3 Department of Dermatology, Brigham and Women’s Hospital, Boston, MA USA; 4 Department of Biological Chemistry and Molecular Pharmacology, Harvard Medical School, Boston, MA USA; 5 Trinity College, Duke University, Durham, NC USA; 6 Department of Pharmacology, University of Michigan Medical School, Ann Arbor, MI USA; 7 Department of Cancer Biology, University of Cincinnati College of Medicine, Cincinnati, OH USA

**Keywords:** Hormone receptors, Receptor pharmacology, G protein-coupled receptors, Supramolecular assembly

## Abstract

G protein-coupled receptors (GPCRs) are targets for almost a third of all FDA-approved drugs. GPCRs are known to signal through both heterotrimeric G proteins and β-arrestins. Traditionally these pathways were viewed as largely separable, with G proteins primarily initiating downstream signaling while β-arrestins modulate receptor trafficking and desensitization in addition to regulating their own signaling events. Recent studies suggest an integrated role of G proteins and β-arrestins in GPCR signaling, however the cellular and biochemical requirements for G protein:β-arrestin interactions remain unclear. Here, we show that G proteins and β-arrestins can directly interact. Through utilization of β-arrestin-biased receptors and artificially enforced β-arrestin relocalization, we demonstrate that recruitment of β-arrestin to the plasma membrane is sufficient to interact with the G protein Gαi. Using purified proteins, we show that Gαi directly interacts with β-arrestin. In addition, we find that Gαi family members differ in their degree of association with β-arrestin, and that a large degree of this selectivity resides within the alpha helical domain of Gαi. These findings delineate the cellular and biochemical conditions that drive direct interactions between G proteins and β-arrestins and illuminate the molecular basis for how they interact.

## Abstract (web summary)

GPCRs help cells sense and respond to their environment, with G proteins and β-arrestins serving as critical signaling effectors. Here, authors show that G proteins can directly bind β-arrestins and define key cellular and biochemical requirements for these interactions.

## Introduction

GPCRs are critical regulators of human physiology, allowing cells to detect and respond to a wide range of stimuli ranging from peptides and small molecules to odorants and photons of light. Canonically, GPCRs are known to initiate signaling through two primary signaling pathways (1) heterotrimeric G proteins and (2) β-arrestins^1–3^. In G protein signaling, a conformational change in a GPCR allows it to catalyze exchange of Gα-bound GDP for GTP. The activated Gα then dissociates from the receptor and from the Gβγ subcomplex. Gα and Gβγ proceed to regulate the activity of downstream signaling proteins such as adenylyl cyclase, inositol trisphosphate receptor, and protein kinase C, as well as modulate ion channel activity^1^. In the canonical β-arrestin pathway, GPCR activation results in phosphorylation of serine and threonine residues, primarily by GPCR Kinases (GRKs) on the flexible C-terminal tail and larger intracellular loops. Phosphorylation of intracellular residues promotes β-arrestin binding to the receptor core, which sterically prevents Gα binding to the receptor, leading to desensitization of G protein signaling^4,5^. This interaction also facilitates plasma membrane anchoring of β-arrestins, despite the β-arrestins lacking lipid membrane-anchoring post translational modifications^6^. In addition, β-arrestins facilitate receptor internalization and can directly scaffold several other proteins, such as Src, extracellular-signal regulated signal kinase (ERK), and Raf, to initiate β-arrestin-dependent signaling events^7–12^.

Ligands have been identified that preferentially activate either the G protein or the β-arrestin signaling pathways. It was thought that such ‘biased-agonists’ could reduce certain on-target, but unintended, adverse effects while enhancing therapeutic efficacy. For example, at the μ-opioid receptor (MOR), the G protein-biased ligand TRV130 (oliceridine)^13^ is now FDA-approved for the treatment of acute pain. In addition to ligand bias, some receptors display intrinsic signaling preferences, a phenomenon referred to as ‘receptor bias’. For instance, the atypical chemokine receptor ACKR3 preferentially engages β-arrestins over G proteins^14^.

Despite promising initial results, the clinical success of biased signaling strategies has been more limited than expected. In addition, as signaling assay sensitivity and specificity has improved, the extent of cross-talk between signaling pathways regulated by both G proteins and β-arrestins is now appreciated^15,16^. Numerous signaling studies suggest an interrelated role of G proteins and β-arrestins in executing the multifaceted signaling signature of each GPCR. For example, concurrent discoveries in 2009 found that prolonged binding of β-arrestin to the parathyroid hormone receptor type 1 or activation of thyroid stimulating hormone receptor in endosomes prolongs cAMP production, implicating β-arrestin-regulated signaling in promoting endosomal G protein activity^17,18^. Further evidence for coordinated G protein and β-arrestin signaling has come from structural studies using negative stain and cryo-electron microscopy, which revealed a signaling “megaplex” involving a β2-adrenergic receptor/vasopressin 2 receptor chimera simultaneously engaging Gαs at the receptor core and β-arrestin at the phosphorylated C-terminal tail^19,20^, as well as more recent structural evidence of ‘megaplexes’ facilitated by an allosteric ligand^21^. Gαs can also direct GRK isoform recruitment, thereby modulating β-arrestin recruitment and alter β-arrestin conformation in cells as assessed by β-arrestin biosensors^22^. Consistent with cross talk between β-arrestin and G protein signaling, studies have recently demonstrated that G proteins and β-arrestins can associate following activation of multiple GPCRs^23^. These complexes differed from previously reported G protein:β-arrestin cross talk as this association was specific for inhibitory G protein (Gαi), even among receptors which do not classically signal through Gαi. Prior studies primarily utilized cell-based assays (confocal microscopy, luciferase, and BRET-based assays) and multiple wild-type GPCRs to show that varying the expression of other G protein families (Gαs, Gαq, Gα12) did not promote the same robust interaction with β-arrestin observed with Gαi-family G proteins. Varying transfections conditions and the tags required to assess the interaction did not change interpretation. These prior studies focused on functional consequences of the interaction, showing that Gαi:β-arrestin complexes can coordinate with secondary effectors, such as ERK and clathrin adapter AP-2^23,24^ and localized primarily to the plasma membrane. Disrupting Gαi and β-arrestin led to decreases in ERK phosphorylation and decreases in cell migration.

In order to better understand and untangle the complex interplay of G protein and β-arrestin signaling, we sought to define the biochemical and cellular requirements for G protein:β-arrestin interactions. In cells, we found that recruitment of β-arrestin to the plasma membrane, either via β-arrestin-biased receptors or chemically inducible heterodimerization, was both necessary and sufficient to promote Gαi:β-arrestin complex formation. In contrast, activation of Gαi alone, using G protein-biased agonists or aluminum fluoride, did not induce complex formation. We confirm this by leveraging the FK506 Binding Protein (FKBP) and FKBP Rapamycin Binding domain (FRB) heterodimerization system as an artificial, receptor-independent method to translocate β-arrestin to the plasma membrane. Using purified proteins, we show that Gαi can directly interact with β-arrestin, and that this interaction is not dependent on the nucleotide state of the Gα subunit. We further demonstrate that β-arrestin binding does not modulate nucleotide exchange by Gαi proteins. Finally, we find that the extent of β-arrestin association varies between different Gαi isoforms and reveal that regions within the alpha helical domain plays a crucial role in β-arrestin binding in living cells. Together, our experiments reveal the biochemical and cellular conditions that promote direct interactions between G proteins and β-arrestins.

## Results

### The M3 muscarinic receptor facilitates Gαi:β-arrestin complex formation

**Fig. 1:**
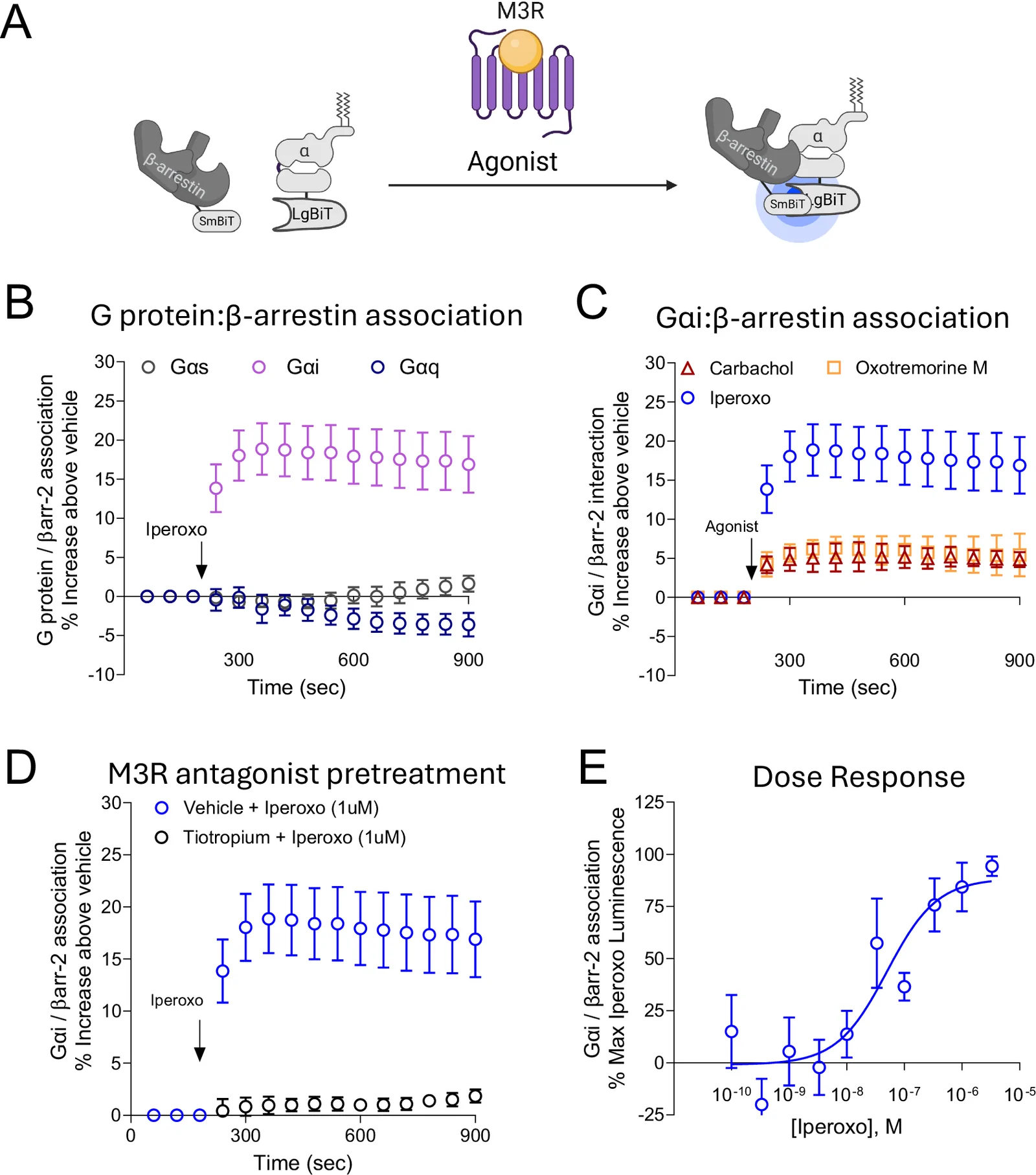
M3R activation promotes Gαi:β-arrestin association. **A** Cartoon of split luciferase assay. Created in BioRender. Lee, C. (2026) https://BioRender.com/03flw9d. **B** HEK 293T cells transiently transfected with the M3R, the indicated Gα-LgBiT, and SmBiT-β-arrestin-2 were treated with the muscarinic agonist iperoxo (1 µM). **C** Cells transiently transfected with the M3R, Gαi1-LgBiT, and SmBiT-β-arrestin-2 were treated with the indicated muscarinic agonist iperoxo (1 µM), carbachol (100 µM), or oxotremorine-M (10 µM). **D** Cells were pretreated with either tiotropium (10 µM) or vehicle for 10 min and subsequently treated with the indicated iperoxo (1 µM). **E** Cells transiently transfected with the M3R, Gαi1-LgBiT, and SmBiT-β-arrestin-2 were treated with iperoxo at the indicated concentration. *n* = 3 replicates per condition. Data shown are mean ± SEM. Source data are provided as a Source Data file.

We first re-established our previous findings of Gαi:β-arrestin and tested if agonist treatment of the M3 muscarinic receptor (M3R) could lead to G protein:β-arrestin complex formation in HEK293T cells by NanoBit complementation. M3R primarily signals through the G protein Gαq, but can also signal through Gαi and Gαs^25–27^. Agonist treatment of the M3R facilitated observable and robust associations of β-arrestin with Gαi, but not Gαq nor Gαs in cells (Fig. 1A, B). Multiple muscarinic agonists facilitated Gαi:β-arrestin complex formation (Fig. 1C), with their ability for promote complex formation directly correlated with their ability to recruit β-arrestin (Supplementary Fig. 1A, B). Gαq-LgBiT, Gαs-LgBiT, and Gα-LgBiT constructs were found by western blot to be similarly expressed (Supplementary Fig. 1C, D). Pretreatment with the muscarinic antagonist tiotropium prevented complex formation and increasing concentrations of agonist increased Gαi:β-arrestin association, indicating a receptor-specific effect (Fig. 1D, E).

### β-arrestin-biased GPCRs promote Gαi:β-arrestin complex formation

**Fig. 2:**
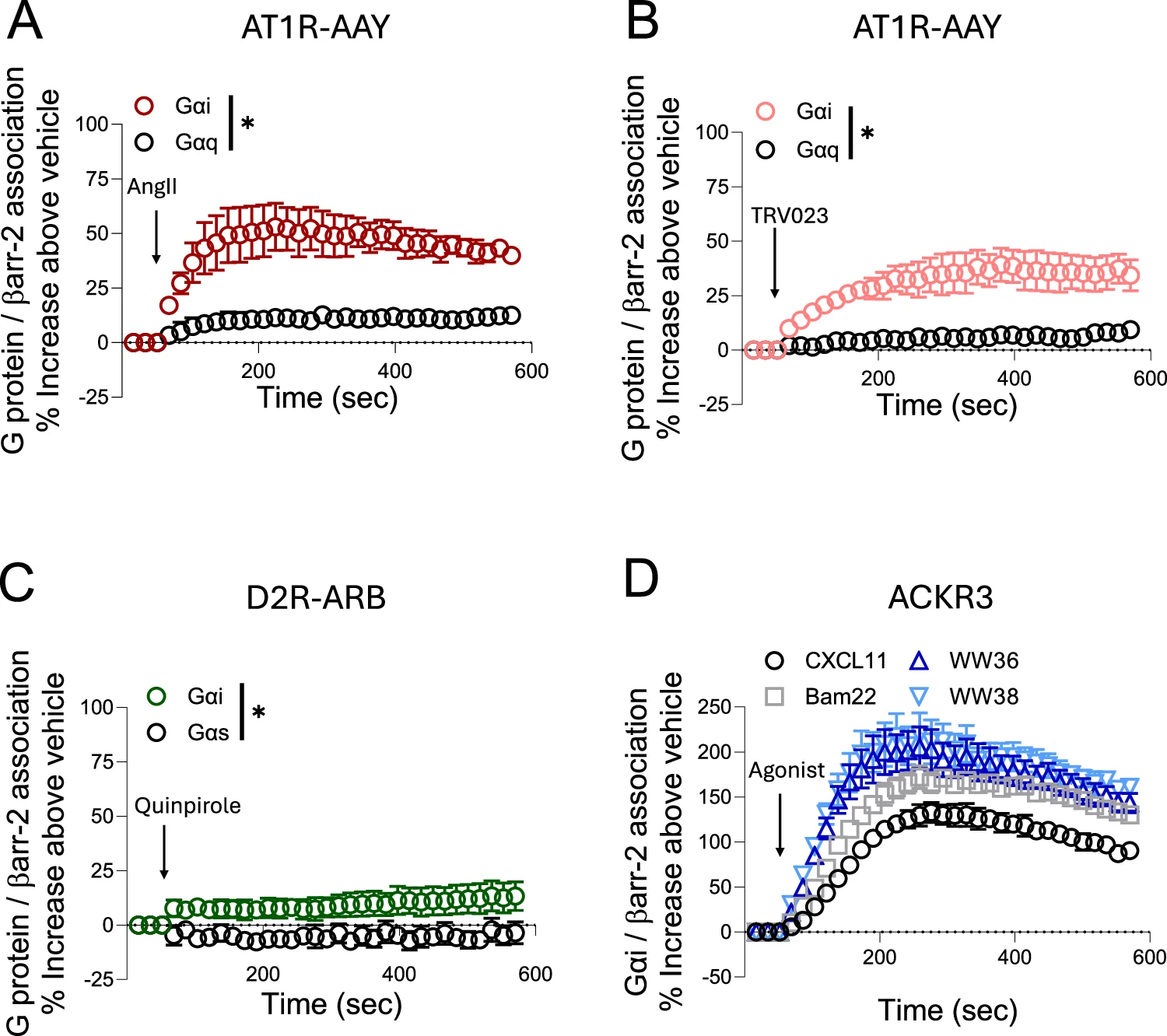
β-arrestin-biased GPCRs promote Gαi:β-arrestin association. HEK 293T cells transiently transfected with the indicated β-arrestin-biased receptor, the indicated Gα-LgBiT, and SmBiT-β-arrestin-2 **A** AT1R-AAY stimulated with 1 µM AngII **B** AT1R-AAY stimulated with 10 µM TRV023. **C** D2R-ARB stimulated with 1 µM Quinpirole. **D** ACKR3 stimulated with 100 nM of its endogenous ligand, CXCL11 (C-X-C Ligand 11), 1 µM Bam22, 1 µM WW36, and 1 µM WW38. For **A**, **B** *P < 0.0001 by two-tailed t test comparing area under curve (AUC) for Gαi-AngII versus Gαq-AngII (**A**) and Gαi-TRV023 versus Gαq-TRV023 (B). For **C** *P = 0.0014 Gαi versus Gαs by two-tailed t test comparing AUC. *n* = 3 for all conditions and represents independent plate replicates. Graphs depict the percent increase over vehicle treatment. Data shown are mean ± SEM. Source data are provided as a Source Data file.

The current paradigm for GPCR activation generally posits that the receptor active state confers conformational changes in both G proteins and β-arrestin to initiate their signaling. However, it is also known that G proteins and β-arrestins may have receptor-independent mechanisms of activation^28–30^. To assess whether Gαi activation was required for Gαi:β-arrestin association, we utilized three β-arrestin-biased receptors that are largely incapable of activating G proteins, including Atypical Chemokine Receptor 3 (ACKR3), Angiotensin II type I Receptor DRY mutant (AT1R-AAY), and a β-arrestin-biased Dopamine 2 Receptor L123W:R132L mutant (D2R-ARB)^14,31,32^. Both AT1R-AAY and D2R-ARB contain mutations in their DRY motifs (D125A and R126A in AT1R and R132L in D2R) that disrupt G protein activation, causing them to signal primarily via β-arrestin-mediated pathways^31,32^. D2R-ARB contains a L123W transmembrane mutation in addition to its DRY motif mutation (R132L) which allows for similar β-arrestin recruitment to native D2R receptors while lacking G protein recruitment and activation. Prior studies show that this mutant does not recruit Gαi1, Gαi2, Gαi3, or Gαo^31^. AT1R classically signals primarily through Gαq, and Gαq was tested alongside Gαi in complex formation experiments for comparison. The D1R classically signals primarily through Gαs and D2R through Gαi, therefore Gαs was chosen for comparison in these experiments. Lastly ACKR3 is a naturally occurring β-arrestin-biased receptor^14,33^. For AT1R-AAY, we tested both endogenous ligand angiotensin II (AngII) and known β-arrestin-biased agonist, TRV120023 (TRV023)^34^. For ACKR3, we tested multiple ligands, including two small molecules that selectively target ACKR3^35,36^. Despite the lack of G protein activation at these receptors, we observed that each β-arrestin-biased receptor promoted Gαi:β-arrestin complex formation (Fig. 2A–D). Furthermore, we showed at ACKR3 these complexes form in the absence of G protein recruitment to the receptor, which we confirmed in both G protein dissociation and G protein recruitment assays with four different ligands (Supplementary Fig. 2). These results demonstrate that Gαi:β-arrestin complex formation can occur independently of classical receptor-mediated G protein activation.

### G protein-biased ligands fail to promote Gαi:β-arrestin complex formation

**Fig. 3:**
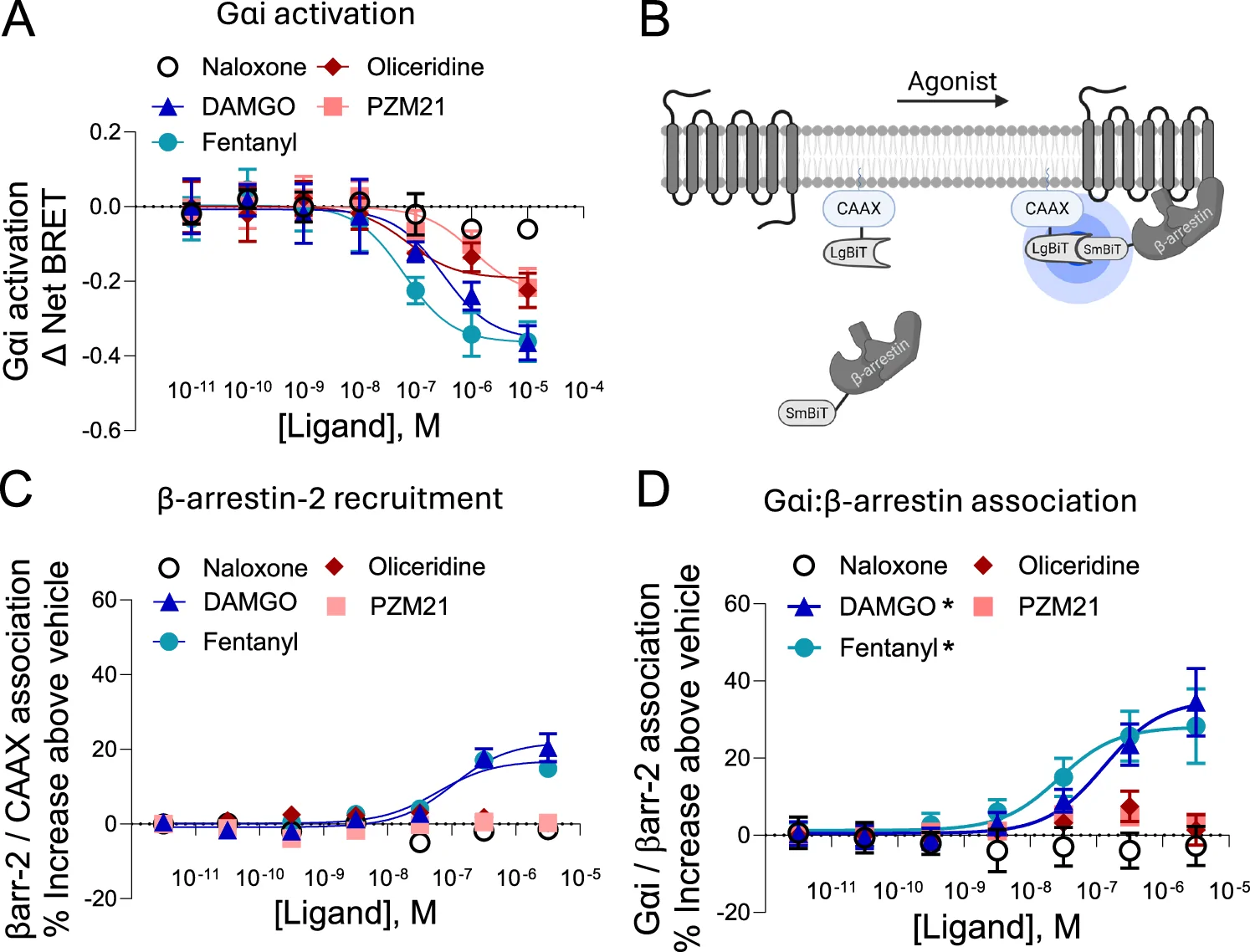
G protein-biased ligands do not promote Gαi:β-arrestin complex formation. **A** HEK293T cells overexpressing the Gi TRUPATH BRET sensor and 2000ng of MOR with (with treatment from DAMGO (*n* = 3), and Fentanyl (*n* = 3) or G protein biased ligands Oliceridine (*n* = 3) and PZM21 (*n* = 3). The antagonist Naloxone was used as a negative control (*n* = 3). **B** Illustration of bystander NanoBiT recruitment assay used to monitor β-arrestin-2 recruitment upon MOR activation. Created in BioRender. Lee, C. (2026) https://BioRender.com/k0sgbp3. **C** DAMGO (*n* = 6) and Fentanyl (*n* = 3), promote β-arrestin-2 recruitment to MOR whereas G biased ligands, Oliceridine (*n* = 5) and PZM21 (*n* = 5), do not. Naloxone was used as a negative control (*n* = 6) in (**C**). **D** Split luciferase Gαi-LgBiT:smBiT-β-arrestin-2 association upon stimulation with DAMGO (*n* = 9) and Fentanyl (*n* = 7), but not G protein biased ligands Oliceridine and PZM21 (*n* = 4). Naloxone (*n* = 7) was used as a negative control. For **D**, a two-way ANOVA with Dunnett’s post hoc test was performed to compare the effect of DAMGO, Fentanyl, Oliceridine, or PZM21 versus the negative control, Naloxone. *P < 0.0001 when comparing max signal DAMGO versus Naloxone and Fentanyl versus Naloxone. All graphs denote the mean signal ± SEM and represent percent increase over vehicle treatment. Source data are provided as a Source Data file.

To complement experiments with β-arrestin-biased receptors, we then tested whether activating the traditional G protein pathway without β-arrestin recruitment was sufficient to facilitate Gαi:β-arrestin association. The Gαi-coupled MOR has several well characterized agonists available including the balanced agonist [D-Ala2, N-MePhe4, Gly-ol]-enkephalin (DAMGO), fentanyl, and the G protein-biased agonists including oliceridine (also known as TRV130) and PZM21^37^. We first validated that oliceridine and PZM21 activated G proteins with minimal β-arrestin recruitment, while DAMGO and fentanyl promoted both G protein activation and β-arrestin recruitment (Fig. 3A–C). We proceeded to test these G protein-biased agonists for their ability to facilitate Gαi:β-arrestin complex formation. In contrast to DAMGO and Fentanyl which retained the ability to activate both G protein and β-arrestin pathways (Fig. 3D), oliceridine and PZM21 failed to promote Gαi:β-arrestin complex formation. These findings suggest that G protein activation alone is not sufficient for Gαi:β-arrestin complex formation and highlight the requirement of β-arrestin recruitment in mediating this interaction.

### β-arrestin recruitment to the plasma membrane is sufficient to form Gαi:β-arrestin complexes

**Fig. 4:**
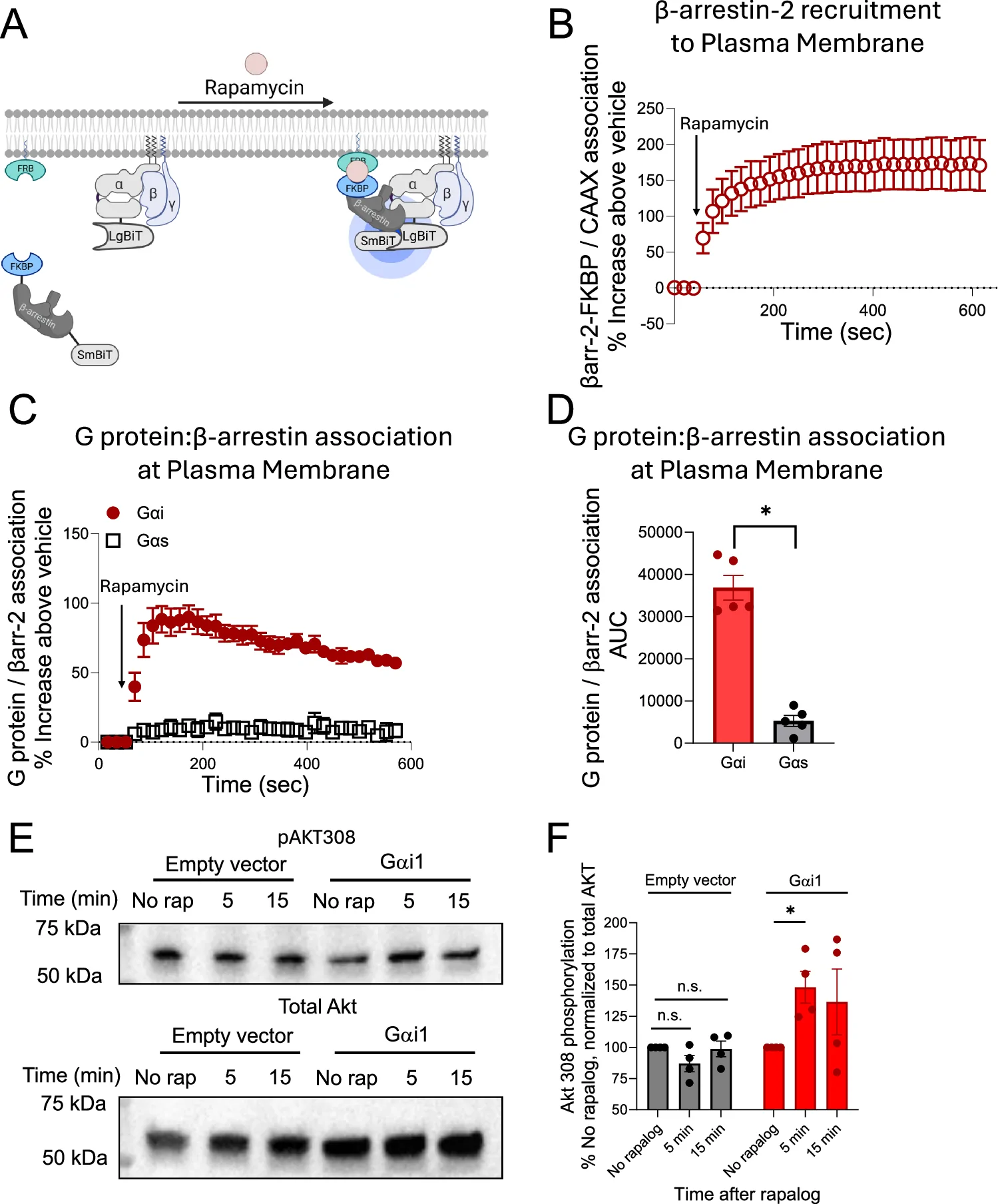
Translocation of β-arrestin to the plasma membrane is sufficient to promote Gαi:β-arrestin association and regulates Akt. **A** Schematic of NanoBiT assay monitoring Gαi:β-arrestin association upon induced translocation β-arrestin-2 to the plasma membrane. Upon addition rapamycin (500 nM), FKBP and FRB heterodimerize, forcing translocation of β-arrestin to the plasma membrane independent of receptor activation. Gαi:β-arrestin complexes are assessed via production of luminescence from reconstituted luciferases. Created in BioRender. Lee, C. (2026) https://BioRender.com/fj4dq48. **B** Recruitment of SmBit-β-arrestin-2-FKBP to the plasma membrane by heterodimerization with Lyn-FRB after 500 nM rapamycin stimulation. LgBiT-CAAX was used as a membrane marker. *n* = 4 and represents independently ran plate-assays. **C** Gαi:β-arrestin complex formation following addition of rapamycin. **C** AUC analysis of (**B**). *P = 0.0001 by two-tailed t-test with Gαi vs Gαs. *n* = 5 independent replicates for (**C**, **D**). **E** Representative blot of Akt Thr 308 phosphorylation after recruitment of SmBit-β-arrestin-2-FKBP to the plasma membrane via Lyn-FRB(LDR) after treatment with rapalog (500 nM) in HEK 293 G protein knockout cells and rescued with either empty vector or Gαi1. **F** Quantification of Akt Thr308 phosphorylation after recruitment of SmBit-β-arrestin-2-FKBP to the plasma membrane. *n* = 4 replicates. *P = 0.0255 by two-way ANOVA with Dunnett’s post hoc. Data shown are mean ± SEM. Source data are provided as a Source Data file.

Since β-arrestin-biased receptors, but not G protein-biased ligands, were capable of promoting Gαi:β-arrestin complex formation, we next tested if β-arrestin recruitment to the plasma membrane in the absence of GPCR agonist treatment was sufficient to form Gαi:β-arrestin complexes. To do this, we utilized a heterodimerization system comprised of FK506 Binding Protein (FKBP) and FKBP Rapamycin Binding domain (FRB) to force β-arrestin to the plasma membrane^8^. In this system we added a plasma membrane-localization Lyn11 tag to FRB and placed a FKBP onto the C-terminus of β-arrestin. In doing so we could translocate β-arrestin to the plasma membrane independently of GPCR activation upon the addition of rapamycin (Fig. 4A, B). Transiently transfecting these constructs in HEK293T cells followed by rapamycin treatment led to the formation of Gαi:β-arrestin, but not Gαs:β-arrestin complexes (Fig. 4C, D). We further adapted the FKBP/FRB system to probe if recruitment of β-arrestin-2-FKBP was sufficient to promote Akt phosphorylation (also known as protein kinase B), and if this was impacted by the presence of Gαi. Akt has previously been shown to be regulated by β-arrestin and control β-arrestin-dependent functions such as T cell chemotaxis^38,39^. Specifically, we overexpressed Lyn11-FRB and β-arrestin-2-FKBP in previously validated G protein knockout HEK 293 cells with Gαi rescue^23^. By utilizing a rapamycin analog, rapalog (AP21967), we minimized endogenous FKBP-FRB coupling^8,40^. Only upon rescue of Gαi and recruitment of β-arrestin-2-FKBP to the plasma membrane by rapalog did we observe Thr 308 phosphorylation of Akt (Fig. 4E, F). An alternative Akt phosphorylation site, Ser 473, was not significantly changed (Supplementary Fig. 3). Our data suggests plasma membrane-localized β-arrestin is sufficient to promote selective Gαi interaction even in the absence of GPCR agonism and that certain functional activities mediated by β-arrestin can require Gαi.

### β-arrestin directly interacts with Gαi

**Fig. 5:**
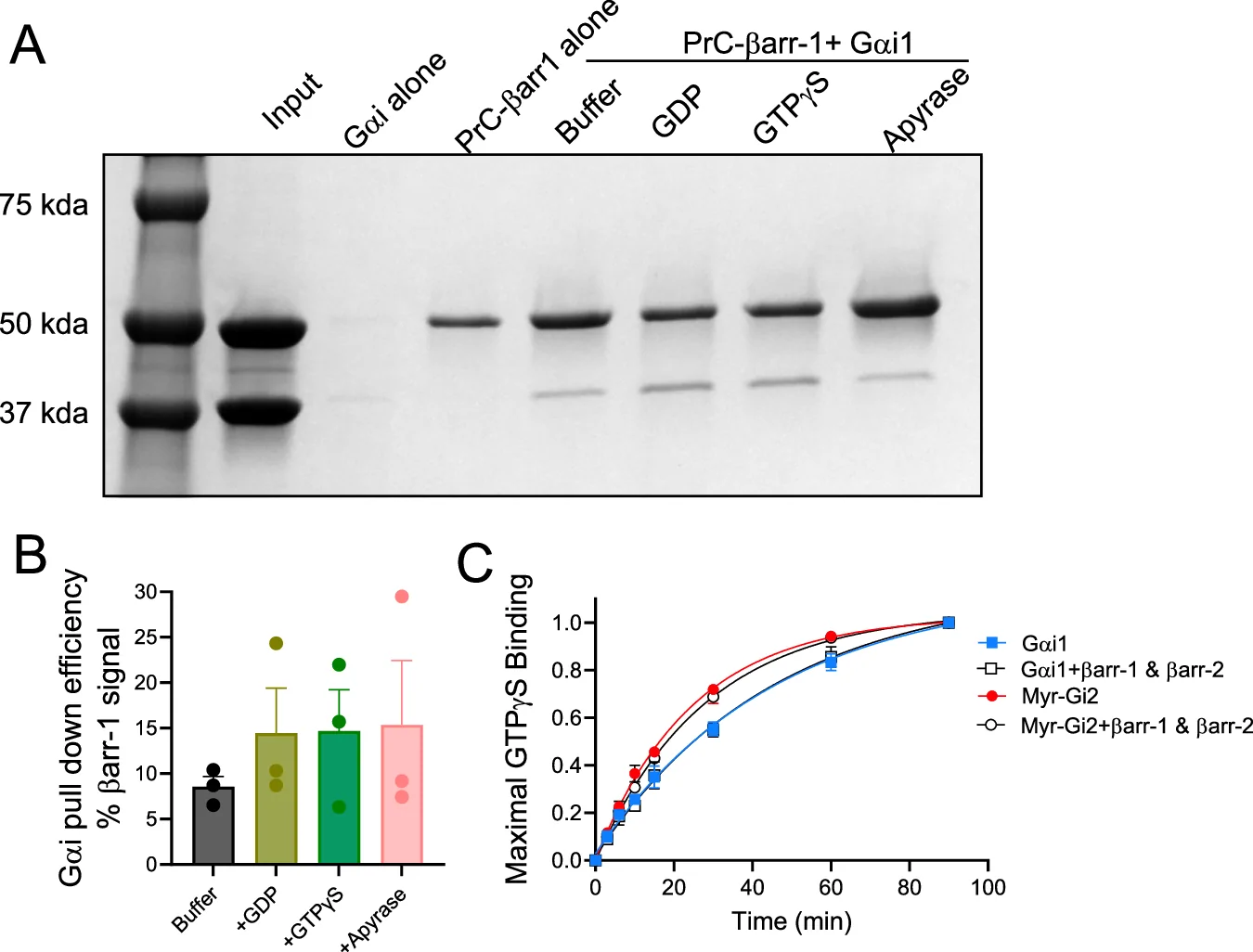
Purified Gαi and β-arrestin directly interact. **A** Coomassie gel of a pull down of purified N-terminally protein-c tagged β-arrestin-1-393X with purified non-lipidated Gαi1 incubated either with buffer or various nucleotide conditions (GDP 20 µM, GTPγS 20 µM, or apyrase for the nucleotide-free state). **B** Quantification of Gαi pulldown efficiency. **C** [^35^S]-GTPγS incorporation into non-lipidated Gαi1 or myristoylated Gαi2 in the presence or absence of 4x molar excess of purified β-arrestin-1-393X and β-arrestin-2-393X. For **A**, **B**, pull-down representative of three independent experiments. For **C**, *n* = 3 replicates are plotted as maximal GTPγS binding. Error bars are the mean ± SEM. Source data are provided as a Source Data file.

We then tested if purified β-arrestin and Gαi directly interact (chromatography traces in Supplementary Fig. 4). We utilized a C-terminally truncated form of β-arrestin-1 shown to have greater spontaneous receptor-binding activity (β-arrestin-1-393X) and a non-lipidated form of Gαi1. Pulldowns of the N-terminal Protein-C tag of β-arrestin led to the coelution of Gαi1, which was not markedly changed when treated with apyrase to remove nucleotides or incubated with excess GDP or GTPγS (Fig. 5A, B). These findings were consistent with our previous G protein-biased signaling data and consistent with NanoBRET studies where activation of G proteins by aluminum tetra-fluoride (AlF_4_^-^)^41^ or over expression of a guanine dissociation inhibitor of Gαi, Activator of G protein Signaling 3 (AGS3)^42^, did not facilitate Gαi:β-arrestin interactions (Supplementary Fig. 5). Given this direct interaction, we then tested if purified β-arrestin-1 or β-arrestin-2 could influence the rate of [^35^S]-GTPγS binding to lipidated or non-lipidated forms of Gαi. Basal Gαi [^35^S]-GTPγS binding rates were consistent with a previous study^43^. Consistent with the bound nucleotide status of Gαi not overtly influencing the ability to be pulled-down with β-arrestin-1, incubation with β-arrestin-1-393X and β-arrestin-2-392X did not alter the rates of [^35^S]-GTPγS binding to either myristoylated Gαi2 or unmodified Gαi1, consistent with the pulldowns in which Gαi:β-arrestin association was agnostic to nucleotide state of the Gαi protein (Fig. 5C).

### Mutagenesis reveals domains of Gαi critical for interaction with β-arrestin

To identify the structural domains within Gαi that are crucial for Gαi:β-arrestin we first looked at the Gα interface with Gβγ. This region is mainly comprised of the Gα Switch 2 region and is well known to bind several proteins including modulators for Gα activity like Ric-8A (resistance to inhibitors of cholinesterase)^44^ and Gα-interacting vesicle-associated protein (also known as Girdin)^30^ as well as downstream effectors like adenylyl cyclase^45^. To determine if Gβγ could compete with β-arrestin for binding to Gαi, we first assessed the effect of adding purified Gβγ to our pull down of β-arrestin-1 with purified Gαi1. We found no significant change in pulldown efficiency in the presence of additional Gβγ (Supplementary Fig. 6A, B). Furthermore, overexpression of Gβγ did not disrupt Gαi:β-arrestin complex formation in our NanoBiT assays (Supplementary Fig. 6C, D). These data suggest that Gβγ and β-arrestin do not compete for the same Gαi binding site and is consistent with our previous observation that Gαi does not require Gβγ for association with β-arrestin (Fig. 5A).

**Fig. 6:**
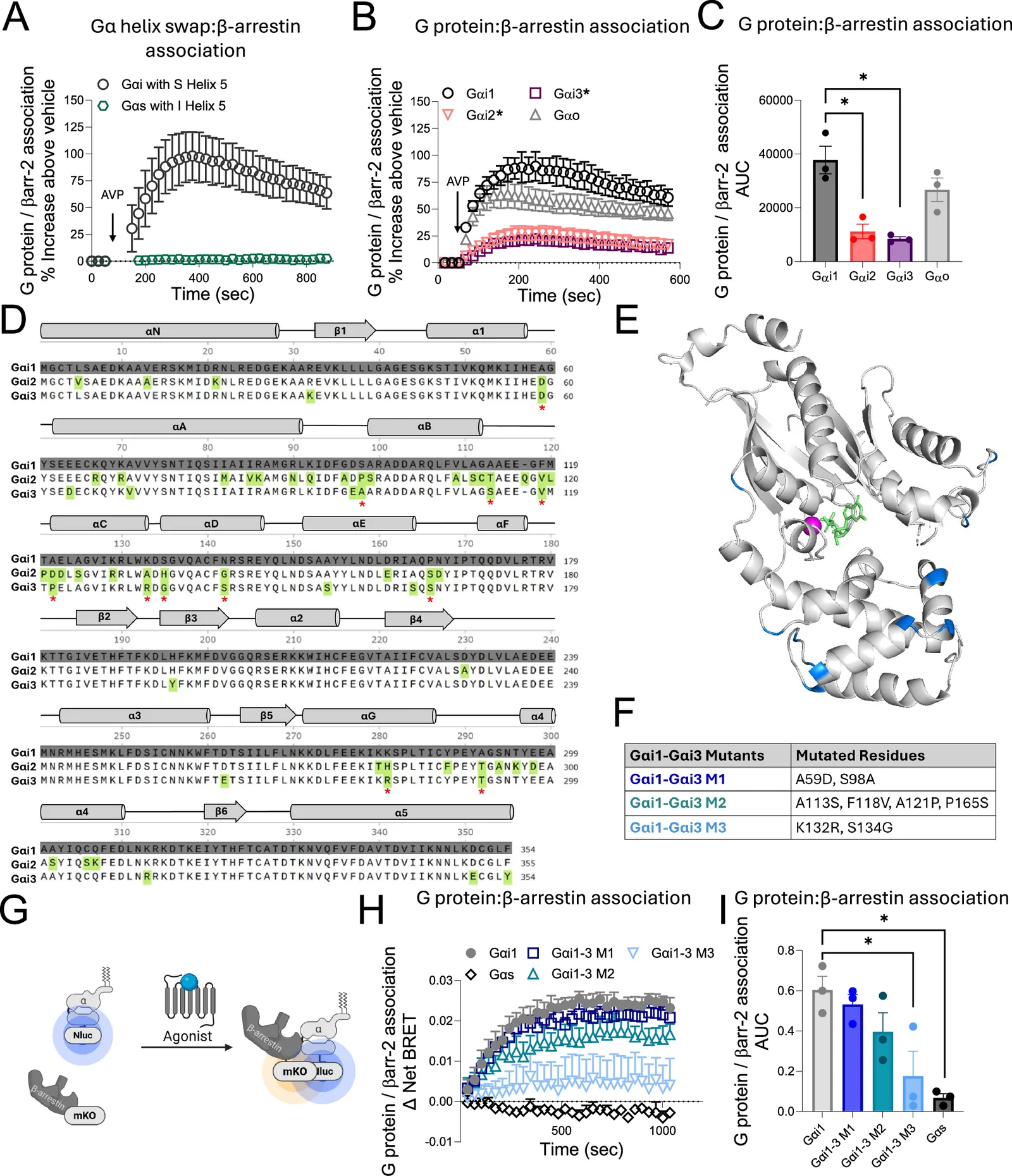
Mutations within Gα helical domain impairs interactions with β-arrestin. **A** HEK293T cells were transiently transfected with V2R, SmBiT-β-arrestin-2, and either LgBiT-tagged Gαi with Gαs α5 helix or LgBiT-tagged Gαs with Gαi α5 helix and treated with AVP (500 nM). **B** LgBiT-tagged Gαo, Gαi1, Gαi2 and Gαi3 were compared with their ability to interact with SmBiT-β-arrestin-2 in cells overexpressing the V2R and treated with AVP (500 nM). **C** Area under the curve graph (AUC) made from the kinetic tracing data in (**B**). *P = 0.0023 by one-way ANOVA, Dunnett post hoc test of Gαi1 versus Gαi2, *P = 0.0012 by one-way ANOVA, Dunnett post hoc test of Gαi1 vs Gαi3. **D** Sequence alignment of human Gαi1, Gαi2, Gαi3 with differing residues highlighted with red asterisks. Alignment performed by GproteinDb^69,70^. **E** Structure of inactive Gαi1 (PDB:1BOF^71^) residues distinct between Gαi1 and Gαi2 and Gαi3 are highlighted in green. **F** Table of Gαi1 to Gαi3 mutants made: Gαi1 A59D, S98A (M1), Gαi1 A113S, F118V, A121P, P165S (M2), or Gαi1 K132R S134G (M3). **G** Diagram of the NanoBRET assay for Gαi:β-arrestin complex formation. HEK293T cells were transiently transfected with Nluc-tagged Gαi mutants (WT, M1, M2, or M3), β-arrestin-2-mKO, and V2R. Upon activation of V2R with AVP (500 nM), cells were monitored for changes in BRET ratio indicating Gαi:β-arrestin complex formation. Created in BioRender. Lee, C. (2026) https://BioRender.com/s1o8gem. **H** Gαi: β-arrestin association assessed by NanoBRET following AVP stimulation. **I** Area under the curve graph (AUC) made from the kinetic tracing data in **H** and represents Δ Net BRET between AVP and vehicle treated cells. *P = 0.0026 by one-way ANOVA with Dunnett’s post hoc comparing Gαs versus Gαi1 and *P = 0.0114 by one-way ANOVA with Dunnett’s post hoc comparing Gαi1-3 M3 versus Gαi1. Data in **A**, **B** demonstrate % increase luminescence of AVP above vehicle signal. All data shown are from *n* = 3 replicates. Data shown are mean ± SEM.

We next probed the C-terminal α5 helix, known for its key role in receptor binding and selectivity^46^. We generated chimeras of Gαi and Gαs, which we pervious observed no association with β-arrestin, by swapping the α5 helix of Gαi into Gαs (Gαs-i-helix5) and vice versa (Gαi-s-helix5). Exchange of helix 5 had no effect on β-arrestin binding by either G protein, as evidenced by clear complex formation by Gαi-s-helix5, but not the Gαs-i-helix 5 (Fig. 6A). These helix swap chimeras are functional as assessed by the ability to dissociate from βγ following agonist treatment of either the canonically Gs-coupled V2R or canonically Gi-coupled CXCR3 (Supplementary Fig. 7A–D). These data show that the Gαi α5 helix is not sufficient for its interaction with β-arrestin.

To identify other domains within Gαi that could interact with β-arrestin we looked for differences in β-arrestin binding amongst the closely related members of the Gαi subfamily. Upon activation of V2R, we found Gαi1 and Gαo formed more robust complexes with β-arrestin compared to Gαi2 and Gαi3, which displayed reduced interaction (Fig. 6B, C). We further confirmed that expression differences between wild-type G proteins and chimeras did not correlate with the ability to form complexes with β-arrestin (Supplementary Fig. 7E–I). Based on these findings, we hypothesized that residues which differ between Gαi1 and those in Gαi2 and Gαi3 are important to promote Gαi:β-arrestin complexes. To determine which regions potentially contributed to this difference, we aligned the amino acid sequence of Gαi1, Gαi2, and Gαi3. Upon initial inspection, there were a total of 11 residues which differed between Gαi1 and those of Gαi2 and Gαi3. Several of these residues were located within the alpha helical domain of Gαi1 (Fig. 6D, E), suggesting that this region of the protein might confer specificity for interactions with β-arrestin. In fact, the alpha helical domain is a known binding site for Regulator of G Protein Signaling (RGS) proteins, which selectively generate distinct RGS-G protein pairs via residue differences across Gαi isoforms in this region^47,48^. To test if these differing residues within Gαi subfamily members conferred the subtype effect for Gαi:β-arrestin, we generated three Gαi mutants in which non-conserved helical domain residues in Gαi1 were substituted with those from Gαi3. These point mutants were grouped by their spatial proximity within Gαi1 structure and linear sequence location and were referred to as Gαi1-Gαi3 M1, Gαi1-Gαi3 M2, and Gαi1-Gαi3 M3 (Fig. 6E, F). These mutants were tagged with a full-length luciferase (NanoLuc) for use in a NanoBRET assay and to ensure expression differences did not account for variation in signal. Upon treating the V2R with agonist, we observed that Gαi1 K132R, S134G (Gαi1-Gαi3 M3) within the alpha helical domain greatly decreased the association with β-arrestin (Fig. 6F–I, Supplementary Fig. 7J). These results highlight the importance of specific residues within the helical domain of Gαi1, such as K132 and S134, for β-arrestin binding. Given that RGS proteins utilize the same residues in their helical domain for G protein binding and confer specificity by the orientational differences of side chains^48^, we further tested whether the entire helical domain is required for interaction with β-arrestin. To do this, we generated a Gαi-Gαs helical domain swap (Gαi-GαsHD) and evaluated Gα:β-arrestin complex formation using the NanoBiT complementation assay. The Gαi-GαsHD had diminished ability to interact with β-arrestin. However, the addition of GαiHD to Gαs (Gαs-GαiHD) was insufficient to restore measurable interaction with β-arrestin, suggesting some additional components within the Ras domain is required (Supplementary Fig. 8A–C). Both helical domain swaps retained ability to dissociate with their Gβγ counterpart, albeit to a lower degree than wild type Gαi and Gαs (Supplementary Fig. 8D–G). The totality of mutagenesis experiments suggested that the alpha helical domain of Gαi is a critical, but not sufficient, determinant for β-arrestin interaction.

## Discussion

In this study, we identified the cellular and certain biochemical factors that promote direct interactions between G proteins and β-arrestins. By using β-arrestin-biased receptors and the FKBP/FRB heterodimerization system, we showed that recruitment of β-arrestin to the plasma membrane was sufficient to promote its interaction with G proteins, even in the absence of receptor activation. Activation of G protein alone, either through G protein-biased agonists or aluminum fluoride, was not sufficient to promote significant G protein:β-arrestin association. In cells, we found that this interaction was most robust with Gαi-family proteins relative to other G protein families. With purified proteins, we showed that β-arrestin directly interacts with Gαi and does not require Gβγ. This interaction did not depend on the nucleotide status of the G protein, as Gα in the GDP-bound, GTP-bound, or nucleotide-free states were all capable of interacting with β-arrestin. Consistent with these observations, purified β-arrestins did not alter the rate of GTP binding to Gαi. Sequence alignment of Gαi-family isoforms and subsequent mutagenesis studies localized a likely interaction site with β-arrestin to the alpha helical domain of Gα. It is also clear that there is some contribution from the Ras domain. This is supported by other work utilizing hydrogen-deuterium exchange mass spectrometry where both Ras and helical domain of Gαi contributed to interactions with β-arrestin with Gαi-HD having higher affinity^49^. We also found that the C-terminal α5-helix of Gαi, which engages GPCRs, did not confer specificity for Gαi:β-arrestin. This further supports our findings that G proteins interact with β-arrestins in a manner distinct from their binding to GPCRs. While the absence of clear β-arrestin association with either Gαs or Gαq in HEK293 cells is consistent with prior observations^23,24^, we note that the absence of observed association in our assays does not preclude association under other conditions or in other cell types, as other G protein family members have been observed in close proximity with β-arrestin via crosslinking or in the presence of a GPCR^19,50^.

Our data supports a working model where plasma membrane-localized β-arrestin can directly interact with the alpha helical domain of Gαi, independent of a GPCR scaffold. β-arrestin did not provide direct guanine exchange factor (GEF) activity as assessed by the measuring the velocity of GTP binding to Gαi. Such an interaction that does not necessarily require a GPCR is consistent with an ‘action-at-a-distance’ signaling model of β-arrestin previously described by Von Zastrow and colleagues^51^, and seemingly consistent with observations of β-arrestin and Gαi in endosomes that lack an associated receptor^52^. Available structural and functional data suggest that β-arrestins adopt a variety of conformational states and binding partners depending on the cellular compartment, function, and lipid environment^53–56^. Indeed, it is now appreciated that endosomal or organellar signaling initiated by G proteins leads to fundamentally distinct cellular responses^57,58^. Consistent with this, we show that the presence of Gαi enhances Akt phosphorylation of Thr 308 when β-arrestin is recruited to the plasma membrane using the FRB/FKBP system. It is clear G proteins alone are sufficient to activate certain kinase signaling cascades in the absence of β-arrestins^59^, and evidence also points to this ability in certain scenarios for β-arrestins in the absence of G proteins^60^. We speculate that β-arrestins can help facilitate trafficking and localization of G proteins to various intracellular compartments, thus further tuning cellular responses, although further work is necessary to test this hypothesis.

There are limitations of our study which should be considered. Many of our experiments utilized overexpressed proteins which may alter the basal signaling processes that occur in our cell system. In some cases, such overexpression can result in diminished signaling specificity downstream of GPCRs^61^. Utilizing signaling assays with limited amplification, such as BRET-based approaches for measuring G protein activation and β-arrestin recruitment, relative to other assays that rely on signal amplification (e.g., Glosensor or PRESTO-TANGO), may not capture weak interactions with receptors and thus may explain differences in overall assessments of biased signaling between studies. Thus, there may be additional cellular components or cofactors which may have been overlooked in our assessment that can promote, or inhibit, G protein and β-arrestin interactions.

Mounting evidence links the spatial and temporal localization of receptors, G proteins, and β-arrestins to divergent signaling events and physiological responses^17,57^. The physiological relevance of such a direct Gαi: β-arrestin interaction is supported by a growing number of reports. For example, the Gαi: β-arrestin interaction is thought to be critical to follicle stimulating hormone function (a receptor that classically couples to Gαs)^62^. Gαi: β-arrestin comes in close contact to the AP2 adapter complex^24^. Blocking the interaction can impair ERK phosphorylation after GPCR agonist treatment, although interestingly not through all receptors^23,24^. The current evidence suggests that Gαi:β-arrestin interactions can facilitate important cellular signaling events, but that other GPCR signaling pathways independent of this Gαi: β-arrestin interaction are clearly critical for many GPCR signaling transduction events, as certain responses are retained in the absence of β-arrestin.

Taken together, our findings provide an important advance in understanding the mechanistic interplay between G protein and β-arrestin transduction pathways. Further experiments are needed to define how this direct association between two critical mediators of GPCR signaling regulates specific cellular pathways and cellular functions.

## Methods

### DNA plasmid generation

Constructs were generated via conventional cloning methods including restriction digest cloning and overlap cloning (see Supplementary Tables 1–3). G protein mutants were generated using Quikchange Lightning mutagenesis (Agilent). G protein helix 5 swaps were cloned by overlap extension amplification with Sigma Ultramer containing the entire helix 5 of Gαi. Gαi1-Nanoluc were synthesized by IDT gBlock (Integrated DNA Technologies, Inc). AHD swaps were synthesized by IDT gBlock (Integrated DNA Technologies, Inc) for Rluc8 constructs and overlap extension amplification for NanoBiT constructs. All constructs that were created, purchased, and received as gifts are denoted in acknowledgements.

### Bacterial strains

XL-1 Blue ultracompetent *E. coli*, (Agilent), XL-10 Gold ultracompetent *E. coli* (Agilent) and NEB 5-alpha competent *E. coli* (New England Biolabs) were used to express and clone constructs.

### Cell culture and transfection

HEK293T cells were maintained in minimum essential medium containing 10% fetal bovine serum and 1% penicillin-streptomycin. Cells were stored in humidified incubator and grown at 37 °C. Cells were transiently transfected by an optimized calcium phosphate^63^, Fugene, or polyethylenimine (PEI) method^64^ as previously described. For the calcium phosphate transfection, cell culture media was replaced 30 minutes prior to transfection. DNA constructs were diluted into 90 µL of sterile water. Afterwards, 10 µL of 2.5 M calcium chloride was added to the diluted DNA and mixed. Next, 100 µL of 2xHEPES-buffered saline solution (10 mM D-Glucose, 40 mM HEPES, 10 mM potassium chloride, 270 mM sodium chloride, 1.5 mM disodium hydrogen phosphate dihydrate) was added to the solution dropwise, incubated for 30 s–1.5 min, and then added to the cells. For Fugene or PEI transfections, a 3:1 ratio of DNA to Fugene or PEI (w/w) was utilized. Briefly, DNA constructs were diluted to 100 µL in Opti-MEM (GIBCO). The appropriate amount of PEI was diluted to 100 µL in Opti-MEM and added to the DNA Opti-MEM. The DNA:PEI solution was mixed, incubated at room temperature for 15–20 min, and then added to cells. All transfections were carried out in 6-well cell culture dishes (Costar) unless otherwise denoted.

### NanoBiT assays and FKBP-FRB Heterodimerization

Experiments were performed as previously described^23^. HEK293T cells were transiently transfected using calcium phosphate. For Gαi:β-arrestin complex formation assays, 250–500 ng of smBiT-βarrestin1, smBiT-βarrestin2, 100–200 ng of Gα-LgBiT, and 1000–2000 ng of untagged receptor were used. For recruitment assays with SmBiT-βarrestin2 or Gα-LgBiT, 500–2000 ng of LgBiT or SmBiT tagged receptor was utilized respectively. 18–24 h post transfection cells were swapped into a starvation medium (clear minimal essential media (MEM) supplemented with 1% penicillin and streptomycin, 1% anti-mycotic anti-biotic, 1% FBS, 10 mM HEPES, 1% sodium pyruvate) and seeded into a white, clear bottom, 96-well plate at 50,000–100,000 cells/well. After overnight incubation, starvation medium was removed and replaced with 80 μL of assay buffer containing 20 mM HEPES and 3 μM of coelenterazine-h (NanoLight Technologies) diluted into Hanks Balanced Salt Solution buffer without calcium or magnesium (HBSS). After 15 min, cells were analyzed for basal luminescence at 485 nm for 3 reads at 0.2 ms per well in a BioTek Synergy-Neo2 plate reader set to 37 °C. Following baseline reads, cells were stimulated with either 20 μL of 5X ligand diluted in the assay buffer, or 20 μL of vehicle (assay buffer) and then read for an additional 30 reads. For NanoBiT assays, the net change in luminescence was analyzed as a percent increase over vehicle treatment and normalized to basal luminescence reads. Some assays were additionally normalized to max signal of indicated treatment group.

For NanoBiT FKBP-FRB heterodimerization assays, cells were transfected via 3:1 PEI transfection with 1000 ng Lyn-FRB, 500 ng of SmBiT-β-arrestin2-FKBP and 100–200 ng Gα-LgBiT. For recruitment verification, 500 ng of CAAX-LgBiT was used along with the previously reported amounts of Lyn-FRB and SmBiT-β-arrestin2-FKBP 18–24 h post transfection cells were swapped into a starvation medium and seeded into a white, clear bottom, 96-well plate at 50,000–100,000 cells/well. After overnight incubation, starvation medium was removed and replaced with 80 μL of assay buffer containing 20 mM HEPES and 3 μM of coelenterazine-h diluted into Hanks Balanced Salt Solution buffer without calcium or magnesium (HBSS). After 15 min, cells were analyzed for basal luminescence at 485 nm for 3 reads at 0.2 ms per well in a BioTek Synergy-Neo2 plate reader set to 37 °C. Following baseline reads, cells were stimulated with either 20 μL of 5 × 2.5 µM Rapamycin stock diluted in the assay buffer as previously described^29^, or 20 μL of vehicle (assay buffer) and then read for an additional 30 reads. The net change in luminescence was analyzed as a percent increase over vehicle treatment and normalized to basal luminescence reads. Plates were read at 37 °C with the standard Rluc 485 nm emissions filter with 3 baseline reads followed by 30 post-stimulation reads. Rapamycin treated cells were given 500 nM Rapamycin final concentration.

For expression determination with HiBiT peptide, G protein LgBiT constructs were transiently expressed in HEK293T cells in the same amounts previously described for Gαi:β-arrestin as well a non-transfected control well. After an incubation for 18–24 h in 37 °C, 50,000 cells/well were seeded into 96-well plates and incubated overnight at 37 °C. The following day, cells were washed with PBS, lysed in the 96-well plate with Passive Lysis Buffer (Promega), and incubated with titrated amounts of purified HiBiT peptide (MedChemExpress) for 30 min at room temperature. Following HiBiT addition, coelenterazine-h diluted in assay buffer containing 20 mM HEPES was added to each well at a final concentration of 3 μM and incubated for 15 min. Cells were analyzed for luminescence at 470–480 nm for 3 reads at 0.35 ms per well in a Clariostar Plus plate reader set to 37 °C.

### Akt phosphorylation upon FKBP-FRB heterodimerization

Δ4 G protein knockout (GKO) cells were seeded in 6-well plates at a density of approximately 7E5 cells per well. Twenty-four hours after seeding, cells were transiently transfected using FuGENE according to the manufacturer’s instructions with plasmids encoding Lyn-FRB (1000 ng), SmBiT–β-arrestin-2–FKBP (500 ng), and either Gαi (200 ng) or pcDNA3.1 empty vector as a control. Forty-eight hours post-transfection, cells were serum-starved for 4 h and subsequently treated with rapalog (500 nM) for the indicated time at 37 °C to induce membrane recruitment of SmBiT–β-arrestin-2–FKBP. Cells were washed twice with ice-cold PBS and lysed in 150 µL RIPA buffer supplemented with protease and phosphatase inhibitors for ~1 h. Lysates were collected by centrifuging at 14,000 *g *× 15 min and stored at −80 °C prior to analysis. Proteins were resolved by SDS–PAGE using 4–20% gradient gels and transferred to nitrocellulose membranes. Membranes were blocked for 1 h in 5% BSA in TBST and incubated overnight at 4 °C with primary antibodies against phospho-Akt (Thr308) (Cell Signaling Technologies #2965), phospho-Akt (Ser473) (Cell Signaling Technologies #31957), or total Akt diluted in TBST containing 1% BSA (Cell Signaling Technologies #2920). Following three 10-min washes in TBST, membranes were incubated with HRP-conjugated anti-rabbit or anti-mouse secondary antibodies for 20–30 min at room temperature, washed three additional times, and visualized. Following imaging, phospho-Akt blots were stripped, washed with TBST, and reblotted with total Akt. For quantification, pixel intensity of the phosphor-Akt condition was quantified in Image Lab 6.1 and normalized to total Akt pixel intensity.

### BRET Assays and NanoBiT BRET assays

Experiments were performed as previously described^23^. Briefly, constructs were transiently transfected using calcium phosphate or PEI into HEK 293 T cells in a 6 well plate. For NanoBRET, 50 ng of Gα-Nanoluc, 500 ng β-arrestin-mKO, and 2000 ng of untagged receptor was used. For internalization assays, optimized amounts of DNA for MyrPalm-mVenus and receptor tagged with RlucII were used (amounts ranged from 500 to 1000 ng receptor-RlucII and 1000–1500 ng of MyrPalm-mVenus). For β-arrestin recruitment assays, β-arrestin-mKO and receptor-RlucII were used (amounts ranged from 500 to 1000 ng receptor-RlucII and 1000–1500 ng of β-arrestin-mKO). For TRUPATH assays^65^, 1000 ng Gα-Rluc8, 1000 ng Gβ3, and 1000 ng Gγ9-GFP2 were transfected. For receptors, 1000 ng ACKR3, 1000 ng MOR, 500 ng V2R, and 500 ng of CXCR3 were transfected. For AGS3 NanoBRET assays, 50 ng of Gα-Nanoluc, 500 ng β-arrestin-mKO, 2000 ng of untagged receptor, and 2000 ng AGS3 were used. For NanoBiT BRET, 100 ng of Gαi-LgBiT, 500 ng of Gβ1, 500 ng Gγ2, 500 ng, SmBiT-βarrestin2.

After incubating overnight at 37 °C, cells were seeded into Costar 96-well plates at 50,000–100,000cells/well in starvation media (see NanoBiT Assay for details). After overnight incubation, starvation medium was removed and replaced with 80 μL of assay buffer containing 20 mM HEPES and 3 μM of coelenterazine-h for BRET1 assays diluted into HBSS without calcium or magnesium. For TRUPATH, 3 μM coelenterazine-400a (NanoLight Technologies) was used in experiments with ACKR3 & MOR and 3 μM prolume purple (NanoLight Technologies) for V2R and CXCR3. After 5 min, cells were stimulated with appropriate ligands and treatments and analyzed via the following plate reader protocols:

For receptor internalization BRET, β-arrestin recruitment BRET, and NanoBRET assays, plates were read using the BioTek Synergy-Neo2 plate reader set to 37 °C with either the standard Rluc 480 nm emissions filter and a mKO 542 nm long-pass emission filter (Agilent). After stimulation, plates were read 30 times with a 0.5 s integration time. Raw BRET ratio was calculated via dividing the mKO signal by the luminescence signal. ΔNet BRET was calculated by subtracting the average Raw BRET signal of the vehicle wells from the Raw BRET signal of the stimulated wells.

For TRUPATH, plates were read using the BioTek Synergy-Neo2 plate reader or a Glomax reader (Promega) set to 37 °C and a Rluc8 400 nm emissions filter and GFP 510 nm emissions filter. After stimulation, plates were read 4–6 times. Raw BRET ratio was calculated via dividing the GFP2 signal by the luminescence signal. Δ Net BRET was calculated by subtracting the average Raw BRET signal of the vehicle wells from the Raw BRET signal of the stimulated wells.

For NanoBiT BRET assays, plates were read on a Berthold Mithras LB940 warmed to 37 °C with standard Rluc 485 nm emission filter and a custom mKO 542 nm long-pass emission filter (Chroma Technology Co. Bellows Falls, VT). Plates were read three times to gather basal luminescence and then stimulated with the appropriate concentration of ligand. Afterwards, cells were read for an additional 13 reads to monitor response. ΔNet BRET was calculated by subtracting the average Raw BRET signal of the vehicle wells from the Raw BRET signal of the stimulated wells.

### AlF_4_ assays

Cells were transfected via calcium phosphate and plated into 96-well plates as previously denoted in the Gαi:β-arrestin NanoBRET methods utilizing V2R as the untagged receptor of choice. On the day of assay reading, starvation media was replaced with 60 μL of assay buffer containing 20 mM HEPES and 3 μM of coelenterazine-h diluted into HBSS without calcium or magnesium. Three basal reads were taken prior to stimulation on the BioTek Synergy-Neo2 plate reader at 37 °C using Rluc 480 nm emissions filter and a YFP 530 nm long-pass emission filter (Agilent). Cells were then treated with 20 µL solution containing 10, 30, or 60 µM of AlF_3_ with 10 mM NaF to generate 10, 30, or 60 µM of AlF_4_ respectively. Cells were read 30 times with the NanoBRET reading protocol described above to monitor change in BRET. Additionally some non-AlF_4_ treated wells were stimulated with 500 nM AVP as a positive control. Following the initial 30 reads, another three basal reads were taken and then 20 µL of AVP at various concentrations was added to AlF_4_ treated wells to monitor dose response between 1 pM and 10 µM AVP final. After stimulation, the plate was read for an additional 30 reads and a dose response was calculated at max signal. AlF_3_ and NaF were purchased from Sigma-Aldrich (St. Louis, MO).

### Recombinant β-arrestin-1 and β-arrestin-2 expression and purification

N-terminally Protein-C tagged rat β-arrestin-1 393X lacking cysteine residues or N-terminally Protein-C tagged bovine β-arrestin-2 392X was purified from BL21 DE3 *E. coli*, with species selection chosen based on previously determined bacterial expression levels^66^. A pMCSG9 expression plasmid encoding maltose binding protein preceded by a hexahistidine tag and followed by a tobacco etch virus cleavage site was appended to the N-terminus of both β-arrestin expression vectors. 10 mL starter cultures were grown in TB supplemented 4% (v/v) glycerol and 100 μg/mL ampicillin to an OD_600_ of ~2 at 37 °C. Bacteria were cooled to 20 °C for ~2 h, induced with 200 μM IPTG, and grown overnight at 20 °C. Pellets were harvested and stored at -80 °C until purification. For purification, bacterial pellets were lysed in 50 mM MOPS pH 7.5, 300 mM NaCl, 2 mM DTT lysis buffer with the addition of protease inhibitor tablets and 0.5 mL of 3 mg/mL lysozyme added per liter of culture. After stirring for ~30 min at 4 °C, lysates were subsequently passed through a LM10 Microfluidizer Processor three times. Insoluble components were removed via centrifugation (50,000 *g* for 30 min), and supernatant was syringe filtered through an 0.22 μm filter and passed over Nickel-NTA resin by gravity flow. The column was then washed with 20 column volumes of lysis buffer and eluted with lysis buffer with the addition of 200 mM imidazole. Elution was dialyzed overnight in 50 mM MOPS pH 7.5, 300 mM NaCl, 2 mM DTT with the addition of TEV protease. In the morning, the dialyzed sample was passed over a nickel gravity column twice to remove the cleaved MBP, and then diluted with 50 mM MOPS pH 7.5, 2 mM DTT to a final salt concentration of 100 mM NaCl. Here, purification strategies for β-arrestin-1 and β-arrestin-2 diverged based on a modified protocol from the Gurevich laboratory^66^. β-arrestin-1 was further purified by ion exchange via loading over a HiTrap QXL Column (Cytiva), washed with MOPS pH 7.5, 100 mM NaCl, 100 μM TCEP and eluted with a gradient elution over 40 CV with 50 mM MOPS pH 7.5, 1 M NaCl, and 100 μM TCEP (estimated NaCl concentration at elution peak of 110 mM). β-arrestin-2 was further purified by loading first over a HiTrap QXL column (Cytiva) linked in tandem with a HiTrap SP FF column (Cytiva). After loading, the Q column containing impurities was removed. The SP FF column was then washed with MOPS pH 7.5, 100 mM NaCl, 2 mM DTT and then eluted with a gradient elution over 40 CV with 50 mM MOPS pH 7.5, 1 M NaCl, and 2 mm DTT (estimated NaCl concentration at elution peak of 300 mM). Protein purity was confirmed by SDS–PAGE. Protein was further concentrated, and 10% v/v glycerol was added to the sample. Protein was flash frozen in liquid nitrogen and stored at −80 °C until use.

### Recombinant non-lipidated Gαi expression and purification

Human Gαi1 lacking the first three amino acids to remove the lipidation residue were cloned into a modified pET51b expression vector. Gαi1 was preceded by a 10x histidine tag and followed by a 3 C cleavage site. 10 mL starter cultures were grown in TB supplemented 4% (v/v) glycerol and 50 μg/mL kanamycin to an OD_600_ of ~2 at 37 °C. Bacteria were cooled to 20 °C for ~2 h, induced with 200 μM IPTG, and grown overnight at 20 °C. Pellets were harvested and stored at −80 °C until purification. For purification, cells were resuspended in lysis buffer (20 mM HEPES pH 7.4, 100 mM NaCl, 10 mM MgCl₂, 5 mM imidazole, 5 mM β-mercaptoethanol, 20 μM GDP) at a ratio of 3 mL per gram of cell pellet. Protease inhibitor tablets (1 per 150 mL) and benzonase (1 μL per 150 mL) were added. Cells were lysed using an LM10 homogenizer following a 20–30 min preincubation on ice. Lysates were clarified by centrifugation at 50,000 × *g* for 30 min at 4 °C and filtered through glass fiber filters. The clarified lysate was applied to 2 mL of Ni-NTA resin pre-equilibrated with lysis buffer. The column was washed with 10 CVs of Wash Buffer 1 (20 mM HEPES pH 7.4, 250 mM NaCl, 10 mM imidazole, 5 mM βME, 2 mM MgCl₂, 10 μM GDP), followed by 10 CV of Wash Buffer 2 (same as Wash Buffer 1 with 20 mM imidazole). Protein was eluted using 4–8 CV of Elution Buffer (20 mM HEPES pH 7.4, 100 mM NaCl, 300 mM imidazole, 5 mM βME, 2 mM MgCl₂, 10 μM GDP). Elution was monitored using Bradford reagent. The eluate was concentrated to ~12 mL using a 30 kDa cutoff centrifugal filter and incubated with 3 C protease at a 1:50 (w/w) estimated protein ratio. The sample was dialyzed overnight at 4 °C in 2 L of dialysis buffer (20 mM HEPES pH 7.4, 100 mM NaCl, 5 mM βME, 1 mM MgCl₂, 10 μM GDP).

Following 3 C protease cleavage, the sample was flowed over a second Ni-NTA column to remove uncleaved protein and 3 C protease. The flow-through, containing cleaved Gα protein, was collected. The column was further washed with dialysis buffer and subsequently eluted to recover any residual bound proteins. Samples were analyzed by SDS-PAGE. Pooled fractions containing Gαi1 were treated with a phosphatase mix for 1 h on ice: 5 μL λ phosphatase, 2 μL Quick CIP, 1 μL Antarctic phosphatase, and 1 mM MnCl₂. The sample was diluted 1:1 in Ion Exchange Dilution Buffer (20 mM HEPES pH 7.4, 5 mM βME, 1 mM MgCl₂, 10 μM GDP) to a final NaCl concentration of 50 mM.

Ion exchange chromatography was performed using a Mono Q 10/100 column (Cytiva). The sample was loaded via sample pump, washed with 5 CV of Buffer A (20 mM HEPES pH 7.4, 50 mM NaCl, 1 mM TCEP, 1 mM MgCl₂, 10 μM GDP), and eluted with a linear gradient from 0 to 40% Buffer B (same as A with 1 M NaCl) over 40 CV. Relevant fractions were pooled, analyzed by SDS-PAGE, concentrated, and stored with 10% glycerol at −80 °C.

### Recombinant non-lipidated γ C68S expression and purification

Sf9 insect cells expressing N-terminally His-tagged Gβγ C68S were resuspended in lysis buffer (20 mM HEPES pH 7.4, 100 mM NaCl, 2 mM MgCl₂, 5 mM imidazole, 5 mM β-mercaptoethanol (βME)) supplemented with protease inhibitors and Benzonase. Following homogenization and centrifugation at 50,000 × *g* for 30 min, supernatant was filtered and applied to Ni-NTA resin (Qiagen) pre-equilibrated in lysis buffer. Resin was washed sequentially with Wash 1 (20 mM HEPES pH 7.4, 250 mM NaCl, 10 mM imidazole) and Wash 2 (20 mM HEPES pH 7.4, 250 mM NaCl, 30 mM imidazole) buffers, and bound protein was eluted with HEPES pH 7.4100 mM NaCl, 300 mM imidazole, 5 mM βME. Eluate was incubated overnight with His-tagged 3 C protease (~1:50, w/w) during dialysis 20 mM HEPES pH 7.4, 100 mM NaCl, 5 mM βME. Post-cleavage, samples were passed twice over Ni-NTA resin; the flow-through contained Gβγ, while His-tagged contaminants and protease remained bound. Flow-through was pooled and treated for 1 h on ice with λ-phosphatase, Quick CIP, and Antarctic phosphatase in the presence of 1 mM MnCl₂. The sample was diluted to 50 mM NaCl in 20 mM HEPES pH 7.4, 5 mM βME, and further purified by anion exchange chromatography on a Mono Q 10/100 column (Cytiva). Bound protein was eluted with a linear gradient from 0 to 40% Buffer B (20 mM HEPES pH 7.4, 1 M NaCl, 1 mM TCEP) over 40 column volumes. Fractions containing were verified by Coomassie gel. Gβγ fractions were pooled, concentrated, supplemented with 20% glycerol, aliquoted, snap-frozen in liquid nitrogen, and stored at −80 °C until use.

### Pull downs

Fresh anti-Protein C resin was prepared as a 1:1 slurry in buffer (20 mM HEPES pH 7.4, 100 mM NaCl, 1 mM MgCl₂, 100 μM TCEP, 2 mM CaCl₂). For each condition, 25 μL of resin (equivalent to 50 μL slurry) was transferred to a 1.5 mL microcentrifuge tube, spun at 3500 × *g* for 2 min at 4 °C, and washed with 1 mL of Buffer. Tubes were shaken gently for 10 min in the cold room, followed by a second wash and 5-minute shake.

Protein complexes containing Protein C (Prc)-β-arrestin-1-393X and non-lipidated Gαi1 were assembled at a final concentration of 25 μM on ice for 45 min, then incubated with either no treatment, 20 μM GDP, 20 μM GTPγS, or 0.25 units of apyrase (New England Biolabs) for 45 min, then equilibrated with anti-Protein C resin for 30 min in the cold room gently shaking. Resin was pelleted at 3500 × *g* for 2 min at 4 °C and the supernatant was collected. Beads were washed once with buffer, spun again under the same conditions, and residual buffer was carefully removed using a narrow-31-gauge needle. Proteins were eluted in 40 μL of Elution Buffer (20 mM HEPES pH 7.5, 100 mM NaCl, 1 mM MgCl₂, 100 μM TCEP, 5 mM EDTA, 0.2 mg/mL Protein C peptide) by shaking for 20 min in the cold room, followed by centrifugation at 3500 × *g* for 3 min at 4 °C. The supernatant was collected using a narrow 31-gauge needle. Eluates were mixed with SDS loading buffer and resolved by SDS-PAGE on 10-well gels at 200 V for 35 min. Gels were stained with 1x One-Step Blue Protein Gel Stain (Biotium) and imaged on a Bio-Rad Gel Doc XR+ under Coomassie gel image protocol.

For Prc-β-arrestin-1-393X pull downs with non-lipidated Gαi and non-lipidated G, experiments were conducted similar to the above, aside from using Protein G coated magnetic bead resin coupled to anti-Protein C antibodies. For each condition, 25 μL of resin (equivalent to 50 μL slurry) was equilibrated in buffer 20 mM HEPES pH 7.4, 100 mM NaCl, 1 mM MgCl₂, 100 μM TCEP, 2 mM CaCl₂ + 10 μM GDP. Protein complexes containing Prc-β-arrestin-1-393X, non-lipidated Gαi1, and non-lipidated Gβγ were assembled at a final concentration of 25 μM on ice for 60 min, then equilibrated with anti Protein C magnetic bead resin for 30 min at at 4 °C gently shaking. Resin was pelleted via MagJET separation rack and the supernatant was removed. Beads were washed four times with 500 μL of buffer, separated from the magnetic bead resin through the same mechanism, with residual buffer carefully removed without disturbing the magnetized bead pellet. Protein was eluted and resolved by SDS-PAGE as described above. For quantification of Gαi pull down efficiency, pixel intensity was quantified in Image Lab 6.1. Non-specific binding of Gαi to anti-protein C resin was subtracted, and percent Gαi intensity was normalized to amount of Prc-β-arrestin-1-393X pulled down.

### [^35^S]-GTPγS binding assays

Gα GTP*γ*S binding assays were described previously^67,68^. Briefly, Gαi_1_ or myristoylated-Gαi_2_ (100 nM) were incubated with or without a mixture of β-arrestin-1-393X and β-arrestin-2-392X (400 nM each) at 25 °C in GTP*γ*S binding buffer (20 mM HEPES, pH 8, 100 mM NaCl, 2 mM DTT, 1 mM EDTA, 10 mM MgCl_2_ and 0.05% (m/v) C12E10 and 10 µM [^35^S]GTP*γ*S (SA 4000 cpm/pmol). Aliquots were taken from biological triplicate reactions at indicated time points (3, 6, 10, 15, 30, 60, 90 min), quenched in GTP quench buffer (20 mM Tris, pH 7.7, 100 mM NaCl, 10 mM MgCl_2_, 1 mM GTP, and 0.08% (m/v) C12E10), and filtered onto BA-85 nitrocellulose filters (GE Healthcare Life Science). Filters were washed (20 mM Tris, pH 7.7, 100 mM NaCl, 2 mM MgCl_2_), dried and subjected to scintillation counting.

### Ligands

AVP, dopamine, AngII, DAMGO, BAM22, Quinpirole, Fentanyl, isoproterenol, tiotropium, and VUF10661 were purchased from Sigma-Aldrich (St. Louis, MO). PZM21, SR17018, and TRV130 (Oliceridine) were purchased from MedKoo Biosciences (Cary, NC). TRV120023 was synthesized by Genscript (Piscataway, NJ). WW36, WW38, were provided via a collaboration with RTI International (Research Triangle Park, NC). Stock solutions of AVP, AngII, DAMGO, BAM22, and VUF10661 were prepared according to the manufacturer’s specifications. Isoproterenol, WW36, and WW38 were dissolved in dimethyl sulfoxide (DMSO) and stored in a desiccator cabinet. Fentanyl was provided pre-solubilized in ethanol vials (1 mg/mL). Pre-solubilized Rapalog (AP21967) vials were purchased from Takara Bio (Mountain view, CA). All drug dilutions were performed with NanoBiT, BRET, or cell culture medium. PTX was obtained from List Biological Laboratories (Campbell, CA) and stored at 4 °C. All compound stocks were stored at −20 °C until use.

### Sequence alignments

Human protein sequence alignments for Gαi1, Gαi2, and Gαi3 were obtained from GproteinDb^69,70^ and visualized on SnapGene (San Diego, CA).

### Data analysis

Data were analyzed via Excel (Microsoft, Redmond, WA) and graphed in Prism 10.0 (GraphPad, San Diego, CA). Log agonist versus stimulus with three parameters (span, baseline, and EC50) was used to determine dose-response curves for applicable data sets. Comparisons of ligand or mutant constructs in concentration and time response assays were conducted via a two-way analysis of variance (ANOVA) unless otherwise noted. Most experiments were conducted three or more times. Some replicates represented positive or negative controls. Statistical tests were two-sided and Bonferroni analysis was corrected for multiple comparisons unless otherwise noted. Lines represent mean values and error bars represent SEM.

### Reporting summary

Further information on research design is available in the Nature Portfolio Reporting Summary linked to this article.

## Supplementary information

Supplementary Information

Reporting Summary

Transparent Peer Review file

## Source data

Source Data

## Data Availability

The authors declare that all data supporting the findings are available within the paper and the Supplemental Data. Source data are provided with this paper.
