# Supplementary material for: β-arrestin recruitment facilitates a direct association with G proteins
Source: Nat Commun. 2026 Jul 17;17:8840. doi: 10.1038/s41467-026-74944-7 (PMC13500827; doi:10.1038/s41467-026-74944-7)
Supplement: Supplementary file 1 [file 41467_2026_74944_MOESM1_ESM.pdf]

**SUPPLEMENTARY INFORMATION**

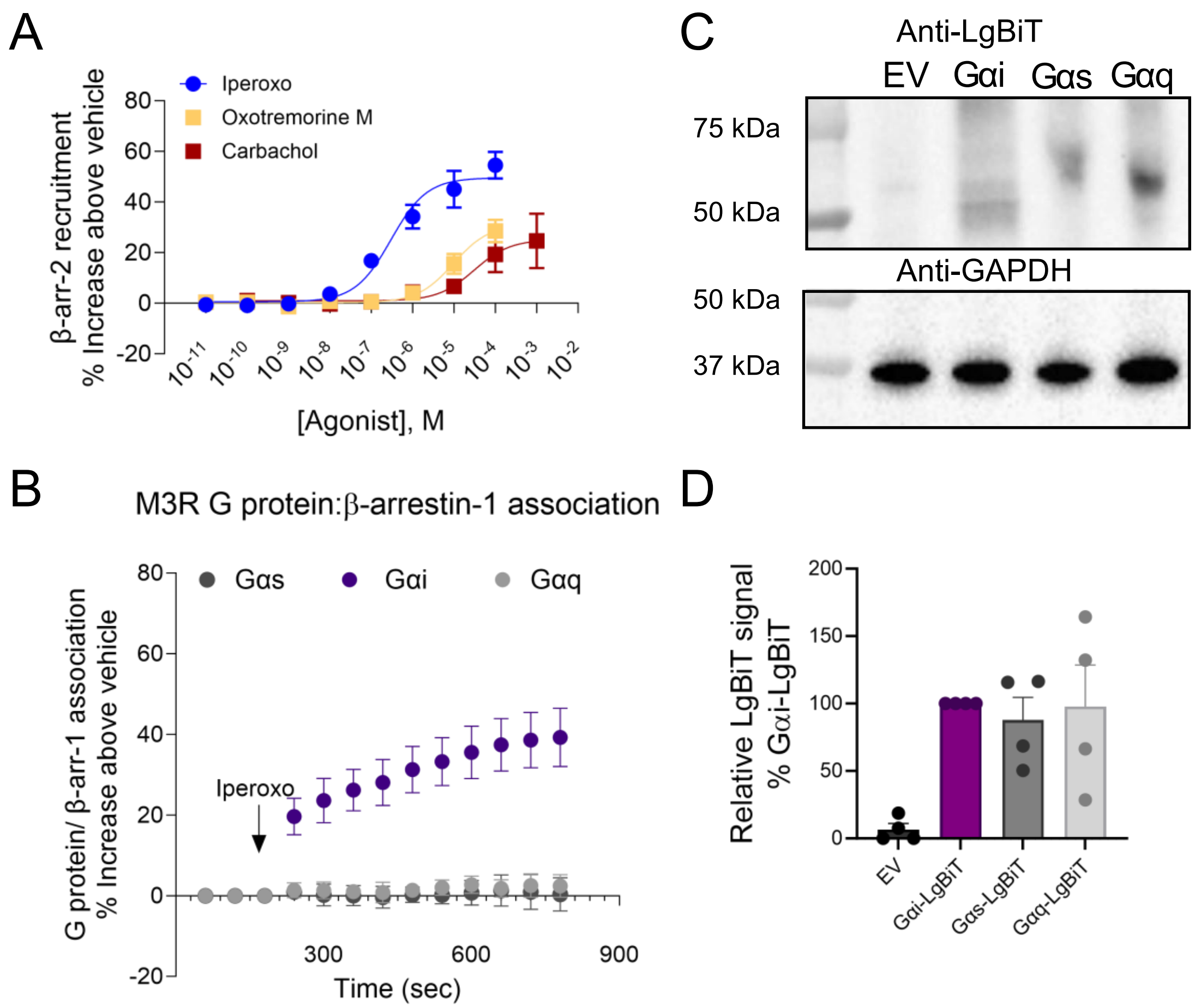


**Supplementary Figure 1: M3R-induced β-arrestin recruitment and Gα-LgBiT expression.**

(A) Nluc-β-arrestin-2 recruitment to the plasma membrane (myrpalm-mVenus) in HEK293T cells overexpressing the M3R following treatment with the indicated agonist as assessed by BRET (n=4 for Oxotremorine and iperoxo, n=2 for carbachol treatment conditions). (B) HEK293T cells overexpressing the M3R and smBiT-β-arrestin-1 association with the indicated Gα-LgBiT construct after treatment with iperoxo (1 μM). (C) Representative western blot of HEK293T cells overexpressing the indicated Gα-LgBiT construct anti-LgBiT construct, representative of n=3 experiments. (D) Pixel intensity of anti-LgBit signal adjusted for loading and normalized to Gαi-LgBiT, n=3 per condition. EV, empty vector. Graphs depict the mean values ± SEM.

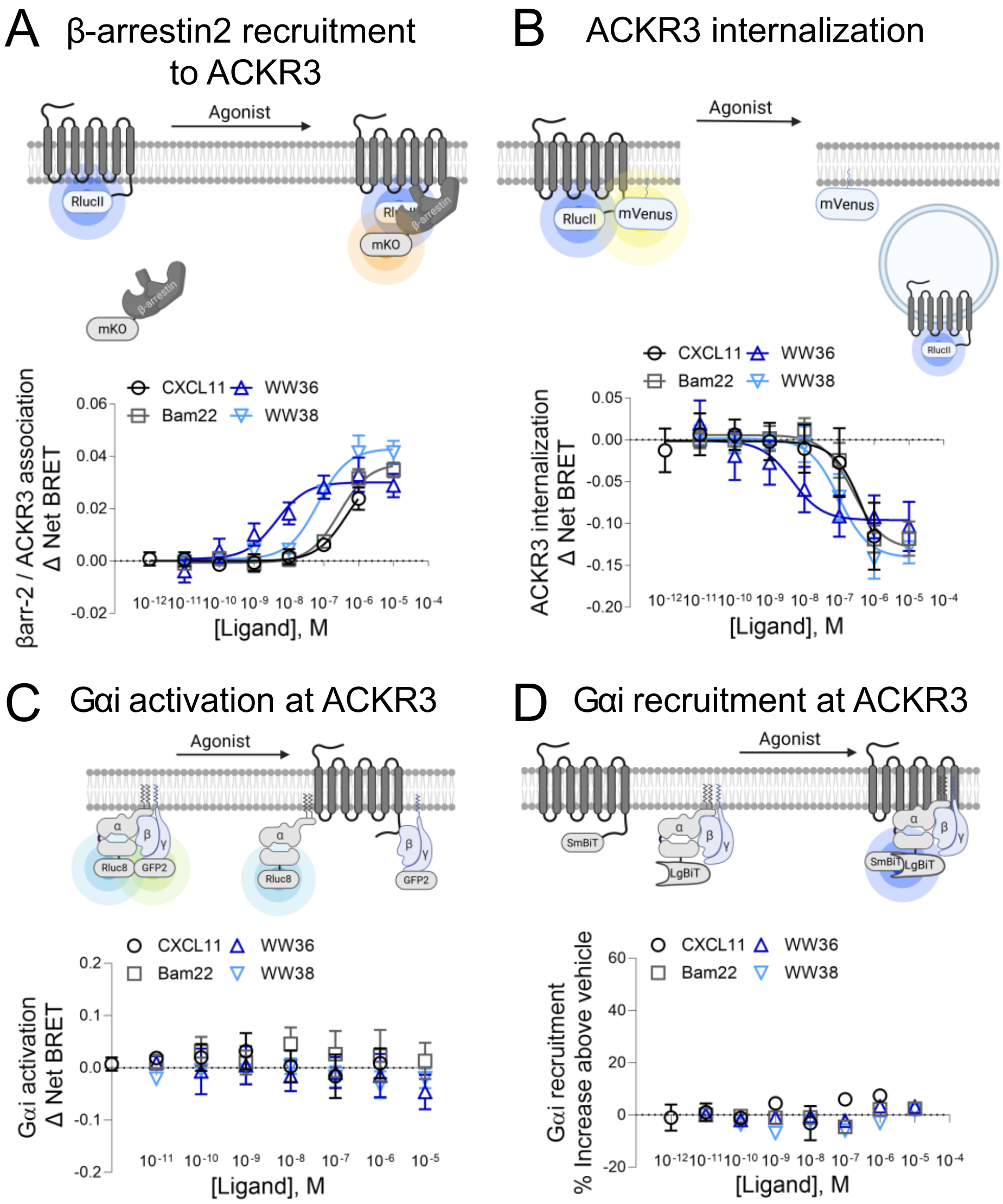


**Supplementary Figure 2: No activation or recruitment of Gαi at ACKR3 despite its ability to form Gαi:β-arrestin complexes.**

Canonical signaling in HEK293T cells overexpressing ACKR3 following treatment with CXCL11, Bam22, WW36, and WW38 by the following assays: (A) β-arrestin2 recruitment to ACKR3 assessed by BRET between ACKR3 tagged with Renilla-luciferase II (RlucII) and β-arrestin-2-mKO. n=4 all ligands. Graphic created in BioRender. Lee, C. (2026) https://BioRender.com/bruk70t (B) ACKR3 Internalization assessed by loss of BRET between Myrpalm-mVenus and ACKR3-RlucII upon endocytosis.  n= 4 CXCL11, WW38; n=8 Bam22, WW36. Graphic created in BioRender. Lee, C. (2026) https://BioRender.com/i4q5fo1(C) G protein activation by ACKR3 assessed by TRUPATH BRET2 assay. n=3 for all ligands. Graphic created in BioRender. Lee, C. (2026) https://BioRender.com/s8qov0g (D) Gαi recruitment to

ACKR3 assessed by NanoBiT recruitment of Gαi-LgBiT to ACKR3-SmBiT. n=3 for all ligands except CXCL11 which is n=2. Graphic created in BioRender. Lee, C. (2026) https://BioRender.com/a287sr3. All replicates represent independently run plate assays. Graphs depict the mean values at each dosage of ligand ± SEM.

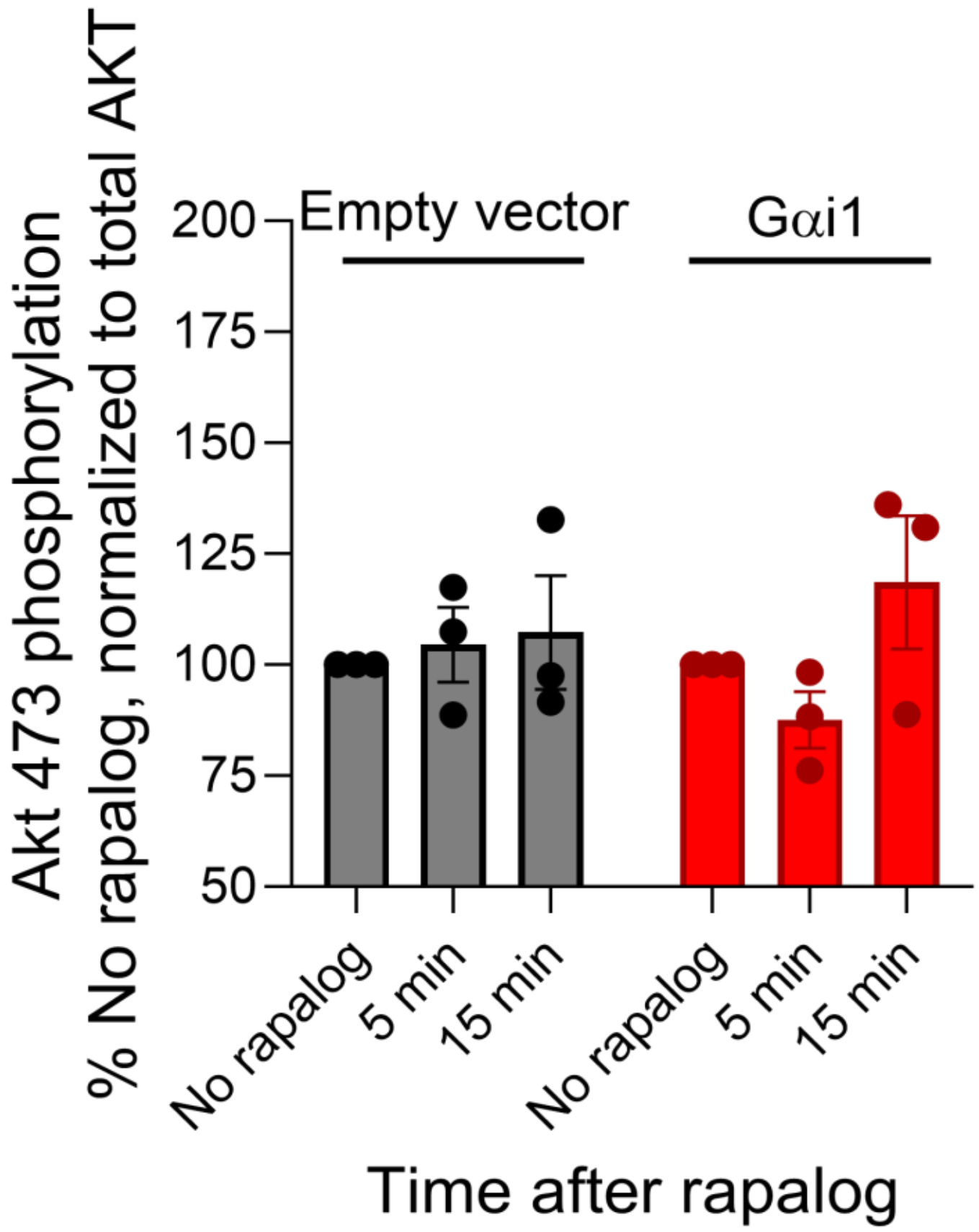


**Supplementary Figure 3: Akt Ser 473 phosphorylation upon β-arrestin translocation to the plasma membrane with Gαi rescue in G protein knockout cells.**

HEK293T cells lacking G proteins were transiently transfected with were transfected with Lyn-FRB (LDR), SmBiT-β-arrestin-2-FKBP, and empty vector or Gαi1 rescue. Cells were treated with rapalog for the indicated time to translocate β-arrestin-2 to the plasma membrane and analyzed for Akt Ser473 phosphorylation. Unlike Akt Thr 308, no significant change in Ser 473 phosphorylation was observed in either condition. Graphs depict the mean values of n=3 ± SEM.

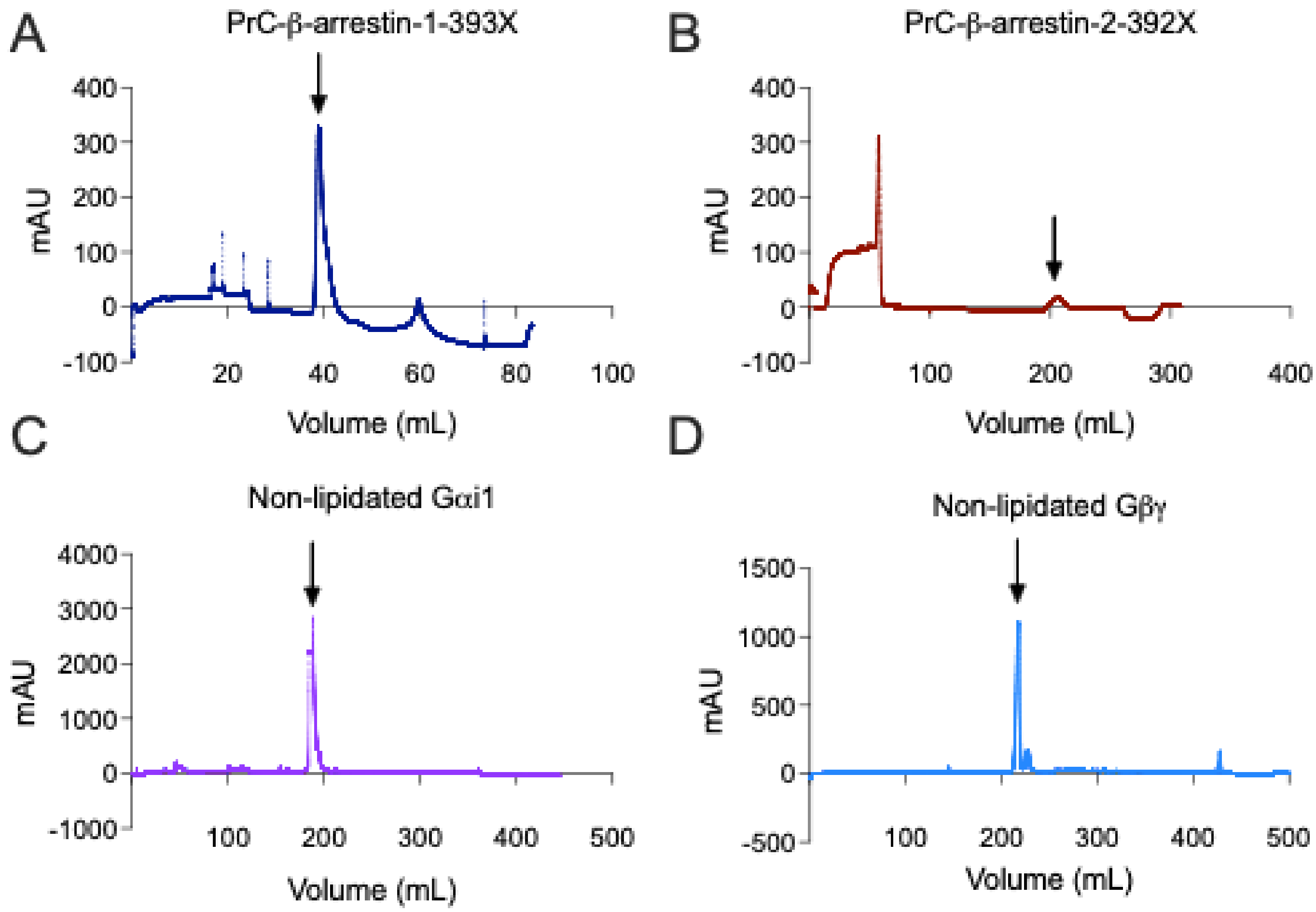


**Supplementary Figure 4: Size exclusion chromatography traces.**

Monodispersed peaks were observed for purification of (A) N-terminally tagged Protein C β-arrestin-1 393X, (B) N-terminally tagged Protein C β-arrestin-2 392X, (C) non-lipidated Gαi1, and (D) non-lipidated Gβγ. Purity of the fractions indicated with arrows was confirmed by Coomassie gel.

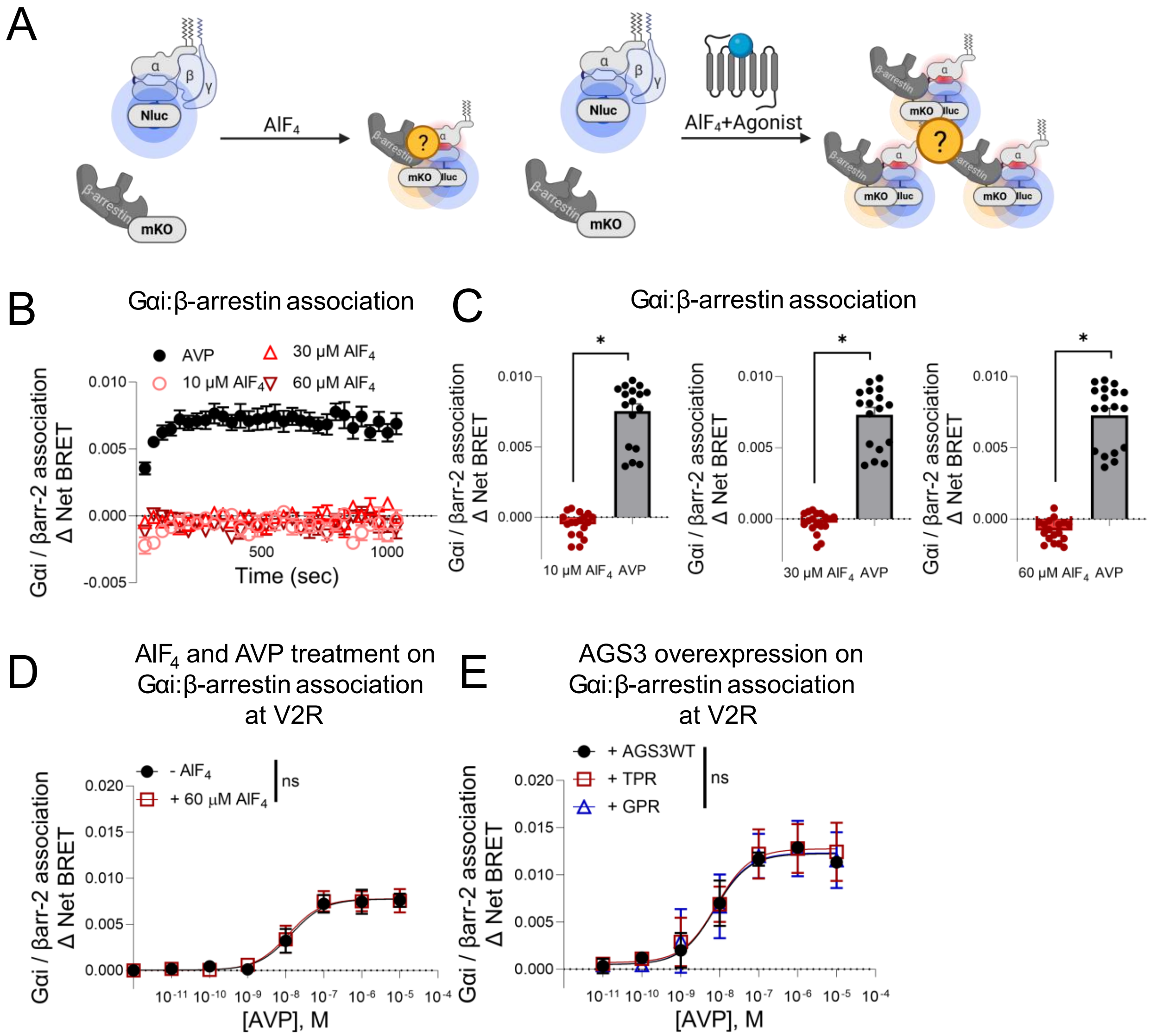


**Supplementary Figure 5. No increase of Gαi:β-arrestin complex formation after receptor independent modulation of Gαi.**

(A) Illustration of $AlF_4$ NanoBRET assay. Briefly, HEK293T cells were transiently transfected with V2R, Gαi-Nluc (Nanoluciferase), and β-arrestin-2-mKO. Cells were treated either vehicle or $AlF_4$ then immediately placed in a plate reader. Afterwards, AVP or vehicle was added to cells with $AlF_4$ treatment to activate V2R and test the effect of $AlF_4$ and AVP co-treatment in Gαi:β-arrestin-2 complex formation. Created in BioRender. Lee, C. (2026) https://BioRender.com/77vkggi (B) NanoBRET kinetics comparing Gαi:β-arrestin cells treated with varying concentrations of $AlF_4$ or AVP (500 nM) (C) Δ Net BRET ratio between reads 10-15 from the kinetic read in panel B displayed as a bar graph. *P<0.0001 by one-way ANOVA with Dunnett post hoc comparing $AlF_4$ treatment versus AVP. (D) NanoBRET dose response curve comparing the Δ Net BRET ratio following AVP treatment (500 nM) in cells that had no pre-treatment of $AlF_4$ or 60 μM of

$AlF_4$ treated prior. (E) NanoBRET assay comparing formation of Gαi:β-arrestin after V2R activation with AVP when AGS3, TPR, or GPR is overexpressed. ns; P>0.05 by two-way ANOVA with bonferroni post hoc test comparing $AlF_4$ treatment to non-treated groups (D) or cells overexpressed with AGS3WT to TPR or GPR mutant overexpression controls (E). n=3 independently run plate replicates. Graphs depict mean values ± SEM.

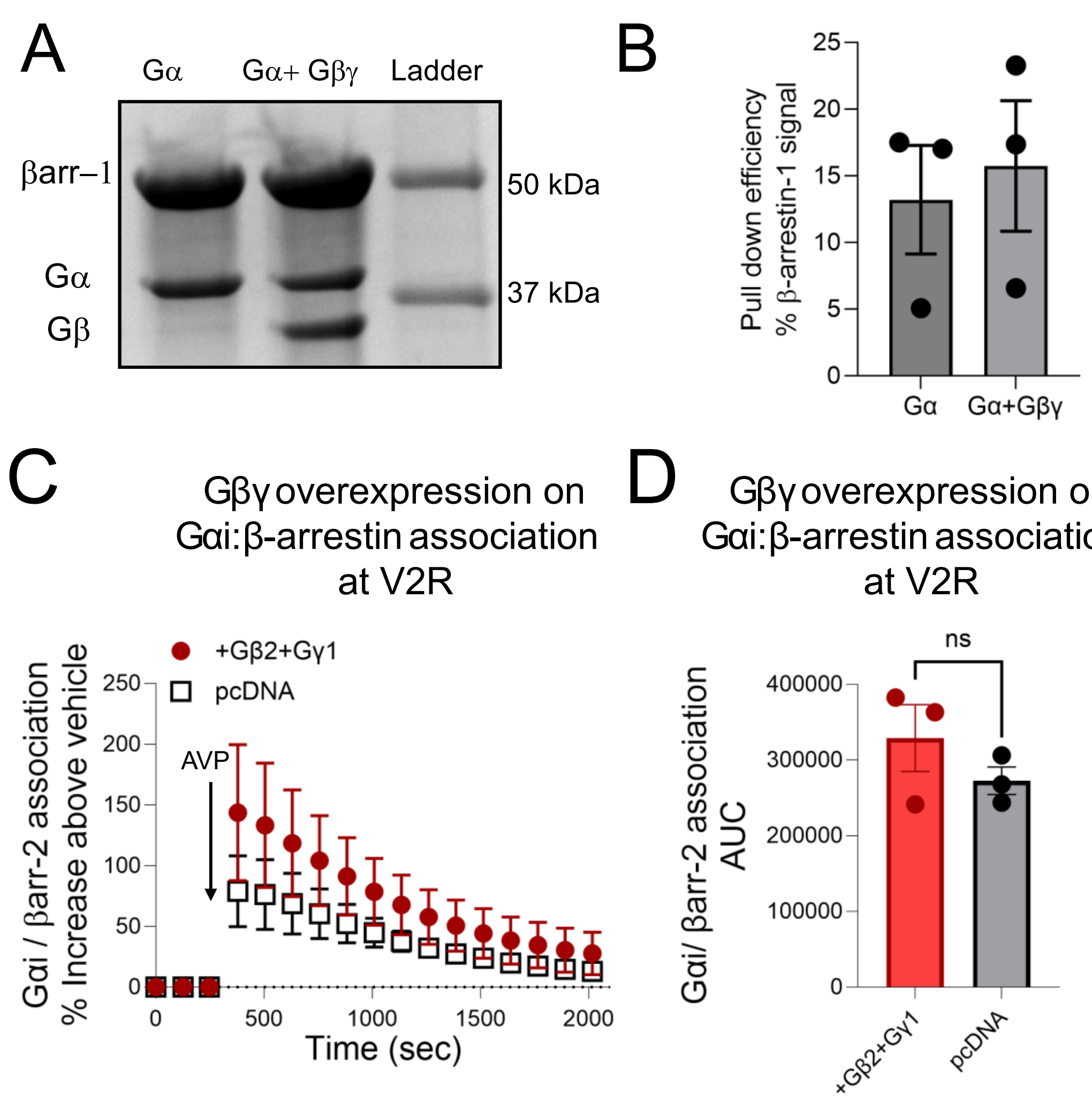


**Supplementary Figure 6. Gβγ is dispensable for Gαi:β-arrestin complexes.**

(A) Coomassie gel of pull down of β-arrestin purified non-lipidated Gαi1 with purified Gβ and Gγ. (B) Quantification of bands in panel A via densinometry. (C) Kinetics of Gαi-LgBiT:SmBiT-β-arrestin with Gβ1 and Gγ2 overexpression or a pcDNA control following V2R treatment with AVP (500 nM). (D) AUC quantification of the kinetic curve. ns P>0.05 by two-tailed t test comparing Gβ1 and Gγ2 overexpression versus pcDNA control. All data are n=3 and represent independent pull downs or plate replicates, respectively. Graphs in Panel C and D demonstrate net increase of AVP over vehicle treatment. Graphs depict mean values ± SEM.

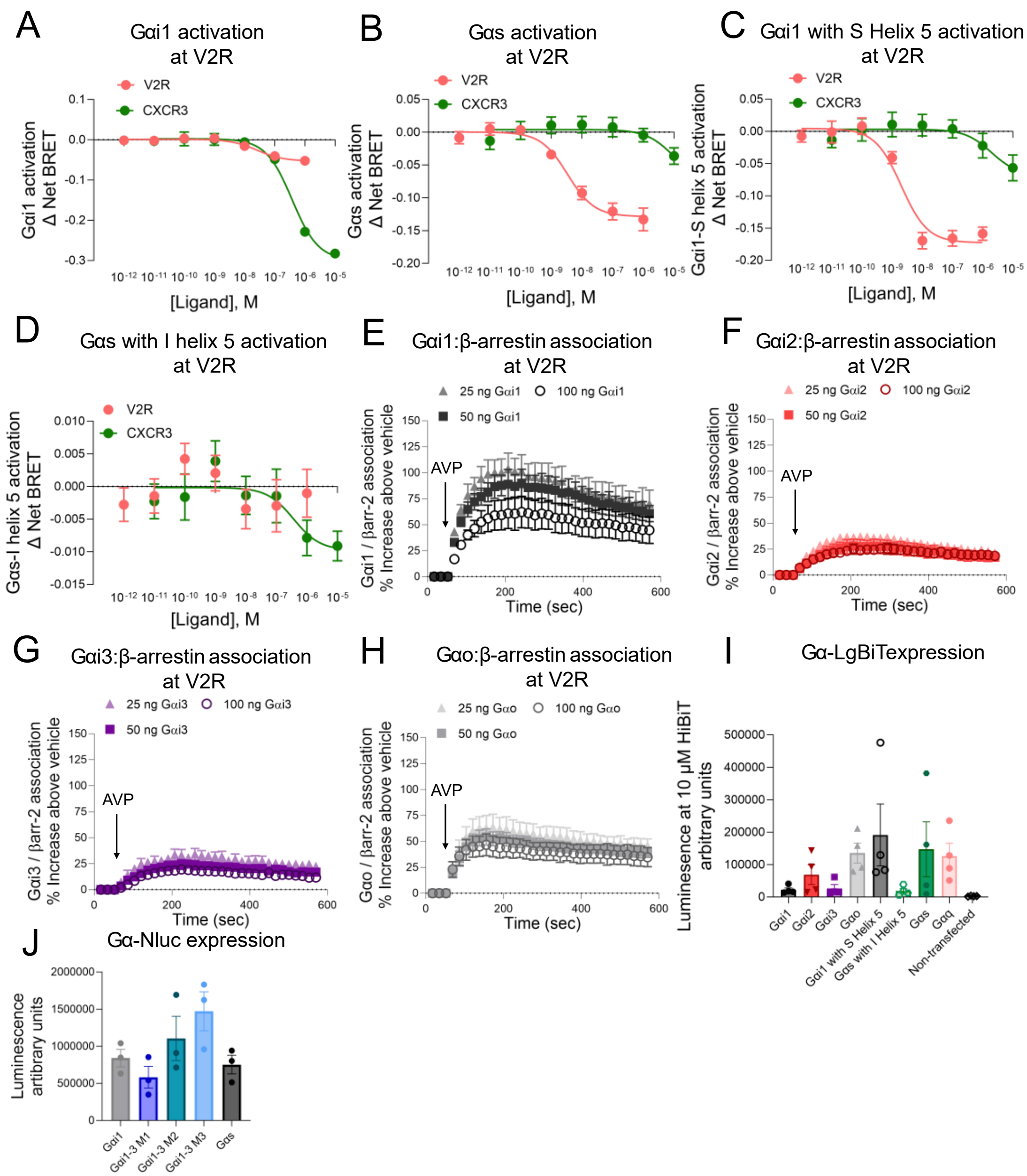

**Supplementary Figure 7. Gα-Helix 5 swaps retain capability to activate and Gαi2 and Gαi3 do not complex as efficiently with β-arrestin2.**

HEK293T cells overexpressing the indicated TRUPATH G protein plasmid (WT Gαi1, WT Gαs, or the indicated helix 5 chimera) and either V2R (classically Gs-coupled) or CXCR3 (classically Gi-coupled) were assessed for G protein activation via TRUPATH G protein dissociation BRET. (A) Gαi, (B) Gαs, (C) Gαi with S Helix 5 (D) Gαs with I Helix 5 transfected cells were treated with either AVP (V2R) or VUF10661 (CXCR3) at the indicated concentrations. (E-H) Titrated amounts of (E) LgBiT-Gαi1, (F) LgBiT-Gαi2, (G) LgBiT-Gαi3, or (H) LgBiT-Gαo. Gαi:β-arrestin complex formation were assessed with NanoBiT luciferase signal upon activation of V2R with AVP (500 nM). (I) Gα-LgBiT protein expression determined by cell lysis and titrating HiBiT peptide. Graph displays

luminescence at max HiBiT added (10 μM) (J) Luminescence expression data for Nanoluc constructs in Fig 6I. Data show n=3 for all graphs at all conditions with the exception of Gαi1 at 100 ng in panel E which had n=4 and LgBiT expression in panel I which had n=4 for all conditions. Panels show mean ± SEM.

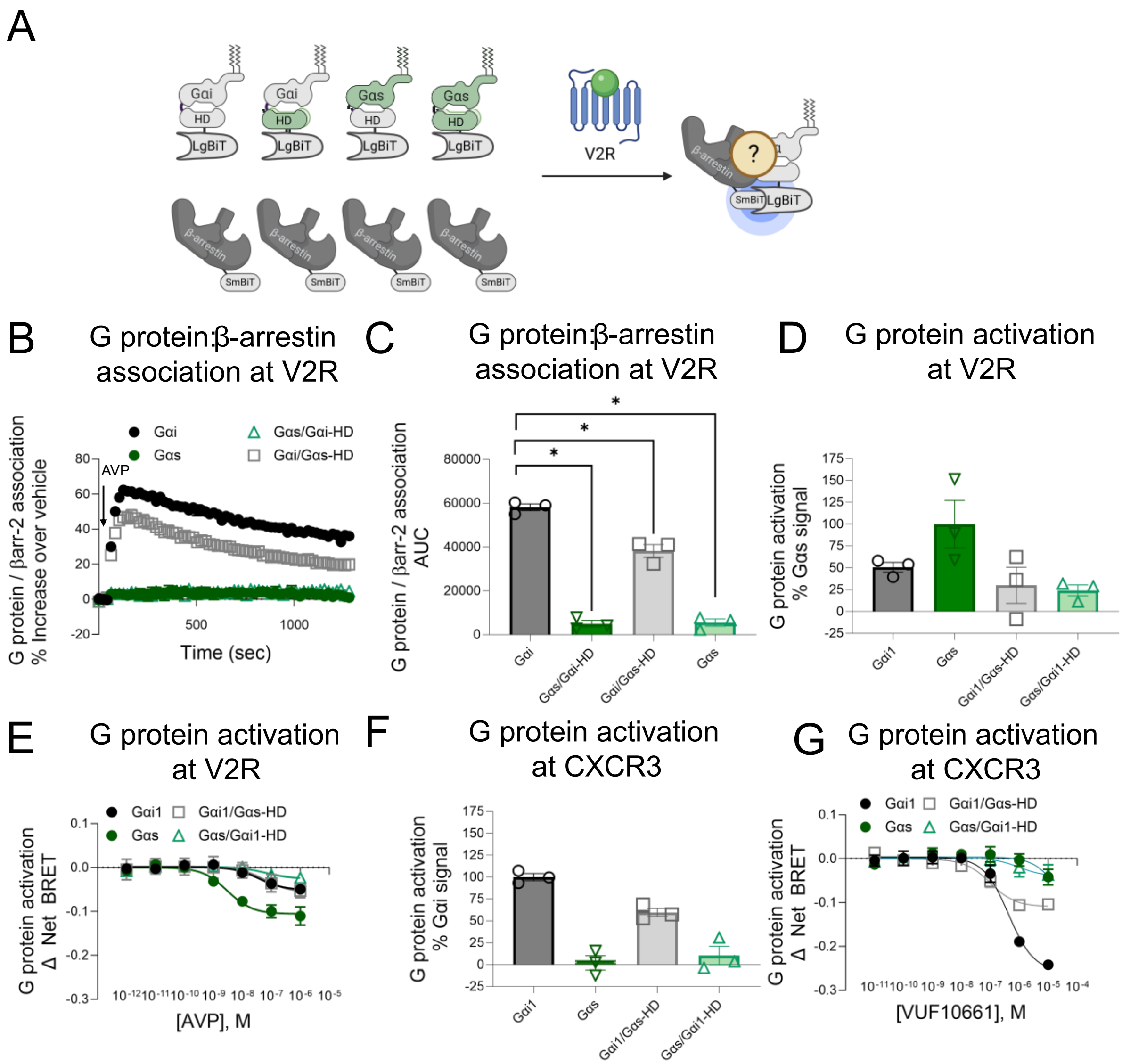


**Supplementary Figure 8. The Gαi1 alpha helical domain is not sufficient to promote G protein:β-arrestin association in HEK293T cells.**

(A) Schematic of helical domain swaps between Gαi1 and Gαs. Created in BioRender. Lee, C. (2026) https://BioRender.com/c7hefcj (B) Gα:β-arrestin-2 association in HEK293T cells overexpressing the indicated G protein chimera-LgBiT, SmBiT-β-arrestin-2, and V2R upon treatment with AVP (500 nM). (C) Area under the curve (AUC) analysis of the kinetics in panel B. *P=0.0003 by one-way ANOVA with Dunnett post hoc test comparing Gαi versus Gαi/GαsHD and *P<0.0001 Gαs/GαiHD, and Gαs versus Gαi/GαsHD and Gαs/GαiHD. (D) G protein activation at V2R shown as precent Gαs

signal at 1 μM AVP (E) Full dose response G protein activation at V2R from Panel D. (E) G protein activation at CXCR3 shown as a percent Gαi signal at 10 μM VUF10661. (G) Full dose response of G protein activation at CXCR3. All panels represent n=3 replicates and depict mean values ± SEM.

**Supplementary Table 1: List of Constructs Used**

| Construct Name | Characteristics | Source |
|---|---|---|
| **V2R** | Human Vasopressin 2 receptor | Ref (1) |
| **β2AR** | Human β2 Adrenergic Receptor | Ref (1) |
| **D2R-ARB** | Dopamine D2 Receptor β-biased mutant | Ref (2) |
| **AT1R** | Human Angiotensin II Type I Receptor | Ref (1) |
| **ACKR3** | Human Atypical Chemokine Receptor 3 | Rajagopal Lab |
| **M3R** | Human Muscarinic 3 acetyl choline receptor | Kruse Lab |
| **CXCR3** | Human C-X-C motif Chemokine Receptor 3 | Rajagopal Lab |
| **V2R-LgBiT** | Human Vasopressin 2 receptor with C-terminal LgBiT Tag | Rajagopal Lab |
| **ACKR3-RlucII** | Human atypical Chemokine Receptor 3 with C-terminal RlucII Tag | Rajagopal Lab |
| **ACKR3-SmBiT** | Human atypical Chemokine Receptor 3 with C-terminal SmBiT Tag | This work |
| **SmBiT-βarr2** | Mouse β-arrestin2 with N-terminal SmBiT Tag | Rajagopal Lab |
| **Gαs-LgBiT** | Human stimulatory G protein with LgBiT tag | Ref (1) |
| **Gαi1-LgBiT** | Human inhibitory G protein 1 with LgBiT tag | Ref (1) |
| **Gαi2-LgBiT** | Human inhibitory G protein 2 with LgBiT tag | Ref (1) |
| **Gαi3-LgBiT** | Human inhibitory G protein 3 with LgBiT tag | Ref (1) |
| **Gαo-LgBiT** | Human other G protein o with LgBiT tag | Ref (1) |

| | | |
|---|---|---|
| **βarr2-mKO** | Mouse β-arrestin2 with C-terminal mKO Tag | Rajagopal Lab |
| **Gβ1** | Human Gβ1 | Ref (1) |
| **Gγ2** | Human Gγ2 | Ref (1) |
| **Gαi1** | Human Gαi1 | Kruse Lab |
| **Gαi1-NanoLuc** | Human Gαi1 with Nanoluciferase tag | This work |
| **Gαi1-3 M1 LgBiT** | Human Gαi1 to Gαi3 mutant (A59D, S98A) with LgBiT tag | This work |
| **Gαi1-3 M2 LgBiT** | Human Gαi1 to Gαi3 mutant (A113S,F118V,A121P,P165S) with LgBiT tag | This work |
| **Gαi1-3 M3 LgBiT** | Human Gαi1 to Gαi3 mutant (K132R, S134G) with LgBiT tag | This work |
| **Gαi/GαsHD** | Human Gαi1 with Gαs helical domain tagged with LgBiT tag | This work |
| **Gαs/GαiHD** | Human Gαs to Gαi1 helical domain tagged with LgBiT tag | This work |
| **GNAI with S Helix 5 LgBiT** | Human Gαi1 with Gαs C-terminal α5helix tagged with LgBiT tag | This work |
| **GNAS with I Helix 5 LgBiT** | Human Gαs to Gαi1 C-terminal α5helix tagged with LgBiT tag | This work |
| **GNAI with S Helix 5 Rluc 8** | Human Gαi1 with Gαs C-terminal α5helix tagged with Rluc8 tag | This work |
| **GNAS with I Helix 5 Rluc 8** | Human Gαs to Gαi1 C-terminal α5helix tagged with Rluc8 tag | This work |
| **Non-lipidated Gai** | Human Gαi1 for bacterial expression | This work |
| **Non-lipidated Gbg** | Human Gbg (C68S) for insect cell virus production | This work |
| **AGS3** | human Activator of G protein Signaling 3 | Ref (3) |
| **TPR** | Human Activator of G protein Signaling 3 truncation mutant containing only the tetratricopeptide repeat (TPR) domain | Ref (3) |
| **GPR** | Human Activator of G protein Signaling 3 truncation mutant containing only the G protein regulatory domain (GPR) | Ref (3) |
| **Gαi1-Rluc8** | Human Gαi1 with Rluc8 Tag | Ref (4) |
| **Gαs-Rluc8** | Human Gαs with Rluc8 Tag | Ref (4) |
| **Gβ3** | Human Gβ3 used in TruPATH assay | Ref (4) |
| **Gγ9-GFP2** | Human Gγ9 with GFP2 Tag used in TruPATH assay | Ref (4) |

| | | |
|---|---|---|
| **MyrPalm-mVenus** | Myristolated and palmitoylated mVenus for plasma membrane localization | Ref (1) |
| **Lyn11-FRB** | Human FKBP-rapamycin-binding domain with N-terminal Lyn11 tag | Ref (5) |
| **Lyn11-FRB (LDR)** | Human FKBP-rapamycin-binding domain with N-terminal Lyn11 tag mutant for complex with rapalog | Ref (6) |
| **SmBiT-βarr2-FKBP** | mouse β-arrestin2 with N-terminal SmBiT Tag and C-terminal FKBP (FK506 binding protein) Tag | This work |
| **MOR** | Human Mu Opioid Receptor | cDNA.org (catalog number: #OPRM100000) |
| **CAAX-LgBiT** | CAAX Tagged LgBiT for plasma membrane localization | Rajagopal Lab |

**SupplementaryTable 2: List of Primers for General Constructs**

| Primer Name | Primer Sequence |
|---|---|
| **SmBiT-βarr2-FKBP FW** | 5' ACTGCAGCCAGGACCAGAGGACACAGGGAAGTGTTGTCCTGGTTGTTGTGCTTGTGGAGTAGACTTTGAGATTCGAGCTT 3' |
| **SmBiT-βarr2-FKBP RV** | 5' AAGCTCGAATCTCAAAGTCTACTCCACAAGCACAACAACCAGGACAACACTTCCCTGTGTCCTCTGGTCCTGGCTGCAGT 3' |
| **Gαi1 Nluc FW** | 5' ATATATGGATCCGTCTTCACACTCGAAGATTTCGTTGG 3' |
| **Gαi1 Nluc RV** | 5' ATATATGGATCCCGCCAGAATGCGTTCGC 3' |
| **ACKR3-SmBiT FW** | 5' ATATATGGTACCTTTGGTGCTCTGCTCCAAG 3' |
| **ACKR3-SmBiT RV** | 5' ATATATGCTAGCGCCACCATGGATCTGCATCTCTTCGACTA 3' |

**SupplementaryTable 3: List of Primers for G protein Mutations**

| Primer Name | Primer Sequence |
|---|---|
| **GNAS H5 FW** | 5’ CACACTTCACCTGTGCCACAGATACCGAGAACATCAGGCGCG 3’ |
| **GNAS XbaI RV** | 5’ TCTAGACTCGAGTCACAGCAGCTCATACTG 3’ |
| **KpnI GNAI FW** | 5’ GGTACCGCCACCATGGGATG 3’ |
| **GNAI H5 FW** | 5’ CACTTCACATGTGCCGTGGACACCAAGAATGTGCAGTTTGTGTTCG 3’ |
| **GNAI XbaI RV** | 5’ ATATATTCTAGACTCGAGTCAAAACAGCCCGC 3’ |
| **GNAS KpnI FW** | 5’ GGTACCGCCACCATGGGATG 3’ |
| **Gαi1-3 M1 LgBiT FW 1** | 5' GATCATCCACGAGGACGGCTACAGCGAGG 3' |
| **Gαi1-3 M1 LgBiT RV 1** | 5' CCTCGCTGTAGCCGTCCTCGTGGATGATC 3' |
| **Gαi1-3 M1 LgBiT FW 2** | 5' CAAGATCGACTTTGGCGATGCCGCCAGGGC 3' |
| **Gαi1-3 M1 LgBiT RV 2** | 5' GCCCTGGCGGCATCGCCAAAGTCGATCTTG 3' |
| **Gαi1-3 M2 LgBiT FW 1** | 5' CTCTTCGTGCTGGCAGGAAGCGCCGAGGAG 3' |

| | |
|---|---|
| **Gαi1-3 M2 LgBiT RV 1** | 5' CTCCTCGGCGCTTCCTGCCAGCACGAAGAG 3' |
| **Gαi1-3 M2 LgBiT FW 2** | 5' CCGAGGAGGGCGTTATGACCACCGAGCTGGCA 3' |
| **Gαi1-3 M2 LgBiT RV 2** | 5' TGCCAGCTCGGTGGTCATAACGCCCTCCTCGG 3' |
| **Gαi1-3 M2 LgBiT FW 3** | 5' GATCAAGAGGCTGTGGAGGGACGGTGGCGTGCAGGC 3' |
| **Gαi1-3 M2 LgBiT RV 3** | 5' GCCTGCACGCCAGCGTCCGCCCACAGCCTCTTG 3' |
| **Gαi1-3 M2 LgBiT FW 4** | 5' TGACAATCTGCTACCCCGAGTATACCGGCTCTAATA 3' |
| **Gαi1-3 M2 LgBiT RV 4** | 5' TATTAGAGCCGGTATACTCGGGGTAGCAGATTGTCA 3' |
| **Gαi1-3 M3 LgBiT FW 1** | 5' GTGTAGGGATATAGTTGCTCTGGGCGATGCGATCCA 3' |
| **Gαi1-3 M3 LgBiT RV 1** | 5' TGGATCGCATCGCCCAGAGCAACTATATCCCTACAC 3' |
| **Gαi1-3 M3 LgBiT FW 2** | 5' AGGACCCGCGTGAGGACCACAGGCATC 3' |
| **Gαi1-3 M3 LgBiT RV 2** | 5' GATGCCTGTGGTCCTCACGCGGGTCCT 3' |
| **GαsHD FW (For swap onto Gαi)** | 5' GATCATCCACGAGGCCGGCTACAGCGATGGCGAGAAGGCCACA 3' |
| **GαsHD RV (For swap onto Gαi)** | 5' CCTGTGGTCTTCACGCGGGTCCGCAGCAGATCCTGGTCAGAAG 3' |
| **GαiHD FW (For swap onto Gαs)** | 5' ACGGCTTCAATGGCGAGTCTAACAGCGAGGAGGAGTGCAAGCAGT 3' |
| **GαiHD RV (For swap onto Gαs)** | 5' ACGGCTTCAATGGCGAGTCTAACAGCGAGGAGGAGTGCAAGCAGT 3' |

**REFERENCES:**


1. Smith, J. S. *et al.* Noncanonical scaffolding of $G_{\alpha i}$ and β-arrestin by G protein–coupled receptors. *Science*, eaay1833 (2021). https://doi.org:10.1126/science.aay1833
2. Beaulieu, J. M. *et al.* An Akt/beta-arrestin 2/PP2A signaling complex mediates dopaminergic neurotransmission and behavior. *Cell* **122**, 261-273 (2005). https://doi.org:10.1016/j.cell.2005.05.012
3. Vural, A. *et al.* Distribution of activator of G-protein signaling 3 within the aggresomal pathway: role of specific residues in the tetratricopeptide repeat domain and differential regulation by the AGS3 binding partners Gi(alpha) and mammalian inscuteable. *Mol Cell Biol* **30**, 1528-1540 (2010). https://doi.org:10.1128/mcb.01018-09

4. Olsen, R. H. J. *et al.* TRUPATH, an open-source biosensor platform for interrogating the GPCR transducerome. *Nature Chemical Biology* **16**, 841-849 (2020). https://doi.org:10.1038/s41589-020-0535-8
5. Parag-Sharma, K. *et al.* Membrane Recruitment of the Non-receptor Protein GIV/Girdin (Gα-interacting, Vesicle-associated Protein/Girdin) Is Sufficient for Activating Heterotrimeric G Protein Signaling. *J Biol Chem* **291**, 27098-27111 (2016). https://doi.org:10.1074/jbc.M116.764431
6. Inoue, T., Heo, W. D., Grimley, J. S., Wandless, T. J. & Meyer, T. An inducible translocation strategy to rapidly activate and inhibit small GTPase signaling pathways. *Nat Methods* **2**, 415-418 (2005). https://doi.org:10.1038/nmeth763
