# Supplementary material for: β-arrestin recruitment facilitates a direct association with G proteins
Source: Nat Commun. 2026 Jul 17;17:8840. doi: 10.1038/s41467-026-74944-7 (PMC13500827; doi:10.1038/s41467-026-74944-7)
Supplement: Supplementary file 2 [file 41467_2026_74944_MOESM2_ESM.pdf]

nature portfolio

Corresponding author(s): Sudarshan Rajagopal, Andrew C. Kruse, Jeffrey S. Smith

Last updated by author(s): Jun 6 2026

Reporting Summary

Nature Portfolio wishes to improve the reproducibility of the work that we publish. This form provides structure for consistency and transparency in reporting. For further information on Nature Portfolio policies, see our Editorial Policies and the Editorial Policy Checklist.

Statistics

For all statistical analyses, confirm that the following items are present in the figure legend, table legend, main text, or Methods section.

n/a	Confirmed	
☐	☒	The exact sample size (n) for each experimental group/condition, given as a discrete number and unit of measurement
☐	☒	A statement on whether measurements were taken from distinct samples or whether the same sample was measured repeatedly
☐	☒	The statistical test(s) used AND whether they are one- or two-sided<br>*Only common tests should be described solely by name; describe more complex techniques in the Methods section.*
☒	☐	A description of all covariates tested
☐	☒	A description of any assumptions or corrections, such as tests of normality and adjustment for multiple comparisons
☐	☒	A full description of the statistical parameters including central tendency (e.g. means) or other basic estimates (e.g. regression coefficient) AND variation (e.g. standard deviation) or associated estimates of uncertainty (e.g. confidence intervals)
☐	☒	For null hypothesis testing, the test statistic (e.g. F, t, r) with confidence intervals, effect sizes, degrees of freedom and P value noted<br>*Give P values as exact values whenever suitable.*
☒	☐	For Bayesian analysis, information on the choice of priors and Markov chain Monte Carlo settings
☒	☐	For hierarchical and complex designs, identification of the appropriate level for tests and full reporting of outcomes
☒	☐	Estimates of effect sizes (e.g. Cohen's d, Pearson's r), indicating how they were calculated

Our web collection on statistics for biologists contains articles on many of the points above.

Software and code

Policy information about availability of computer code

Data collection	Plate reader data were collected by the following corresponding programs: Synergy-Neo2 Biotek plate reader (Gen 3.15), Berthold Mithras LB940 (Mithras 2010), Promega Glomax.
Data analysis	Data were analyzed via Excel (Microsoft, Redmond, WA) and/or Prism 10, and graphed in Prism 10 (GraphPad, San Diego, CA).

For manuscripts utilizing custom algorithms or software that are central to the research but not yet described in published literature, software must be made available to editors and reviewers. We strongly encourage code deposition in a community repository (e.g. GitHub). See the Nature Portfolio guidelines for submitting code & software for further information.

Data

Policy information about availability of data

All manuscripts must include a data availability statement. This statement should provide the following information, where applicable:

- Accession codes, unique identifiers, or web links for publicly available datasets
- A description of any restrictions on data availability
- For clinical datasets or third party data, please ensure that the statement adheres to our policy

All data are available in the manuscript or the supplementary materials and supporting data. Data and materials will be fully available upon reasonable request. Constructs designed in this study and that originated in the Rajagopal Lab (table S1) are available from S.R. under a materials transfer agreement with Duke University. Constructs designed in this study and that originated in the Kruse Lab (table S1) are available from A.C.K. under a materials transfer agreement with Harvard University. Graphical figures were created in part using BioRender.com.

Research involving human participants, their data, or biological material

Policy information about studies with human participants or human data. See also policy information about sex, gender (identity/presentation), and sexual orientation and race, ethnicity and racism.

Reporting on sex and gender	n/a
Reporting on race, ethnicity, or other socially relevant groupings	n/a
Population characteristics	n/a
Recruitment	n/a
Ethics oversight	n/a

Note that full information on the approval of the study protocol must also be provided in the manuscript.

Field-specific reporting

Please select the one below that is the best fit for your research. If you are not sure, read the appropriate sections before making your selection.

[x] Life sciences [ ] Behavioural & social sciences [ ] Ecological, evolutionary & environmental sciences

For a reference copy of the document with all sections, see nature.com/documents/nr-reporting-summary-flat.pdf

Life sciences study design

All studies must disclose on these points even when the disclosure is negative.

Sample size	A minimum sample size of at least n=3 was utilized for all assays (no calculations were made prior for determining sample size) to ensure sufficient sample size for statistical analysis. Additional samples were utilized in experiments where signal window was poor or where additional controls were needed
Data exclusions	Some data were excluded if known human error occurred for the specific replicate (e.g. pipetting error).
Replication	All experiments were replicated a minimum of 3 times. Each replicate constituted a completely independent experiment ran on separate days and, if possible, by another person to ensure reproducibility.
Randomization	Samples were organized into groups via treatment conditions, identity of DNA transfected (for cellular assays), and/or protein constructs expressed. No randomization occurred by the nature of our cellular experiments.
Blinding	Blinding was not utilized in our studies.

Reporting for specific materials, systems and methods

We require information from authors about some types of materials, experimental systems and methods used in many studies. Here, indicate whether each material, system or method listed is relevant to your study. If you are not sure if a list item applies to your research, read the appropriate section before selecting a response.

Materials & experimental systems

n/a	Involved in the study	
[ ]	[x]	Antibodies
[ ]	[x]	Eukaryotic cell lines
[x]	[ ]	Palaeontology and archaeology
[x]	[ ]	Animals and other organisms
[x]	[ ]	Clinical data
[x]	[ ]	Dual use research of concern
[x]	[ ]	Plants

Methods

n/a	Involved in the study	
[x]	[ ]	ChIP-seq
[x]	[ ]	Flow cytometry
[x]	[ ]	MRI-based neuroimaging

Antibodies

Antibodies used	Anti-Protein C antibodies were used in pull-down assays.
Validation	An anti-protein C secreting hybridoma is maintained by the Kruse laboratory and was initially obtain from the Laboratory of Daniel Hilger, Phillips-University of Marburg. This hybridoma has also been validated by sequencing (https://www.addgene.org/221361/).

Eukaryotic cell lines

Policy information about cell lines and Sex and Gender in Research

Cell line source(s)	HEK293T-17
Authentication	Cells in the Rajagopal and Kruse laboratory were purchased from ATCC
Mycoplasma contamination	Performed and negative in the Kruse Lab and Rajagopal Lab
Commonly misidentified lines (See ICLAC register)	n/a

Plants

Seed stocks	n/a
Novel plant genotypes	n/a
Authentication	n/a
