# Supplementary material for: β-arrestin recruitment facilitates a direct association with G proteins
Source: Nat Commun. 2026 Jul 17;17:8840. doi: 10.1038/s41467-026-74944-7 (PMC13500827; doi:10.1038/s41467-026-74944-7)
Supplement: Supplementary file 3 [file 41467_2026_74944_MOESM3_ESM.pdf]

nature portfolio

Peer Review File

β-arrestin recruitment facilitates a direct association with G proteins

Corresponding Author: Dr Jeffrey Smith

This file contains all reviewer reports in order by version, followed by all author rebuttals in order by version.

Version 0:

Reviewer comments:

Reviewer #1

(Remarks to the Author)
In this manuscript the authors continue their characterization of interactions between Galpha subunits and beta arrestins. The main claims are that 1) G proteins and arrestins can interact directly, shown using purified proteins, 2) concentration of arrestin on the plasma membrane is sufficient to promote the interaction, shown using arrestin-biased receptors and induced dimerization, and 3) there are differences between Galphai subunits, and these can be attributed to their alpha helical domains. The results generally support these claims, but none of the claims is a significant conceptual advance and many are not novel.

Fig. 3L in the authors' 2021 Science paper shows pulldown of purified Galphai by arrestin3. The current Fig. 5 is a very similar experiment using a truncated version of arrestin2 and shows insensitivity to nucleotide, but since Galphai activation has already been shown to be inessential for the interaction (Fig. 3G of the same paper and elsewhere) this is unsurprising. Notably arrestin2 (beta-arrestin1) is not used for any other experiment in the present manuscript.

Likewise the 2021 paper showed that TRV-activated angiotensin receptors did not activate G proteins but did promote Gi-arrestin interaction, essentially the same experiment repeated here with different arrestin-biased tools. The previous paper also showed that G protein activation alone was insufficient to promote the interaction, repeated here with G protein-biased receptors. The induced dimerization approach used in the present manuscript is new, but again not a conceptual advance since it was shown previously that the proteins interact in vitro and in cells when concentrated on a membrane. The group's 2022 Science Signaling paper also focused on biased ligands and showed a strong correlation between arrestin recruitment and Gi-arrestin interaction, despite a statement to the contrary in the abstract.

Fig. S7 and Fig. 3 of the 2021 paper showed differences between the same Galphai subunits studied here. The present manuscript shows loss of function with some mutations that correspond to the less-favored Galphai subtypes, suggesting the alpha helical region is responsible for the binding. However, there is no corresponding gain of function for the switched alpha helical domain (Fig. S6 of the present manuscript) and a lack of rigorous controls for mutant protein expression and localization. Additional experiments would be required to make this conclusion convincing.

One of the other major shortcomings of the work is exclusive reliance on overexpressed components fused to complementing protein fragments or concentrated proteins in vitro. From this group's previous two papers it was clear that there is weak binding of arrestin to Galphai subunits that can be detected using the methods used here, and the present manuscript doesn't change this picture appreciably. The significance of this binding interaction when native receptors recruit native arrestins to membranes containing native G proteins is much less clear and is not addressed here.

Fig. 1 uses M3 muscarinic receptors to "re-establish" the group's previous findings. This figure doesn't add anything, especially since this receptor is never used again.

Recruitment assays comparing different LgBiT-tagged alpha subunits (Fig. 4 and Fig. 6 and supplement) need to be controlled for different Galpha-LgBit expression and colocalization with either receptors or FRB. Titration experiments with different amounts of plasmid DNA are not adequate to rule out differences in expression level or localization.

The manuscript relies heavily on data that are normalized, sometimes in an unclear manner. For example, Fig. S5 shows

response with different amounts of transfected Galphai DNA normalized to the maximum luminescence observed with 100ng, but somehow all of the responses start from the exact same baseline (~70%) even though baseline luminescence was shown previously to be quite sensitive to DNA amount (Fig. S5 of the 2021 paper)? In Fig. 2 panels A-C are normalized as % increase above vehicle, whereas panel D is normalized to 100% of the maximum luminescence, making it impossible to compare these responses. Curves in panel D start at 0, suggesting either that there was zero background luminescence, or that the y axis is actually normalized to the maximum change in luminescence.

Most of the figures report only 3 replicates, and "replicates" is not clearly defined. Parametric statistical comparisons are often performed on data where n=3, which is of little value.

Reviewer #2

(Remarks to the Author)
This study by Lee et al. elegantly demonstrates a direct interaction between Gαi and β-arrestin at the plasma membrane, significantly advancing our understanding of GPCR signaling by challenging the traditional view of independent pathway operation. The authors use a series of innovative and well-controlled experiments—including β-arrestin-biased receptors, chemically inducible heterodimerization, and direct protein interaction assays—to convincingly show that β-arrestin plasma membrane localization alone is sufficient for complex formation, independent of classical G protein activation and independent of β-arrestin recruitment to a specific GPCR. Their mechanistic insights are strengthened by systematic mutagenesis, revealing a role of the Gαi alpha helical domain in binding selectivity. While the study is rigorous and thorough in its analysis of Gαi:β-arrestin complex formation, it leaves applicability to other G protein families and the cellular and physiological consequences of complex formation as avenues for future investigation. The clarity of the findings and the methodological sophistication make this work a valuable contribution to the field, setting the stage for future studies on the functional impact of Gαi:β-arrestin cross-talk and its potential therapeutic implications.

I have several specific comments:

1. The authors conclude the abstract with the statement that "These findings delineate the cellular and biological conditions that drive direct interactions between G proteins and β-arrestins and illuminate the molecular basis for how they work together to effect GPCR signaling." While the studies reveal some of the conditions that are sufficient (e.g., membrane localization) and necessary (e.g., intact Gαi alpha helical domain) for Gαi:β-arrestin complex formation, this statement may lead readers to expect an investigation into GPCR signaling.
2. In the introduction, it would be helpful if the authors could more clearly delineate what is already known about Gαi:β-arrestin complex formation. For example, how was the Gαi:β-arrestin complex previously demonstrated? Where prior observations of complex formation based exclusively on proximity-based assessments? What was the alternative to a direct Gαi:β-arrestin interaction?
3. Figure 1A shows M3R ligand-induced association between β-arrestin and Gαi but not Gαq or Gαs. How confident are the authors that β-arrestin:Gαq and β-arrestin:Gαs complexes do not form? Were attempts to identify such complexes made by expressing different amounts of the Gα, moving the location (i.e., amino acid position) of the LgBiT tag, using an alternative proximity-based assay, or performing immunoprecipitations? Relatedly, does adding the LgBiT tag differentially change expression levels or function of the Gα proteins? If these experiments were conducted previously and presented elsewhere, it would be helpful if the authors could reference this work.
4. Figure 1C shows Gαi:β-arrestin complex formation following M3R activation by different agonists. Why is the extent of complex formation greater with iperoxo, as compared to carbachol and oxotremorine M? Is the extent of ligand-induced complex formation proportional to the extent of ligand-induced β-arrestin recruitment to the receptor / translocation to the plasma membrane?
5. In Figure 2, what is the signaling profile of D2R-ARB? It would be helpful if the authors could include this or clearly reference whether the signaling profile has been previously presented. A thorough assessment would include multiple Gi/o G protein family members and a description of what if any G protein activation is retained.
6. In Figure 2, Gq and Gs are presented seemingly as negative controls, but their data are difficult to interpret without understanding how rigorously an association between β-arrestin and these G proteins was investigated. That said, these data do show that the interaction is not tag-driven or non-specific.
7. It would help the reader if the authors could justify their choice of Gq vs Gs for inclusion in the studies presented in Figure 2 (i.e., inclusion of Gq but not Gs in panels A and B and Gs but not Gq in panel C).
8. On page 7, line 145, fentanyl is referred to as a balanced agonist. Others have reported fentanyl to show a preference for β-arrestin activation (Schimid et al. 2017. Volume 171, Issue 5, 16 November 2017, Pages 1165-1175.e13Bias Factor and Therapeutic Window Correlate to Predict Safer Opioid Analgesics). While certainly more balanced than other newly develop MOR ligands, can the authors clarify and contextualize the statement that fentanyl is balanced?
9. In Figure 3, no β-arrestin recruitment is identified with oliceridine. This is in contrast to other reports for this ligand, which show some degree of preserved β-arrestin recruitment. It is not a major concern, as differences in assay design and sensitivity will drive detection, but can the authors comment on differences in the assay platforms that may account for this apparent discrepancy?
10. The studies in Figure 3 presumably required expression of the mu opioid receptor (MOR), but this not mentioned in the results, legends, or methods. MOR does appear in the construct list. Could the authors please add information on how these studies were performed to the Methods and reference MOR expression in the results and/or figure legend?
11. Related to the finding that β-arrestin translocation to the plasma membrane is sufficient to promote Gαi:β-arrestin in Figure 4. It would strengthen the authors' argument to demonstrate that rapamycin does indeed drive β-arrestin recruitment

to the plasma membrane using an orthogonal assay. Perhaps increased association with a CAAX- or MyrPalm-tethered sensor or via microscopy.
12. The identification of a direct interaction between Gi1 and β-arrestin1 in Figure 5 is a major finding. This set for studies, however, would benefit from additional explanation and controls. While the representative gel image is nice, density quantification that includes data from multiple biological replicates would increase confidence. Did the authors consider normalizing to β-arrestin? Looking at the relative densities of the Gi input lane and rest of the gel, was the extent of complex formation in line with expectations? Why switch from β-arrestin-2, with which all cellular complex formation experiments were conduction, to β-arrestin-1 here? The authors should justify this choice and, ideally, include cellular complex formation studies with β-arrestin-1. And, perhaps most importantly, how does the extent of complex formation with Gi:β-arrestin-1 as compared to Gq: β-arrestin-1 and/or Gs:β-arrestin-1?
13. The authors mention that βγ is not required for Gi:β-arrestin complex formation, however, the binding in the pulldowns appear relatively small small. Have the authors considered adding βγ to these pulldowns assays to see if this either increases or decreases binding? This would clarify the role of βγ in this interaction.
14. With Figure 6, I again wonder about attempts to optimize this association assay for other Gi family members. Also, could the reduction in BRET with these mutants be the result of different complex conformation that results in a greater distance between mKO and Nluc rather than a reduction in total complex formation?
15. It appears many different species of β-arrestin protein used throughout the work (mouse for BRET, rat or bovine for purification) and that the species did not match that of the G proteins, which were human. The authors should explain these choices and
16. As I'm sure the authors have considered, it would be fascinating - and further increase the impact of this work - to evaluate the cellular consequences of these alpha helical domain mutations and to determine if these consequences are attributable to a change in the formation or signaling competency of the Gi:β-arrestin complex. In general, evaluating the signaling consequences of complex formation or inhibition would help frame the significance of the interaction.

Minor
1. The first time FKBP/FRB appears on Page 5, the abbreviation is left undefined.
2. For simplicity, it looks like several panels in Figure 2 could be combined, for example 2A and 2B could be combined into a single panel. 2D and 2E could be combined into a single panel. 2F, 2G, and 2H could be combined into a single panel. This would reduce the total panels in Figure 2 from 8 to 4 and may ease reader interpretation.
3. Error bars in Figure 5B are SD while for the rest of the figures they are SEM. Could the authors justify the use of SD for this panel or bring it into alignment with the rest of the figures?
4. Figure 6 is missing panel numbers.
5. The species of the ACKR3 construct is missing from Table 1.

I hope these suggestions, on an already thorough manuscript, will provide relevant feedback to the authors. I remain at the disposal of the editor and the authors for further clarifications.

Reviewer #3

(Remarks to the Author)
Lee, Rajagopal and colleagues provide evidence supporting the ability of beta-arrestin to associate with Gi/o at the plasm membrane, as this group has previously proposed, and they extend the work by demonstrating a specific in vitro association with purified proteins and identifying the G protein N-terminal helix as an important structural determinant of the interaction. The data are generally convincing and the interpretations are reasonable. I'm left being convinced that the interaction demonstrated probably does occur in cells, but unclear about what functional significance this interaction has.

I found the data generally convincing and have only a couple of specific points that would benefit from comment or clarification..

1. The interaction in cells is demonstrated primarily using beta-arrestin-2 but I'm unclear about what interaction is measured in vitro in Fig 5. The figure indicates barr1 in panel A and barr1/2 in B. Can the autjhors clarify? Is barr1/2 a chimera or what? Can the authors comment on or clarify if there are differences, in cells or in vitro, in Gi/o association with beta-arrestin 1 compared to 2?

2. I may have missed this in this manuscript or previous publications, but can the authors comment on what functional significance they attribute to the beta-arrestin:G protein interaction. The authors show clearly that the interaction depends on membrane recruitment of beta-arrestin but not GPCR-G protein coupling. They also show recruitment by AVPR2 and I don't believe this receptor couples to Gi/o. What functional consequence do the authors think that this recruitment has?

Version 1:

Reviewer comments:

Reviewer #1

(Remarks to the Author)
The manuscript by Lee et al. has not changed substantially from the initial submission. Instead, the authors argue in their rebuttal that they have performed new experiments and expanded their toolset and make several conceptual advances. I

agree that they have shown new data here and have used new tools, but I disagree that the conceptual advances are significant. The main findings as outlined in the abstract are:

“Here we show that G proteins and beta-arrestins can directly interact.”

The abstract from their 2021 paper states “we found that GPCRs can catalyze a direct interaction between the Galphai protein subtype and beta-arrestins”. Figure 3L in the 2021 paper shows a direct interaction between purified proteins.

“Through utilization of beta-arrestin-biased receptors and artificially enforced beta-arrestin relocalization we demonstrate that recruitment of beta-arrestin to the plasma membrane is sufficient to interact with the G protein Galphai.”

Several of the experiments in the previous two papers demonstrate the same concept, including receptor-ligand combinations that do not activate G proteins, PTX, etc. It must also be reiterated that if putting two proteins together in a test tube is sufficient to promote the interaction then concentrating them on a membrane is expected to do so as well.

“Using purified proteins, we show that Galphai directly interacts with beta-arrestin.”

See above.

“In addition, we find that Galphai family members differ in their degree of association with beta-arrestin,”

This was amply demonstrated in the original 2021 paper with the same G proteins studied here.

“and that a large degree of this selectivity resides within the alpha helical domain of Galphai.”

This is new but given the almost perfect identity of the Galphai isoforms outside of the alpha helical domain there was little alternative. Loss of function is still shown without reciprocal gain of function, and n is still 3 for this and many other experiments.

The data contained in this manuscript largely confirm this group’s previous findings and a few small gaps are filled in, but the overall model hasn’t changed significantly. The conceptual advance does not rise to the standard of Nature Communications.

Reviewer #2

(Remarks to the Author)
The authors addressed most critiques with the addition of control experiments, references to published work, and refinement of the text. As noted previously, the work convincingly demonstrates that the beta-arrestin and Gi interaction is direct and identifies some of its molecular determinants.

A few concerns remain:

1) The largest concern is the lack of functional data demonstrating why this interaction is of biological significance.

2) The possibility remains that the effect identified in Figure 6H is driven by differences in construct expression. In lines 255-256 the authors state that "all mutants were tagged with a full-length luciferase (NanoLuc) for use in a NanoBRET assay and to ensure expression differences did not account for variation in signal." However, it is not clear that this expression data is included in the paper. As reduced expression can drive reductions in max delta net BRETs, these data are necessary to rule out expression differences as a confounder in these studies and support the stated conclusion.

3) The new Supplemental Figure 6I plot is challenging to interpret as presented, with the data for the Gs with I Helix 5 entirely obscured.

4) The authors reference a co-submitted manuscript that supports an interaction motif in the alpha helical domain. Without access to this manuscript we cannot assess this claim.

5) For transparency, the bar graphs in main text Figures 5B and 6I and Supplemental Figures 1D, 5B and 5D should include experimental replicates as symbols.

Reviewer #3

(Remarks to the Author)
The authors have sufficiently addressed my previous concerns and they have clarified the revised manuscript accordingly.

Reviewer #4

(Remarks to the Author)
I co-reviewed this manuscript with one of the reviewers who provided the listed reports. This is part of the Nature

Communications initiative to facilitate training in peer review and to provide appropriate recognition for Early Career Researchers who co-review manuscripts.

Version 2:

Reviewer comments:

Reviewer #2

(Remarks to the Author)
The authors have sufficiently addressed my earlier concerns, and the manuscript has been revised accordingly.

Open Access This Peer Review File is licensed under a Creative Commons Attribution 4.0 International License, which permits use, sharing, adaptation, distribution and reproduction in any medium or format, as long as you give appropriate credit to the original author(s) and the source, provide a link to the Creative Commons license, and indicate if changes were made.
In cases where reviewers are anonymous, credit should be given to 'Anonymous Referee' and the source.
The images or other third party material in this Peer Review File are included in the article's Creative Commons license, unless indicated otherwise in a credit line to the material. If material is not included in the article's Creative Commons license and your intended use is not permitted by statutory regulation or exceeds the permitted use, you will need to obtain permission directly from the copyright holder.
To view a copy of this license, visit https://creativecommons.org/licenses/by/4.0/

We thank the reviewers for their careful evaluation of our manuscript. Below, we include the reviewers' comments in regular type and our responses in bold.

Reviewer #1 (Remarks to the Author):

In this manuscript the authors continue their characterization of interactions between Galpha subunits and beta arrestins. The main claims are that 1) G proteins and arrestins can interact directly, shown using purified proteins, 2) concentration of arrestin on the plasma membrane is sufficient to promote the interaction, shown using arrestin-biased receptors and induced dimerization, and 3) there are differences between Galphai subunits, and these can be attributed to their alpha helical domains. The results generally support these claims, but none of the claims is a significant conceptual advance and many are not novel.

We thank the reviewer for their careful reading of this manuscript and our prior contributions on this interaction. We note that at multiple conferences questions arise regarding the biochemical validation of a Gi:beta-arrestin interaction. Indeed, at the most recent Gordon Research Molecular Pharmacology conference, the 2021 *Science* paper was directly referenced as intriguing but requiring further biochemical data and validation. G proteins and beta-arrestins are the two most studied proximal signaling effectors of GPCRs, and we believe that a direct interaction between the two requires rigorous dissection that is of high interest to the field. The reviewer appropriately notes that we have published two papers focused on the breadth of interaction at a number of GPCRs and functional consequences (cell migration, ERK phosphorylation) (Smith and Pack, 2021) and a characterization of a single Gi-coupled receptor CXCR3 with multiple chemokines and small molecules (Zheng and Smith, 2022), which we summarized and cited frequently throughout the text.

The current manuscript delineates the following advances involving both technical and conceptual novelty (1) beta-arrestin recruitment to the plasma membrane is sufficient to promote the interaction regardless of Gi activation status (new finding) (2) the region of the G protein required to interact with beta-arrestin (new finding, and in near complete agreement with another independent manuscript being considered), (3) nucleotide status of the G protein not essential for the interaction (new finding, aligning with binding domain findings) and (4) a gold-standard approach showing that beta-arrestin does not act as a guanine exchange factor (GEF) or GTPase; important experiments to conduct given that some proteins which directly interact with G proteins act as GEFs or GTPases. We utilize a panel of tools to reach these conclusions.

Fig. 3L in the authors' 2021 Science paper shows pulldown of purified Galphai by arrestin3. The current Fig. 5 is a very similar experiment using a truncated version of arrestin2 and shows insensitivity to nucleotide, but since Galphai activation has already been shown to be inessential for the interaction (Fig. 3G of the same paper and

elsewhere) this is unsurprising. Notably arrestin2 (beta-arrestin1) is not used for any other experiment in the present manuscript.

Figure 3G in the 2021 shows that that the interaction is sensitive to pertussis toxin in HEK293T cells. This suggests a direct interaction, but does not define the nucleotide status (2021 panels G and H from the 2021 paper are copied for reference below). In the current manuscript we use purified protein to define the interaction status in the presence of GDP, GTPγS, and apyrase (nucleotide free) and also show that the interaction has no impact on GTPγS incorporation into the G protein. These are all new experiments. For thoroughness, we used both beta-arrestin2 and beta-arrestin1 are included in the GTP incorporation experiments.

We have also added a supplemental figure using beta-arrestin1 in BRET experiments (new Supplemental Figure 1B).

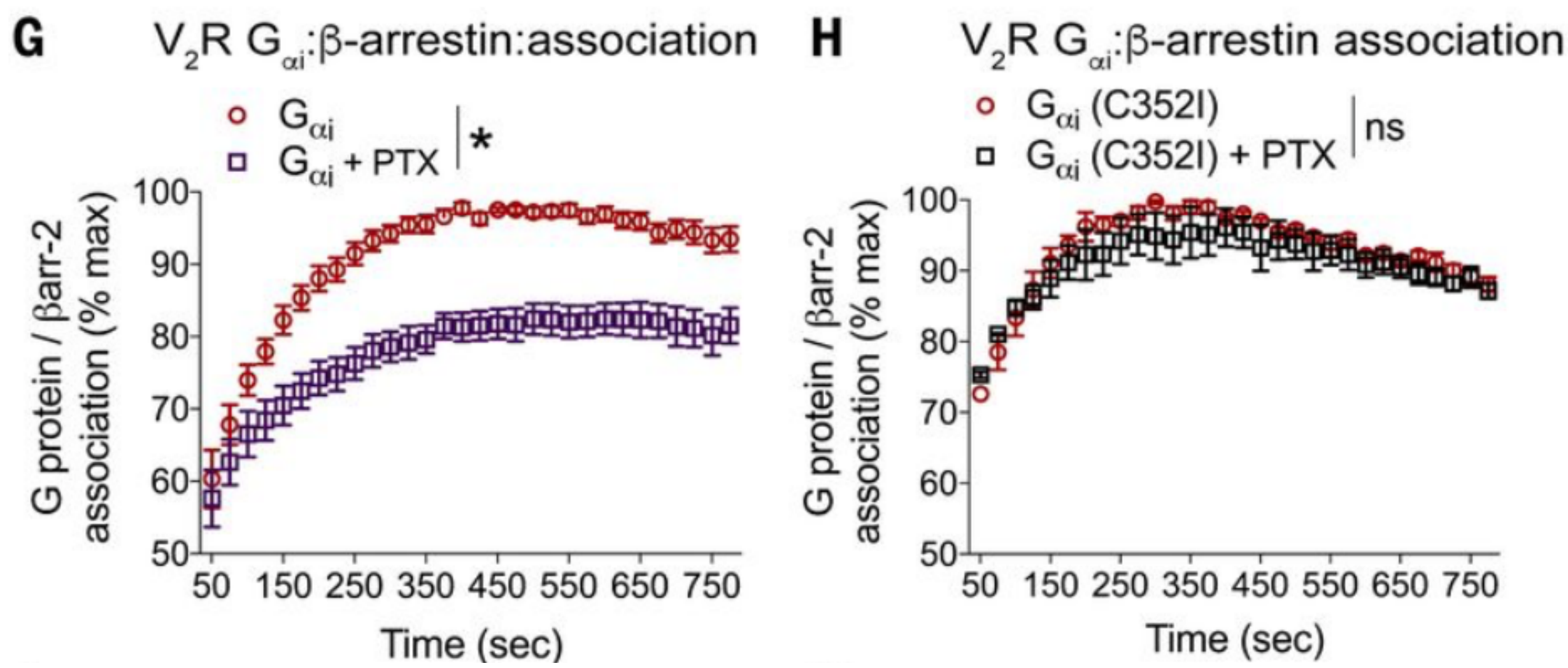

Likewise the 2021 paper showed that TRV-activated angiotensin receptors did not activate G proteins but did promote Gi-arrestin interaction, essentially the same experiment repeated here with different arrestin-biased tools.

While the 2021 paper was an initial characterization of this Gi:beta-arrestin interaction, the TRV023 experiments were conducted primarily to probe function in cell migration. The current study we expanded our toolset using both biased ligands and multiple biased receptors, with quantification of multiple pathways. Here, we dissected mechanism using multiple different biased receptors, biased ligands, and the FKBP system, all of which are novel and shows that recruitment to the membrane is sufficient for the interaction. Additionally, the inclusion of ACKR3, an endogenous beta-arrestin-biased receptor that is structurally suggested to be incapable of binding G proteins, was very helpful to confirm that both activation and recruitment of G protein are not required for association. This is in comparison to receptors like V2R that we knew did not activate Gi but can promote some Gi recruitment.

The previous paper also showed that G protein activation alone was insufficient to promote the interaction, repeated here with G protein-biased receptors.

We did not previously utilize fully G protein biased agonists in either the 2021 Science or 2022 Science signaling papers, or any direct activators of G protein such as aluminum fluoride. We are unaware of previously published experiments that directly test this.

The induced dimerization approach used in the present manuscript is new, but again not a conceptual advance since it was shown previously that the proteins interact in vitro and in cells when concentrated on a membrane. The group's 2022 Science Signaling paper also focused on biased ligands and showed a strong correlation between arrestin recruitment and Gi-arrestin interaction, despite a statement to the contrary in the abstract.

In our prior 2022 Science Signaling manuscript we did observe a positive correlation between beta-arrestin recruitment and the interaction. This laid the groundwork for experiments within the current study to flesh out the mechanistic details in which components were required (beta-arrestin recruitment, G protein activation, or both), as these requirements were unclear to us and many in the field we spoke with. We believe the current study is a significant conceptual (and arguably technical) advance in the mechanism of complex formation.

Fig. S7 and Fig. 3 of the 2021 paper showed differences between the same Galphai subunits studied here. The present manuscript shows loss of function with some mutations that correspond to the less-favored Galphai subtypes, suggesting the alpha helical region is responsible for the binding. However, there is no corresponding gain of function for the switched alpha helical domain (Fig. S6 of the present manuscript) and a lack of rigorous controls for mutant protein expression and localization. Additional experiments would be required to make this conclusion convincing.

We have now included expression data for these constructs through multiple approaches (Supplemental Figure 1, Supplemental Figure 6 panel I). In Supplemental Figure 6 panels A-D and Supplemental Figure 7 panels D-G we show that the alpha 5 helix swaps and the helical domain swaps, respectively, are functional as assessed by the ability of Gα to dissociate from Gβγ a TRUPATH assay (although not to the same degree as WT Gs or Gi, as might be expected).

These findings are also in line with the findings in a co-submitted manuscript from Duan et al "Interactions between β-Arrestin 1 and Gαs or Gαi1: Functional and structural insights" where both the Ras domain and helical domain for Gi1 were found to have contributions towards beta-arrestin 1 binding in a purified setting. Additionally, another study by Lefevre et al. in JBC 2024 (https://pubmed.ncbi.nlm.nih.gov/38522511/) showed that interactions between the Ras and helical domains of Gi were crucial in differentiating downstream

signaling profiles of Gi2 from other Gi family members. In short, interactions with both domains are likely.

One of the other major shortcomings of the work is exclusive reliance on overexpressed components fused to complementing protein fragments or concentrated proteins in vitro. From this group's previous two papers it was clear that there is weak binding of arrestin to Galphai subunits that can be detected using the methods used here, and the present manuscript doesn't change this picture appreciably. The significance of this binding interaction when native receptors recruit native arrestins to membranes containing native G proteins is much less clear and is not addressed here.

A limitation of many studies in GPCR signaling is the incorporation of tags that often rely on non-endogenous expression conditions. These limitations are noted in the discussion, page 14-15 lines 433-438, tracked changes version. Nevertheless, key findings within receptor biology have been discovered in overexpressed systems, and determining the interaction sites and key cellular steps required for the interaction will allow us to design appropriate experiments to test function in future studies with endogenous expression.

Fig. 1 uses M3 muscarinic receptors to "re-establish" the group's previous findings. This figure doesn't add anything, especially since this receptor is never used again.

We thank the reviewer for reading our prior work. However, we recognize that for the broad audience of *Nature Communications* most readers will not be as familiar with our prior two papers on this interaction (although we cite them throughout the manuscript). The muscarinic acetylcholine 3 receptor (M3R) is only the second primarily Gq-coupled receptor probed to form this Gi:beta-arrestin interaction, to our knowledge, and we believe an experiment worthy of inclusion.

Recruitment assays comparing different LgBiT-tagged alpha subunits (Fig. 4 and Fig. 6 and supplement) need to be controlled for different Galpha-LgBit expression and colocalization with either receptors or FRB. Titration experiments with different amounts of plasmid DNA are not adequate to rule out differences in expression level or localization.

We have now included western blot data for LgBiT Ga expression (Supplemental Figure 1 panels C,D) and incubation with various concentrations of HiBiT after cell lysis (Supplemental Figure 6 I). These orthogonal approaches to assess G protein levels show that expression differences between families, isoforms, and/or G protein chimeras do not account for differences in observed association with beta-arrestin.

The manuscript relies heavily on data that are normalized, sometimes in an unclear manner. For example, Fig. S5 shows response with different amounts of transfected Galphai DNA normalized to the maximum luminescence observed with 100ng, but

somehow all of the responses start from the exact same baseline (~70%) even though baseline luminescence was shown previously to be quite sensitive to DNA amount (Fig. S5 of the 2021 paper)? In Fig. 2 panels A-C are normalized as % increase above vehicle, whereas panel D is normalized to 100% of the maximum luminescence, making it impossible to compare these responses. Curves in panel D start at 0, suggesting either that there was zero background luminescence, or that the y axis is actually normalized to the maximum change in luminescence.

We have standardized our NanoBiT graphs to normalize signal as "% increase over vehicle" where appropriate.

Most of the figures report only 3 replicates, and "replicates" is not clearly defined. Parametric statistical comparisons are often performed on data where n=3, which is of little value.

Replicates refers to three independent reproductions of the same experiment (e.g., 3 plates for a cellular assay, 3 pull-downs for a pull-down experiment).

Reviewer #2 (Remarks to the Author):

This study by Lee et al. elegantly demonstrates a direct interaction between Gαi and β-arrestin at the plasma membrane, significantly advancing our understanding of GPCR signaling by challenging the traditional view of independent pathway operation. The authors use a series of innovative and well-controlled experiments—including β-arrestin-biased receptors, chemically inducible heterodimerization, and direct protein interaction assays—to convincingly show that β-arrestin plasma membrane localization alone is sufficient for complex formation, independent of classical G protein activation and independent of β-arrestin recruitment to a specific GPCR. Their mechanistic insights are strengthened by systematic mutagenesis, revealing a role of the Gαi alpha helical domain in binding selectivity. While the study is rigorous and thorough in its analysis of Gαi:β-arrestin complex formation, it leaves applicability to other G protein families and the cellular and physiological consequences of complex formation as avenues for future investigation. The clarity of the findings and the methodological sophistication make this work a valuable contribution to the field, setting the stage for future studies on the functional impact of Gαi:β-arrestin cross-talk and its potential therapeutic implications.

We thank the reviewer for highlighting the innovation and design of our study. **Future work will further dissect functional impact, particularly under endogenous conditions.**

I have several specific comments:

1. The authors conclude the abstract with the statement that "These findings delineate the cellular and biological conditions that drive direct interactions between G proteins and β-arrestins and illuminate the molecular basis for how they work together to effect

GPCR signaling." While the studies reveal some of the conditions that are sufficient (e.g., membrane localization) and necessary (e.g., intact Gαi alpha helical domain) for Gαi:β-arrestin complex formation, this statement may lead readers to expect an investigation into GPCR signaling.

We have adjusted the abstract accordingly.

2. In the introduction, it would be helpful if the authors could more clearly delineate what is already known about Gαi:β-arrestin complex formation. For example, how was the Gαi:β-arrestin complex previously demonstrated? Where prior observations of complex formation based exclusively on proximity-based assessments? What was the alternative to a direct Gαi:β-arrestin interaction?

We have expanded the introduction for a more in-depth summary of prior work on this interaction (pages 4 and 5, lines 90-100). The interaction was previously defined primarily in cell-based assays using confocal, split luciferase, and BRET-based approaches with a pull-down experiment also supporting a direct interaction. Functional outcomes in terms of ERK phosphorylation and cell migration were also a focus of the initial work, but the molecular basis and mechanistic requirements for complex formation were unclear. The alternative to a direct interaction was something like the observed megaplex, where a GPCR or other protein is required to colocalize Gai and beta-arrestin.

3. Figure 1A shows M3R ligand-induced association between β-arrestin and Gαi but not Gαq or Gαs. How confident are the authors that β-arrestin:Gαq and β-arrestin:Gαs complexes do not form? Were attempts to identify such complexes made by expressing different amounts of the Gα, moving the location (i.e., amino acid position) of the LgBiT tag, using an alternative proximity-based assay, or performing immunoprecipitations?

In our initial study of this Gi:beta-arrestin interaction we used multiple different tags; a screen shot of the pertinent panels from supplemental Figure S9A using BRET as opposed to split luciferase is included after treatment of the V2R with a saturating concentration of AVP; no associated with Gs was observed. We have clarified these previous findings in the introduction (page 4-5, line 90-99, tracked changes version) and included screen shots below from a prior paper conducting these controls (DOI: 10.1126/science.aay183) for reference.

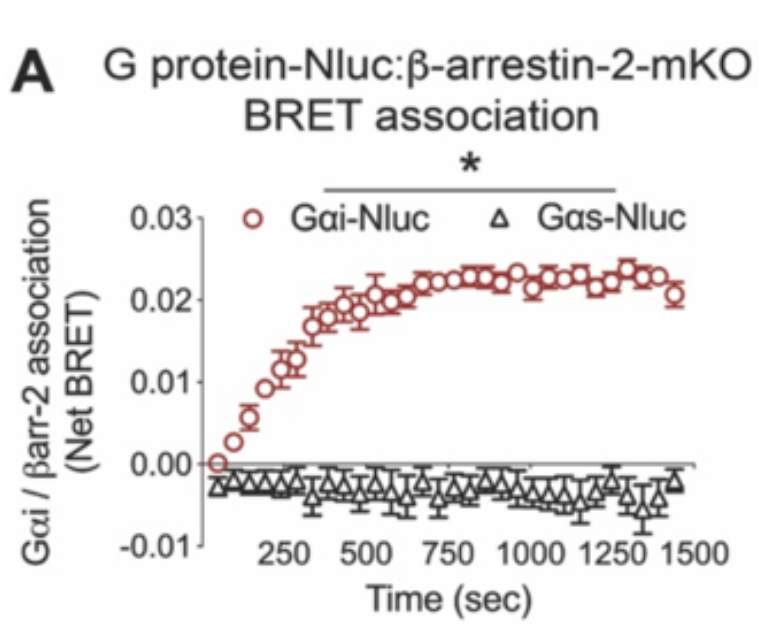
A
G protein-Nluc:β-arrestin-2-mKO
BRET association
*
Gαi-Nluc
Gαs-Nluc
Gαi / βarr-2 association (Net BRET)
Time (sec)

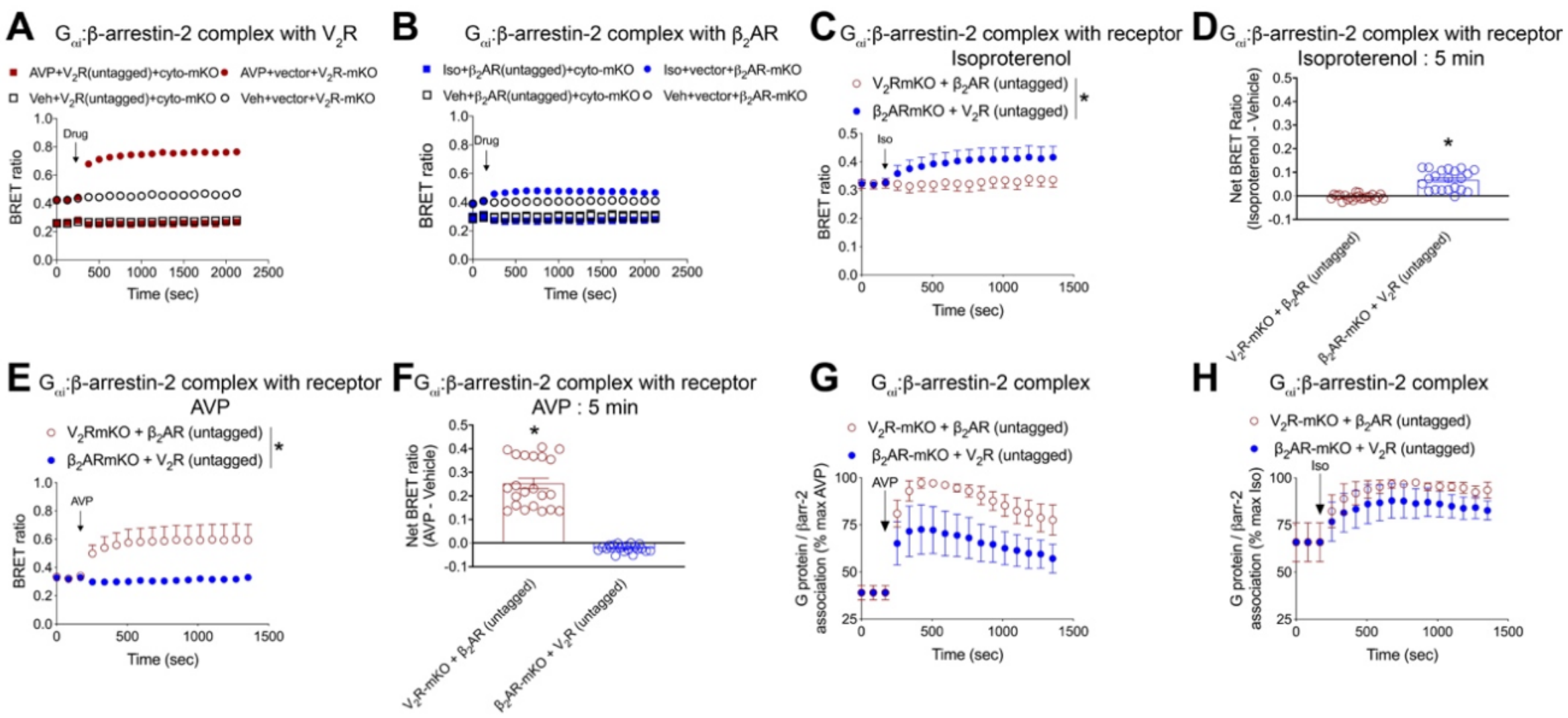
A
Gαi:β-arrestin-2 complex with V2R
AVP+V2R(untagged)+cyto-mKO
AVP+vector+V2R-mKO
Veh+V2R(untagged)+cyto-mKO
Veh+vector+V2R-mKO
Drug
BRET ratio
Time (sec)
B
Gαi:β-arrestin-2 complex with β2AR
Iso+β2AR(untagged)+cyto-mKO
Iso+vector+β2AR-mKO
Veh+β2AR(untagged)+cyto-mKO
Veh+vector+β2AR-mKO
Drug
BRET ratio
Time (sec)
C
Gαi:β-arrestin-2 complex with receptor
Isoproterenol
V2RmKO + β2AR (untagged)
β2ARmKO + V2R (untagged)
*
Iso
BRET ratio
Time (sec)
D
Gαi:β-arrestin-2 complex with receptor
Isoproterenol : 5 min
Net BRET Ratio (Isoproterenol - Vehicle)
*
V2R-mKO + β2AR (untagged)
β2AR-mKO + V2R (untagged)
E
Gαi:β-arrestin-2 complex with receptor
AVP
V2RmKO + β2AR (untagged)
β2ARmKO + V2R (untagged)
*
AVP
BRET ratio
Time (sec)
F
Gαi:β-arrestin-2 complex with receptor
AVP : 5 min
Net BRET ratio (AVP - Vehicle)
*
V2R-mKO + β2AR (untagged)
β2AR-mKO + V2R (untagged)
G
Gαi:β-arrestin-2 complex
V2R-mKO + β2AR (untagged)
β2AR-mKO + V2R (untagged)
AVP
G protein / βarr-2 association (% max AVP)
Time (sec)
H
Gαi:β-arrestin-2 complex
V2R-mKO + β2AR (untagged)
β2AR-mKO + V2R (untagged)
Iso
G protein / βarr-2 association (% max Iso)
Time (sec)

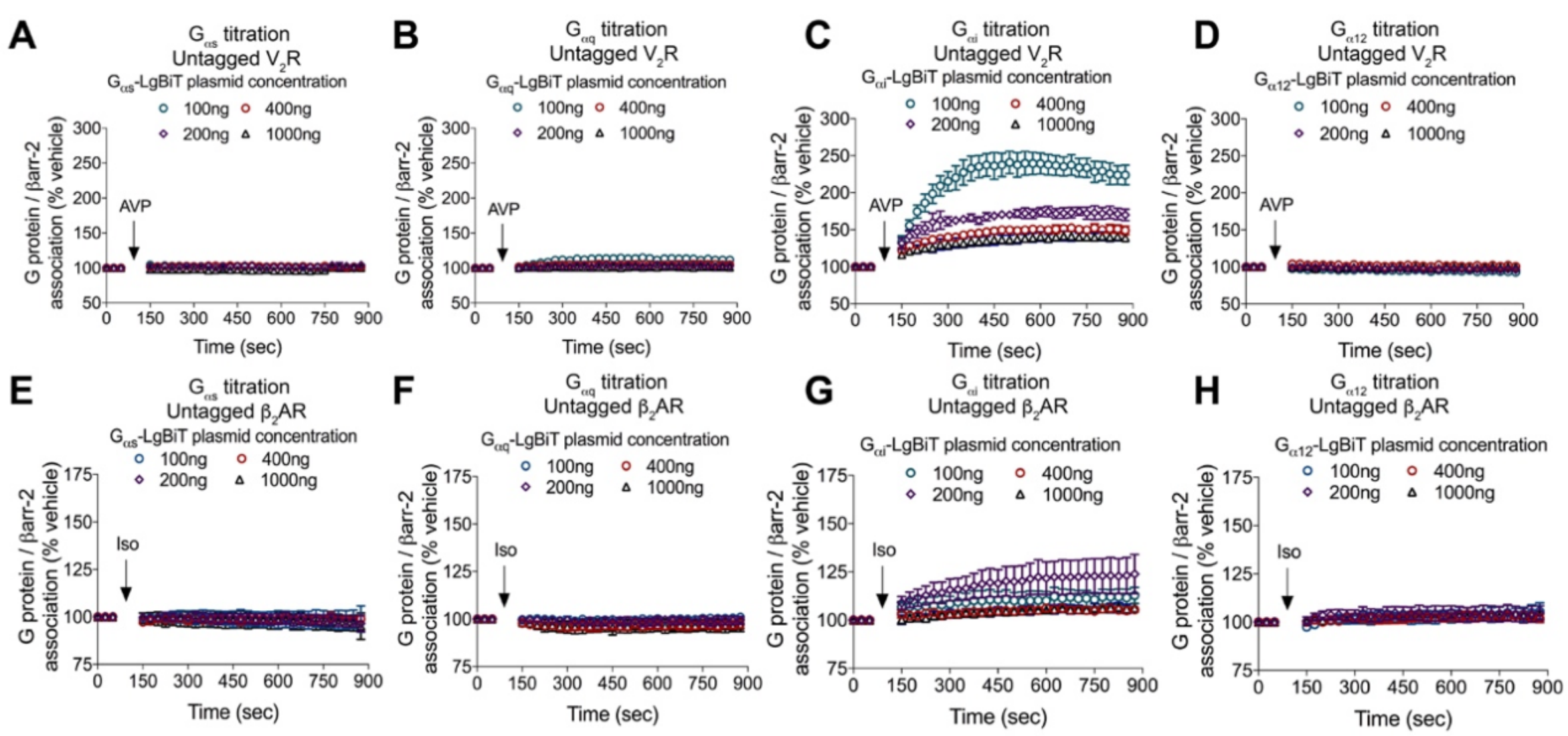
A
Gαs titration
Untagged V2R
Gαs-LgBiT plasmid concentration
100ng
400ng
200ng
1000ng
AVP
G protein / βarr-2 association (% vehicle)
Time (sec)
B
Gαq titration
Untagged V2R
Gαq-LgBiT plasmid concentration
C
Gαi titration
Untagged V2R
Gαi-LgBiT plasmid concentration
D
Gα12 titration
Untagged V2R
Gα12-LgBiT plasmid concentration
E
Gαs titration
Untagged β2AR
Gαs-LgBiT plasmid concentration
Iso
F
Gαq titration
Untagged β2AR
Gαq-LgBiT plasmid concentration
G
Gαi titration
Untagged β2AR
Gαi-LgBiT plasmid concentration
H
Gα12 titration
Untagged β2AR
Gα12-LgBiT plasmid concentration

Adding the LgBiT tag was previously demonstrated elegantly by Asuka Inoue in a 2019 cell paper to not interfere with G protein function (Inoue, Asuka, et al. "Illuminating G-protein-coupling selectivity of GPCRs." ***Cell*** **177.7 (2019): 1933-1947); a screen shot of an example of preserving function is shown in the screen shot below of supplemental figure S2, panels G and H. Importantly, we believe that interactions with beta-arrestin and other G protein families beyond Gi** ***can*** **indeed be observed in certain contexts based on prior reports; however, in our hands the interaction with Gi is by far the most robust in a cellular context and thus the focus of our biochemical validation.**

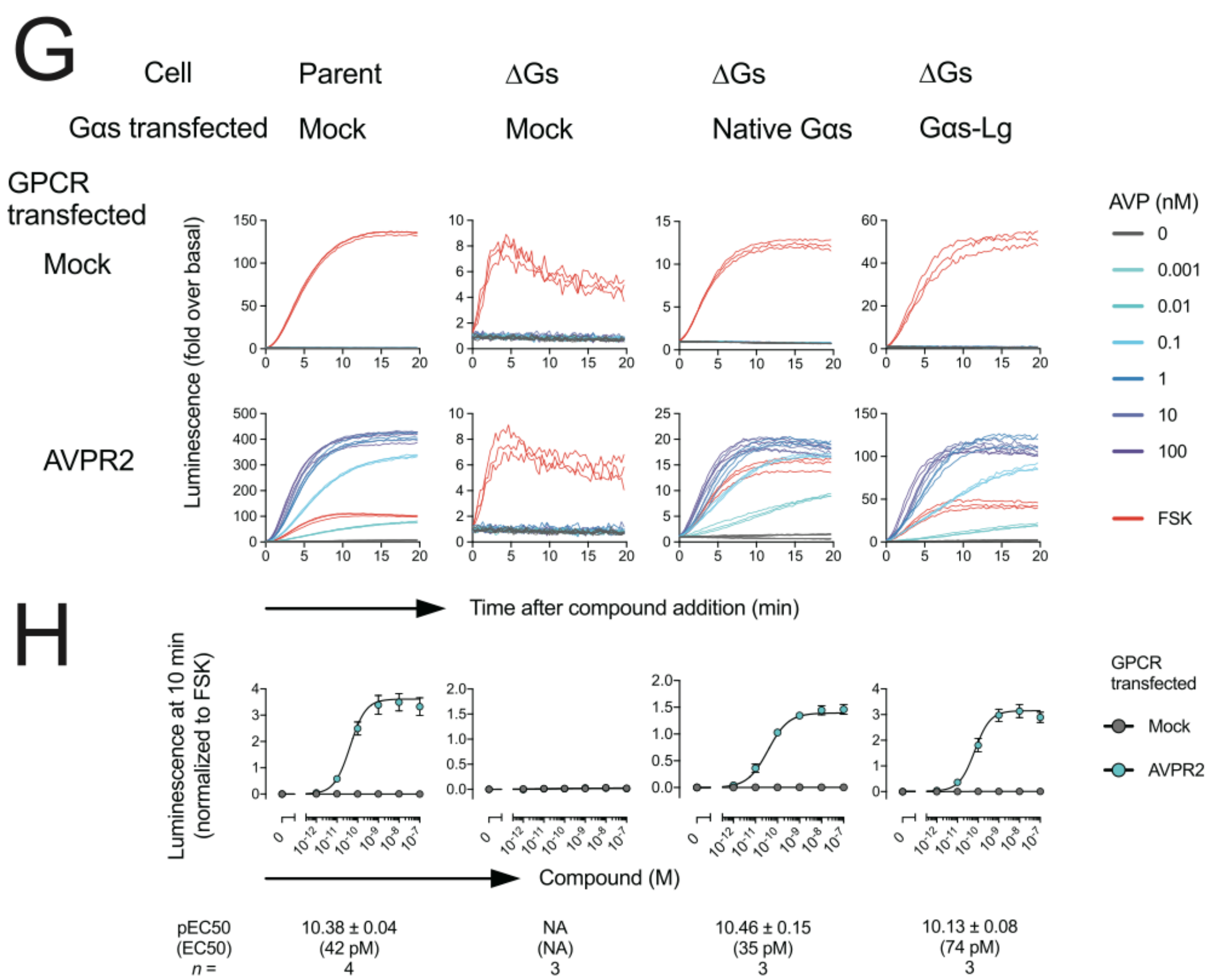

Relatedly, does adding the LgBiT tag differentially change expression levels or function of the Gα proteins? If these experiments were conducted previously and presented elsewhere, it would be helpful if the authors could reference this work.

We have now included western blot data for LgBiT Gα expression (Supplemental Figure 1 panels C,D) and incubation with various concentrations of HiBiT after cell lysis (Supplemental Figure 6 I). These orthogonal approaches to assess G protein levels show that expression differences between families, isoforms,

and/or G protein chimeras do not account for differences in observed association with beta-arrestin.

4. Figure 1C shows Gαi:β-arrestin complex formation following M3R activation by different agonists. Why is the extent of complex formation greater with iperoxo, as compared to carbachol and oxotremorine M? Is the extent of ligand-induced complex formation proportional to the extent of ligand-induced β-arrestin recruitment to the receptor / translocation to the plasma membrane?

Beta-arrestin recruitment to the M3R is indeed greater with iperoxo relative to either Oxo-M or carbachol. We have included the below panel as supplemental figure 1A.

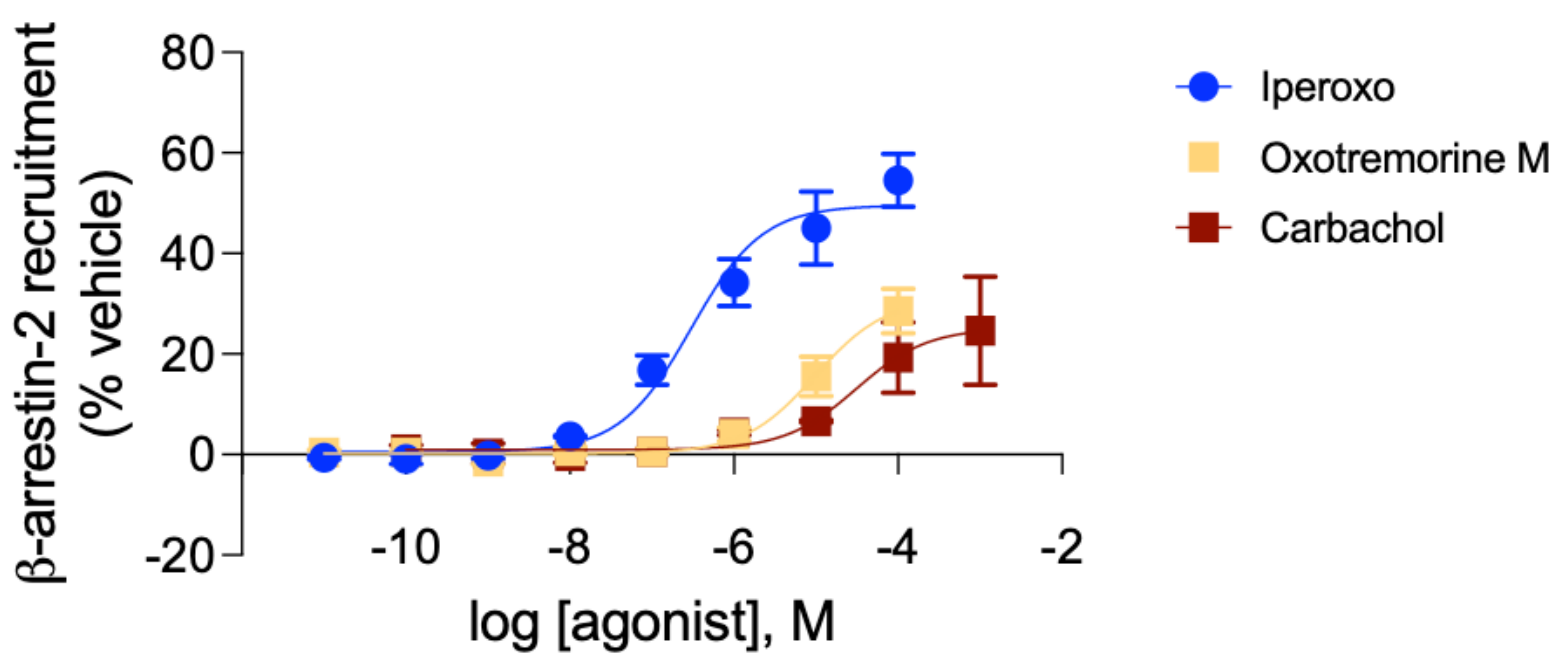

5. In Figure 2, what is the signaling profile of D2R-ARB? It would be helpful if the authors could include this or clearly reference whether the signaling profile has been previously presented. A thorough assessment would include multiple Gi/o G protein family members and a description of what if any G protein activation is retained.

We have provided additional details of the D2R-ARB mutant, previously characterized both in cellular signaling assays and *in vivo* (page 7, lines 147-153). A screen shot of figure 1 from this paper is included for the reviewer, also found here: https://pmc.ncbi.nlm.nih.gov/articles/PMC6378141/

Briefly, the D2R-ARB mutant was created via combining a previously known L123W mutation in transmembrane region 3 of D2R (enhances the potency of agonists to activate D2R) with an R132L mutation located in the DRY motif. The resulting mutant D2R had minimal Gi recruitment (Panel C gray dots and Panel F for other Gi subfamily members in gray bars) and loss of inhibition of cAMP production (Panel G gray dots) while retaining nearly the same beta-arrestin 2 recruitment profile as WT D2R (Panel D gray dots). A screen shot of a pertinent figure from this paper is included below.

Figure 1. An extremely arrestin-biased D2R mutant receptor, D2R(L123W:R132L).

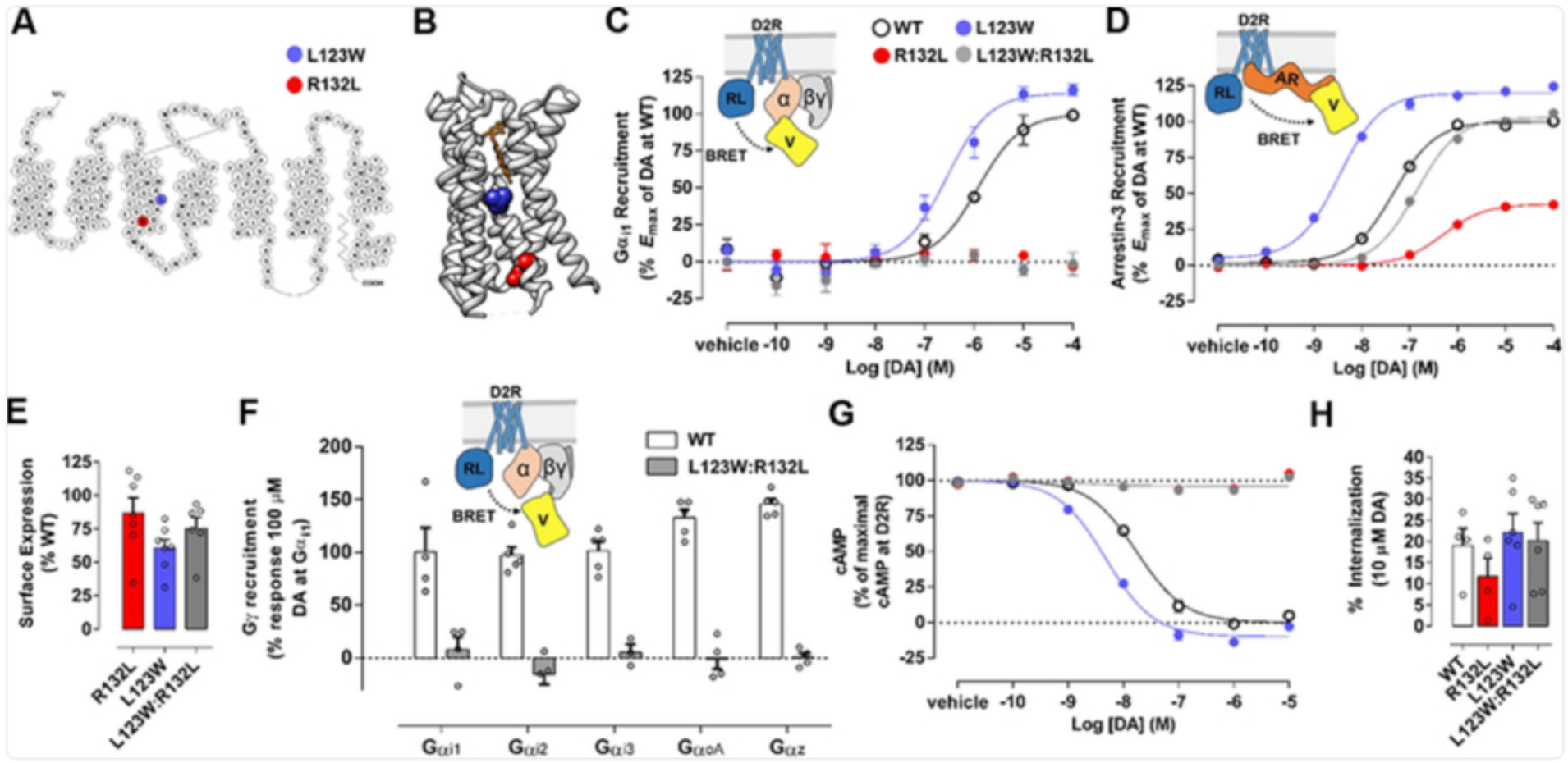

The above referenced experiment is recruitment of G protein to the receptor. In our own hands, we also observed a lack of Gi heterotrimer dissociation following treatment of the D2R-ARB mutant relative to WT control in the TRUPATH G protein dissociation assay, shown in the graph down below using a saturating concentration of 10 mM quinpirole.

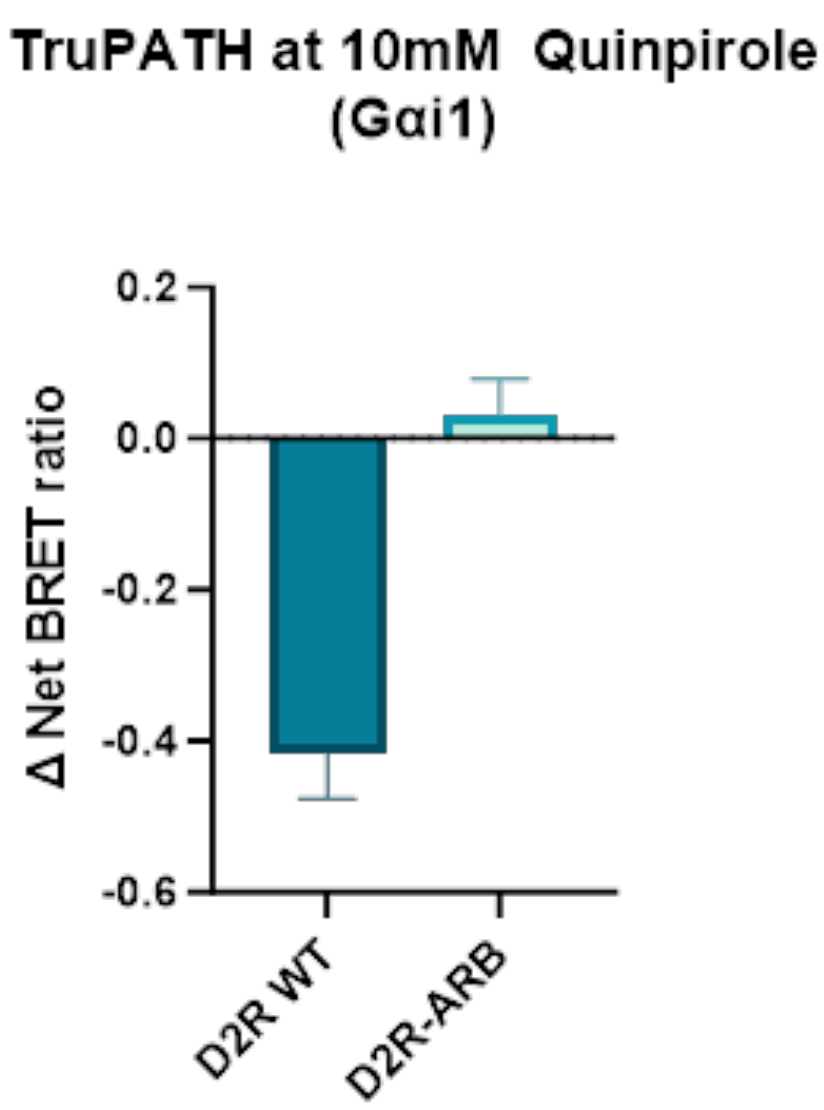

6. In Figure 2, Gq and Gs are presented seemingly as negative controls, but their data are difficult to interpret without understanding how rigorously an association between β-arrestin and these G proteins was investigated. That said, these data do show that the interaction is not tag-driven or non-specific.

In addition to expression data noted above, we have also added a panel probing beta-arrestin-1 association with Gq, Gs, and Gi (supplemental Figure 1B) in HEK293T cells.

7. It would help the reader if the authors could justify their choice of Gq vs Gs for inclusion in the studies presented in Figure 2 (i.e., inclusion of Gq but not Gs in panels A and B and Gs but not Gq in panel C).

For each GPCR, we tested Gαi and the primary G protein that the receptor couples to (Gq for AT1R). Given that D2R primarily couples to Gi and D1R primarily couples to Gs, we chose Gs for comparison here. For ACKR3, no canonical G protein signaling is observed. We have clarified this in the text (page 7, lines 149-153).

8. On page 7, line 145, fentanyl is referred to as a balanced agonist. Others have reported fentanyl to show a preference for β-arrestin activation (Schimid et al. 2017. Volume 171, Issue 5, 16 November 2017, Pages 1165-1175.e13Bias Factor and Therapeutic Window Correlate to Predict Safer Opioid Analgesics). While certainly more balanced than other newly develop MOR ligands, can the authors clarify and contextualize the statement that fentanyl is balanced?

Our initial intent was to highlight the capability for fentanyl, despite its beta-arrestin bias, to robustly activate both Gi and beta-arrestins in comparison to the G-biased ligands for which we observed little to no beta-arrestin recruitment. We have updated the text prevent confusion, only referring to DAMGO as balanced while retaining the fentanyl data for comparison (Page 8, lines 194-195, tracked changes version).

9. In Figure 3, no β-arrestin recruitment is identified with oliceridine. This is in contrast to other reports for this ligand, which show some degree of preserved β-arrestin recruitment. It is not a major concern, as differences in assay design and sensitivity will drive detection, but can the authors comment on differences in the assay platforms that may account for this apparent discrepancy?

Our cellular signaling studies primarily rely on BRET-based approaches for G protein and beta-arrestin signaling. There are several potential methods to assay for beta arrestin recruitment, some of which involve signal-amplification through secondary readouts and/or signal cascades such as in the Presto-TANGO system. Our BRET based approaches do not similarly amplify signal. We have added a line in the discussion (page 14, lines 331-335) noting how differences in assays may account for apparent discrepancies in signaling data between studies and thus assessment of biased signaling.

10. The studies in Figure 3 presumably required expression of the mu opioid receptor (MOR), but this not mentioned in the results, legends, or methods. MOR does appear in the construct list. Could the authors please add information on how these studies were

performed to the Methods and reference MOR expression in the results and/or figure legend?

We have updated our methods section for clarity (page 18, line 423). We have also added MOR to the construct table.

11. Related to the finding that β-arrestin translocation to the plasma membrane is sufficient to promote Gαi:β-arrestin in Figure 4. It would strengthen the authors' argument to demonstrate that rapamycin does indeed drive β-arrestin recruitment to the plasma membrane using an orthogonal assay. Perhaps increased association with a CAAX- or MyrPalm-tethered sensor or via microscopy.

We have included new experimental data that demonstrates recruitment of SmBiT-beta-arrestin2-FKBP recruitment to CAAX-LgBiT via heterodimerization with LynFRB upon stimulating cells with 500 nM rapamycin in Figure 4B.

12. The identification of a direct interaction between Gi1 and β-arrestin1 in Figure 5 is a major finding. This set for studies, however, would benefit from additional explanation and controls. While the representative gel image is nice, density quantification that includes data from multiple biological replicates would increase confidence.

We agree Figure 5 adds important data to the prior characterization of the Gi:beta-arrestin interaction, importantly dissecting the nucleotide status of the G protein. We have included density quantification as assessed by pixel intensity, normalized to beta-arrestin and subtracted the slight non-specific binding of Gai to the resin (new panel, Figure 5B). The methods section has also been updated to reflect this analysis.

Did the authors consider normalizing to β-arrestin? Looking at the relative densities of the Gi input lane and rest of the gel, was the extent of complex formation in line with expectations?

Density quantification calculations are now normalized to beta-arrestin (new Figure 5B). The interaction is clear, but, like many protein interactions with beta-arrestin, seemingly not robust, or requires additional currently unknown proteins, cofactors, and/or cellular conditions to stabilize.

Why switch from β-arrestin-2, with which all cellular complex formation experiments were conduction, to β-arrestin-1 here?

Truncated β-arrestin-1 is far easier to purify in reasonable quantities. Prior supplemental Figure 3 is pasted below for reference; the mAu peak of beta-arrestin-2 is <10% that of beta-arrestin-1 and consistent with reports by other laboratories.

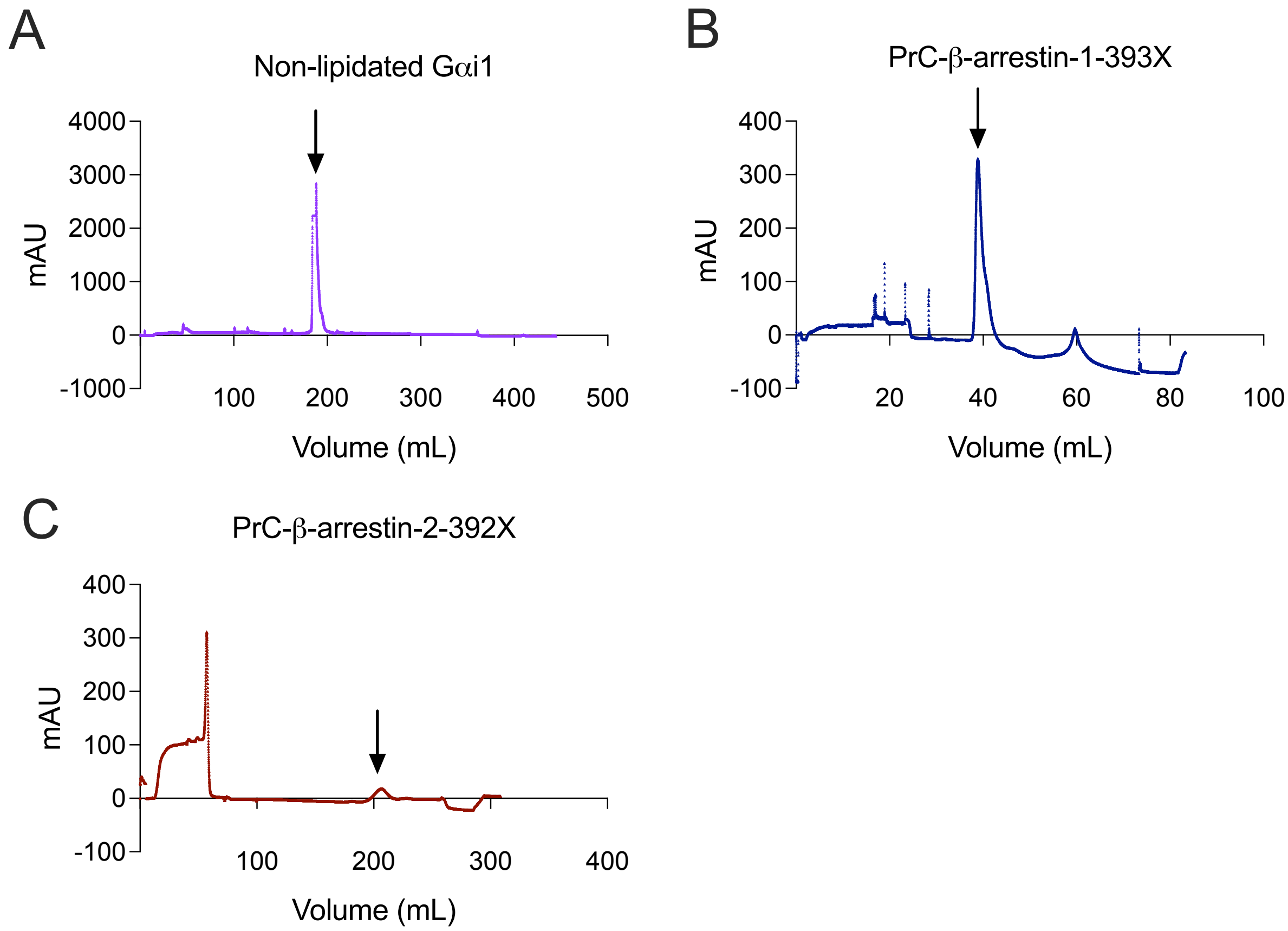

The authors should justify this choice and, ideally, include cellular complex formation studies with β-arrestin-1. And, perhaps most importantly, how does the extent of complex formation with Gi:β-arrestin-1 as compared to Gq: β-arrestin-1 and/or Gs:β-arrestin-1?

We have now included cellular complex formation with β-arrestin-1 (supplemental figure 1B). Similar to β-arrestin-2, Gαi:β-arrestin-1, but neither Gαs or Gαq, interactions are observed.

13. The authors mention that βγ is not required for Gi:β-arrestin complex formation, however, the binding in the pulldowns appear relatively small small. Have the authors considered adding βγ to these pulldowns assays to see if this either increases or decreases binding? This would clarify the role of βγ in this interaction.

We purified non-lipidated βγ (supplemental figure 3D for chromatography trace, showing a monodispersed peak). We have expanded our data in supplemental figure 5 to include pull-downs with βγ and have found no significant change in Gi:β-arrestin association, consistent with our cellular experiments also shown in supplemental figure 5.

14. With Figure 6, I again wonder about attempts to optimize this association assay for

other Gi family members. Also, could the reduction in BRET with these mutants be the result of different complex conformation that results in a greater distance between mKO and Nluc rather than a reduction in total complex formation?

This is possible in principle. However, mutagenesis and HDX data in an accompanying manuscript supports an interaction motif in part within the alpha helical domain.

15. It appears many different species of β-arrestin protein used throughout the work (mouse for BRET, rat or bovine for purification) and that the species did not match that of the G proteins, which were human. The authors should explain these choices.

We used previously validated constructs that are nearly identical; rat and human beta-arrestin 1 are 99.0% identical; murine and human beta-arrestin-2 are 98.05% identical, bovine and human beta-arrestin-2 are 94.8% identical. Notably, bovine beta-arrestin-2 was used for purification as it has been empirically determined to express far better than closely related homologs (some of which were attempted by the authors, and which did not yield workable amounts of protein). Vishnivetskiy, Sergey A., et al. "Arrestin expression in E. coli and purification." ***Current Protocols in Pharmacology*** **67.1 (2014): 2-11. An explanation to this effect have been added in the methods section on page 22.**

16. As I'm sure the authors have considered, it would be fascinating - and further increase the impact of this work - to evaluate the cellular consequences of these alpha helical domain mutations and to determine if these consequences are attributable to a change in the formation or signaling competency of the Gi:β-arrestin complex. In general, evaluating the signaling consequences of complex formation or inhibition would help frame the significance of the interaction.

We agree that further experiments on function beyond our previously published evaluation pERK and cell migration. We plan to use these mechanistic studies to further rigorously dissect functional consequences, but this large undertaking is currently outside the scope of the current manuscript.

Minor
1. The first time FKBP/FRB appears on Page 5, the abbreviation is left undefined.
2. For simplicity, it looks like several panels in Figure 2 could be combined, for example 2A and 2B could be combined into a single panel. 2D and 2E could be combined into a single panel. 2F, 2G, and 2H could be combined into a single panel. This would reduce the total panels in Figure 2 from 8 to 4 and may ease reader interpretation.

We have updated the definition for FKBP/FRB (page 5, line 112 tracked changes version) and have consolidated the panels in Figure 2 accordingly (now panels A-D).

3. Error bars in Figure 5B are SD while for the rest of the figures they are SEM. Could

the authors justify the use of SD for this panel or bring it into alignment with the rest of the figures?

This is now adjusted to SEM.

4. Figure 6 is missing panel numbers.

We have corrected this.

5. The species of the ACKR3 construct is missing from Table 1.

We have updated Table 1 accordingly.

I hope these suggestions, on an already thorough manuscript, will provide relevant feedback to the authors. I remain at the disposal of the editor and the authors for further clarifications.

Reviewer #3 (Remarks to the Author):

Lee, Rajagopal and colleagues provide evidence supporting the ability of beta-arrestin to associate with Gi/o at the plasm membrane, as this group has previously proposed, and they extend the work by demonstrating a specific in vitro association with purified proteins and identifying the G protein N-terminal helix as an important structural determinant of the interaction. The data are generally convincing and the interpretations are reasonable. I'm left being convinced that the interaction demonstrated probably does occur in cells, but unclear about what functional significance this interaction has.

I found the data generally convincing and have only a couple of specific points that would benefit from comment or clarification..

1. The interaction in cells is demonstrated primarily using beta-arrestin-2 but I'm unclear about what interaction is measured in vitro in Fig 5. The figure indicates barr1 in panel A and barr1/2 in B. Can the authors clarify? Is barr1/2 a chimera or what? Can the authors comment on or clarify if there are differences, in cells or in vitro, in Gi/o association with beta-arrestin 1 compared to 2?

Purified beta-arrestin-1 and beta-arrestin-2 were both added to assess if either had an effect on GTP incorporation; neither did. The figure has been updated for clarity "βarr-1 & βarr-2".

We have now conducted Gαi, Gαs, Gαq cellular association assays with beta-arrestin-1. Similar to beta-arrestin-2, only an association with Gai was observed in cells. These data are now included in Supplemental Figure 1B and pasted here for

reference.

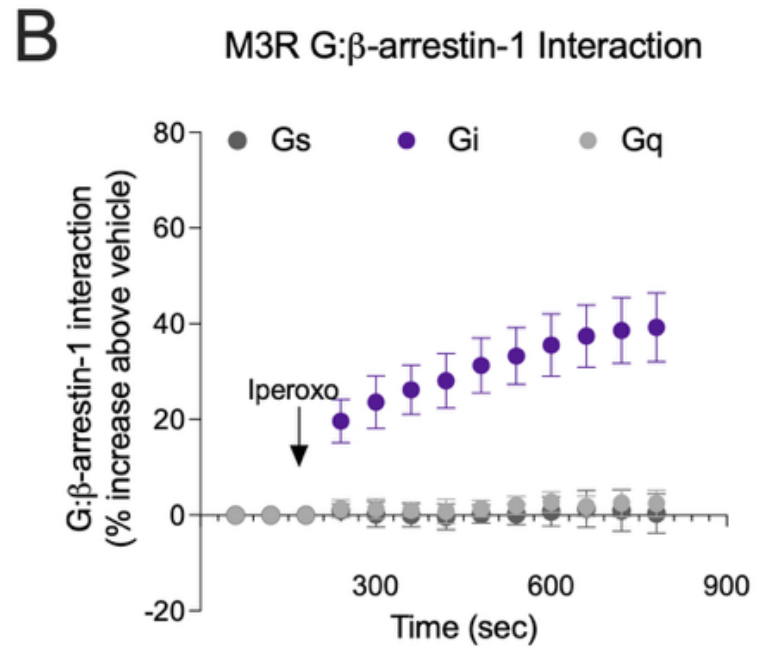

2. I may have missed this in this manuscript or previous publications, but can the authors comment on what functional significance they attribute to the beta-arrestin:G protein interaction. The authors show clearly that the interaction depends on membrane recruitment of beta-arrestin but not GPCR-G protein coupling. They also show recruitment by AVPR2 and I don't believe this receptor couples to Gi/o. What functional consequence do the authors think that this recruitment has?

Our initial 2021 paper was focused on cellular functions Gi:beta-arrestin interaction, which correlated Gi:beta-arrestin association with functions such as cell migration and ERK phosphorylation. Intriguingly, this Gi:beta-arrestin complex could be formed downstream of receptors that do not classically activate Gi, like the vasopressin 2 receptor, as well as others such as the dopamine 1 receptor, which classically couples to Gs. We have expanded the introduction to more fully provide this context (pages 4-5, lines 90-104, tracked changes version). While more specific functional roles remain to be tested, we believe that providing a rigorous characterization of this interaction will facilitate future hypothesis testing.

We thank the reviewers for their continued careful evaluation of our manuscript. Below, we include the reviewers' comments in regular type and our responses in bold.

Reviewer #1 (Remarks to the Author):

The manuscript by Lee et al. has not changed substantially from the initial submission. Instead, the authors argue in their rebuttal that they have performed new experiments and expanded their toolset and make several conceptual advances. I agree that they have shown new data here and have used new tools, but I disagree that the conceptual advances are significant. The main findings as outlined in the abstract are:

"Here we show that G proteins and beta-arrestins can directly interact."

The abstract from their 2021 paper states "we found that GPCRs can catalyze a direct interaction between the Galphai protein subtype and beta-arrestins". Figure 3L in the 2021 paper shows a direct interaction between purified proteins.

"Through utilization of beta-arrestin-biased receptors and artificially enforced beta-arrestin relocalization we demonstrate that recruitment of beta-arrestin to the plasma membrane is sufficient to interact with the G protein Galphai."

Several of the experiments in the previous two papers demonstrate the same concept, including receptor-ligand combinations that do not activate G proteins, PTX, etc. It must also be reiterated that if putting two proteins together in a test tube is sufficient to promote the interaction then concentrating them on a membrane is expected to do so as well.

"Using purified proteins, we show that Galphai directly interacts with beta-arrestin."

See above.

"In addition, we find that Galphai family members differ in their degree of association with beta-arrestin,"

This was amply demonstrated in the original 2021 paper with the same G proteins studied here.

"and that a large degree of this selectivity resides within the alpha helical domain of Galphai."

This is new but given the almost perfect identity of the Galphai isoforms outside of the

alpha helical domain there was little alternative. Loss of function is still shown without reciprocal gain of function, and n is still 3 for this and many other experiments.

The data contained in this manuscript largely confirm this group's previous findings and a few small gaps are filled in, but the overall model hasn't changed significantly. The conceptual advance does not rise to the standard of Nature Communications.

We respectfully disagree with the Reviewer on the advances in the manuscript, which have also been highlighted with the recent acceptance of Duan et al. (DOI: 10.1038/s41467-026-68690-z). Regarding specific points that were raised:

- **The 2021 manuscript did test the effects of pertussis toxin and the biased agonist TRV023. However, some groups have reported residual G protein activity with TRV023. For that reason, we systematically tested other biased agonists and receptors in this work to demonstrate that G protein activation was not required for complex formation.**
- **Similarly, we wanted to test whether beta-arrestin activation was required in the cellular context for complex formation, which we tested with the FKBP:FRB system. While the studies with purified proteins supported a model in which complex formation occurred in the absence of G protein or beta-arrestin activation, that could be artifactual in the setting of high concentrations of protein in a reconstituted system. Demonstrating the sufficiency of beta-arrestin recruitment to the plasma membrane for complex formation in a cellular context addressed this important gap.**
- **We agree that it is likely that the helical domain of the G proteins were mediating interaction with beta-arrestin, but identification of certain critical residues that mediate the interaction was important, as it facilitates future experiments with selective inhibition of the Gai and beta-arrestin interaction while preserving other aspects of Gai activity.**

Reviewer #2 (Remarks to the Author):

The authors addressed most critiques with the addition of control experiments, references to published work, and refinement of the text. As noted previously, the work convincingly demonstrates that the beta-arrestin and Gi interaction is direct and identifies some of its molecular determinants.

A few concerns remain:

1) The largest concern is the lack of functional data demonstrating why this interaction is of biological significance.

We have now included FRB/FKBP rapalog-induced recruitment of β-arrestin-2 to the plasma membrane in total G-protein - knockout cells either rescued with Gαi1 or an empty vector control. Using rapalog over rapamycin in this experiment also reduces

confounding cellular effects of rapamycin/mTOR signaling. This FRB/FKBP system also avoids confounding effects of classical G protein signaling when directly activating a GPCR. In the presence of Gαi, we observed an increase in phosphorylation of Akt Thr 308 following β-arrestin recruitment to the plasma membrane, but no significant difference in conditions lacking Gαi or at Akt Ser 473 (page 9 lines 190-202, Figure 4E-F on page 39, Supplemental Figure 3).

We specifically chose to probe Akt because it is a highly important kinase for cell growth, survival, and cellular movement. Prior work from Marc Caron and colleagues, as well as other groups including our own, have shown that Akt phosphorylation is shaped by β-arrestin (examples: https://pubmed.ncbi.nlm.nih.gov/16051150/, https://pubmed.ncbi.nlm.nih.gov/30401786/).

2) The possibility remains that the effect identified in Figure 6H is driven by differences in construct expression. In lines 255-256 the authors state that "all mutants were tagged with a full-length luciferase (NanoLuc) for use in a NanoBRET assay and to ensure expression differences did not account for variation in signal." However, it is not clear that this expression data is included in the paper. As reduced expression can drive reductions in max delta net BRETs, these data are necessary to rule out expression differences as a confounder in these studies and support the stated conclusion.

We apologize for the confusion; this is misleading as written as not all constructs were tagged with full length nanoluc (although for BRET assays they were in the context we were describing). As most of our comparison data was using LgBiT-tagged G proteins, we transfected cells with these constructs, lysed, and incubated with purified HiBiT to produce the complemented "full length" nanoluc. These are data were in supplemental Figure 6I, and we have now updated to the highest concentration of HiBiT based on feedback (new supplemental Figure 7I). While there is variability in expression of the G protein constructs (especially chimeras, as would be expected), importantly these differences do not correlate with the Gi:Barr complex formation. We have included luminesce data of the full length nanoluc mutants used in Figure 6H below and added onto Supplemental Figure 7J, providing further data that expression differences of the Ga subunits do not explain differences in Gi:Barr complex formation.

3) The new Supplemental Figure 6I plot is challenging to interpret as presented, with the data for the Gs with I Helix 5 entirely obscured.

We have re-graphed at a single high concentration of HiBiT for clarity, as discussed above (supplemental figure 7I). Gs with i-Helix 5 does express to a lower amount but is confirmed to be functional (supplemental Figure 7D).

4) The authors reference a co-submitted manuscript that supports an interaction motif in the alpha helical domain. Without access to this manuscript we cannot assess this claim.

The co-submitted paper was recently accepted and now available online:
https://www.nature.com/articles/s41467-026-68690-z

5) For transparency, the bar graphs in main text Figures 5B and 6I and Supplemental Figures 1D, 5B and 5D should include experimental replicates as symbols.

We have made these adjustments.

Reviewer #3 (Remarks to the Author):

The authors have sufficiently addressed my previous concerns and they have clarified the revised manuscript accordingly.

We thank the reviewer for their time and expertise.

Reviewer #4 (Remarks to the Author):

I co-reviewed this manuscript with one of the reviewers who provided the listed reports. This is part of the Nature Communications initiative to facilitate training in peer review and to provide appropriate recognition for Early Career Researchers who co-review manuscripts.

We thank the reviewer for their time and expertise.
